# Urate-induced epigenetic modifications in myeloid cells

**DOI:** 10.1186/s13075-021-02580-1

**Published:** 2021-07-28

**Authors:** M. Badii, O. I. Gaal, M. C. Cleophas, V. Klück, R. Davar, E. Habibi, S. T. Keating, B. Novakovic, M. M. Helsen, N. Dalbeth, L. K. Stamp, D. Macartney-Coxson, A. J. Phipps-Green, H. G. Stunnenberg, C. A. Dinarello, T. R. Merriman, M. G. Netea, T. O. Crişan, L. A. B. Joosten

**Affiliations:** 1 Department of Medical Genetics, Iuliu Hațieganu University of Medicine and Pharmacy, Cluj-Napoca, Romania; 2 Department of Internal Medicine and Radboud Institute for Molecular Life Sciences (RIMLS), Radboud University Medical Center, Geert Grooteplein 8, 6525 GA Nijmegen, The Netherlands; 3 Department of Molecular Biology, Faculty of Science, Radboud University, Nijmegen, The Netherlands; 4 Department of Rheumatology, Radboud University Medical Center, Nijmegen, The Netherlands; 5 Department of Medicine, University of Auckland, Auckland, New Zealand; 6 Department of Medicine, University of Otago Christchurch, Christchurch, New Zealand; 7 Human Genomics, Institute of Environmental Science and Research (ESR), Wellington, New Zealand; 8 Department of Biochemistry, University of Otago, Dunedin, New Zealand; 9 Department of Medicine, University of Colorado Denver, Aurora, CO 80045 USA; 10 Division of Clinical Immunology and Rheumatology, University of Alabama at Birmingham, Birmingham, AL USA; 11 Human Genomics Laboratory, University of Medicine and Pharmacy of Craiova, Craiova, Romania

**Keywords:** Hyperuricemia, Cytokines, Epigenetics, DNA methylation, Gout

## Abstract

**Objectives:**

Hyperuricemia is a metabolic condition central to gout pathogenesis. Urate exposure primes human monocytes towards a higher capacity to produce and release IL-1β. In this study, we assessed the epigenetic processes associated to urate-mediated hyper-responsiveness.

**Methods:**

Freshly isolated human peripheral blood mononuclear cells or enriched monocytes were pre-treated with solubilized urate and stimulated with LPS with or without monosodium urate (MSU) crystals. Cytokine production was determined by ELISA. Histone epigenetic marks were assessed by sequencing immunoprecipitated chromatin. Mice were injected intraarticularly with MSU crystals and palmitate after inhibition of uricase and urate administration in the presence or absence of methylthioadenosine. DNA methylation was assessed by methylation array in whole blood of 76 participants with normouricemia or hyperuricemia.

**Results:**

High concentrations of urate enhanced the inflammatory response in vitro in human cells and in vivo in mice, and broad-spectrum methylation inhibitors reversed this effect. Assessment of histone 3 lysine 4 trimethylation (H3K4me3) and histone 3 lysine 27 acetylation (H3K27ac) revealed differences in urate-primed monocytes compared to controls. Differentially methylated regions (e.g. HLA-G, IFITM3, PRKAB2) were found in people with hyperuricemia compared to normouricemia in genes relevant for inflammatory cytokine signaling.

**Conclusion:**

Urate alters the epigenetic landscape in selected human monocytes or whole blood of people with hyperuricemia compared to normouricemia. Both histone modifications and DNA methylation show differences depending on urate exposure. Subject to replication and validation, epigenetic changes in myeloid cells may be a therapeutic target in gout.

**Supplementary Information:**

The online version contains supplementary material available at 10.1186/s13075-021-02580-1.

## Introduction

Urate is the end-point metabolite in purine catabolism in humans and is regarded as an alarmin released from disintegrating cells at times of stress or cell death [1, 2]. Higher urate concentration in the serum defines the condition of hyperuricemia, at which point monosodium urate (MSU) crystals can precipitate in peripheral tissues and cause an inflammatory response. Gout is caused by persistent hyperuricemia, a painful inflammatory arthritis caused by the deposition of (MSU) crystals in the synovial cavity [3]. MSU crystals have been shown to induce IL-1β release through activation of the NLRP3 inflammasome [4]. They recruit ASC (Inflammasome Adaptor Protein Apoptosis-Associated Speck-Like Protein Containing CARD) at the inflammasome formation site through the polymerization of tubulin [5]. MSU crystals alone are insufficient for a gout flare and second signals are required to act in synergy with MSU crystals. Such second signals can be pathogen-related ligands such as lipopolysaccharide (LPS) [6], Pam3Cys [7], or sterile stimuli such as fatty acids (e.g., stearate) [8], or the C5a component of the complement [9].

Despite a widely accepted pathogenesis model for gout stemming from long-lasting hyperuricemia that determines the formation of MSU crystals, many questions remain to address the clinical observations of urate-related inflammation[10]. The reasons why not all people with hyperuricemia develop gout, or why some people with MSU crystals in synovial fluid do not show signs of inflammation [11], remain unknown. Genome-wide association studies have identified hundreds of genomic loci associated with serum urate levels and gout [12–15] However, little progress has been made in understanding the genetic control of the progression from hyperuricemia to gout [16]. Large-scale genetic studies are likely to pinpoint additional factors that specifically lead to gout in people with hyperuricemia. Environmental factors (e.g., dietary triggers) [17] can also contribute to inflammation in people with hyperuricemia. Moreover, epidemiological studies suggest that MSU crystals and soluble urate itself also play a role in signaling danger in diseases other than gout: from the low-grade inflammation in aging [18] to common metabolic disorders [19] and cancer [20].

We previously described the priming effects of high concentrations of soluble urate on primary human peripheral blood mononuclear cells (PBMC)s and monocytes, where a shift in cytokine production towards elevated IL-1β concomitant with reduced IL-1Ra could be observed [21]. In addition, we reported that PBMCs of individuals with hyperuricemia produce higher amounts of pro-inflammatory cytokines than normouricemic controls after ex vivo stimulation [21]. This can be reproduced in vitro by pre-treating cells with increasing urate doses followed by washout and re-stimulation with toll-like receptor ligands and MSU crystals. Interestingly, the high proinflammatory capacity coincided with a reduction in IL-1 receptor antagonist (IL-1Ra) production [21], which is at least in part mediated by AKT phosphorylation and autophagy repression in primary human monocytes [22]. Several stimuli exert long-term effects on innate immunity through epigenetic modifications (a process termed trained immunity) [23]. This persistent state of immunological memory can be induced by microbial stimuli such as *Candida albicans* or β-glucan (cell wall component of *C. albicans*) [24, 25], as well as sterile stimuli such as oxidized cholesterol or phospholipids [26, 27].

A recent study performed in patients with gout highlights several differentially methylated loci (DML) with relevance to inflammation [28]. DMLs were found in known gout risk genes and candidate genes (e.g., *SLC2A9*, *ABCC9*), transcription factor genes (*NFATC2* and *MEF2C*), and their regulated gene networks in leukocytes. Pathway analysis of DML suggests gout patients have altered DNA methylation levels of genes involved in both innate and adaptive immunity pathways, with a strong signature for Th17 differentiation and osteoclastogenesis [28].

In the present study, we hypothesize that urate drives persistent proinflammatory effects through epigenetically mediated innate immune memory and that hyperuricemic individuals could have altered epigenetic landscapes in immune cells compared to normouricemic people. We use complementary approaches aimed to establish the molecular basis of urate-mediated proinflammatory status of human monocytes. We show that exposure to urate can have persistent effects in vitro, which is consistent with previous data showing that monocytes of gout patients retain their capacity to produce more cytokines in the absence of hyperuricemia[7, 21]. We identify post-translational histone modifications and DNA methylation as molecular substrates for the effects of hyperuricemia.

## Materials and methods

A detailed version of this section is provided in the Additional file 1.

### Participants

Urate priming experiments were performed in 85 Dutch volunteers from the Human Functional Genomics Project (http://www.humanfunctionalgenomics.org) [29]. Experiments were approved by the Ethical Committee of Radboud University Nijmegen (nr. 42561.091.12). The DNA methylation study was performed in 76 individuals (Table S1) of New Zealand Māori ancestry and was approved by the New Zealand Lower South Health and Disability Ethics Committee (MEC/05/10/130). Patients or the public were not involved in the design, or conduct, or reporting, or dissemination of our research.

### PBMC and monocyte isolation

Human PBMCs were separated using Ficoll-Paque (Pharmacia Biotech). Monocytes were enriched using hyperosmotic Percol solution [30] and were subsequently purified by negative selection using magnetic beads (Miltenyi Biotec).

### Stimulation experiments

Experiments were performed in a culture medium containing RPMI 1640, supplemented with 50 μg/ml gentamicin, 2 mM L-glutamine, 1 mM pyruvate, and 10% human pooled serum following an in vitro urate priming protocol described extensively elsewhere[22].

### Cytokine measurements

Cytokine concentrations were determined in cell culture supernatants using ELISA.

### Animal model

Male 10–12 weeks old C57Bl/6 J mice were purchased from Jackson Laboratories (Bar Harbor, Maine, USA). Uricase was inhibited using oxonic acid and urate was administered according to a previously described protocol [22].

### ChIP-sequencing preparation and analysis

DNA-histone crosslinking was performed using 1% formaldehyde followed by 1.25 mol/L glycine. Chromatin was sonicated using a Diagenode Bioruptor UCD-300 and immunoprecipitated using H3K27ac or H3K4me3 antibodies (Diagenode) and protein A/G magnetic beads. DNA was purified using QIAGEN Qiaquick MinElute PCR purification Kit. Illumina library preparation was done as previously described [31]. Sequencing was performed using Illumina HiSeq 2000.

### DNA methylation analysis

Genomic DNA was isolated from the peripheral blood white cells of 76 individuals of Aotearoa New Zealand Māori ancestry with varying serum urate levels. Genome-wide methylation analysis was performed using Illumina InfiniumMethylationEPIC BeadChips [32].

### Statistical analysis

Cytokine data were analyzed in GraphPad Prism version 8 using Friedmann or Wilcoxon signed rank test. ChIP-sequencing and DNA methylation data were analyzed using R.

## Results

### Urate treatment of human PBMCs in vitro results in a specific and persistent cytokine production phenotype

**Fig. 1:**
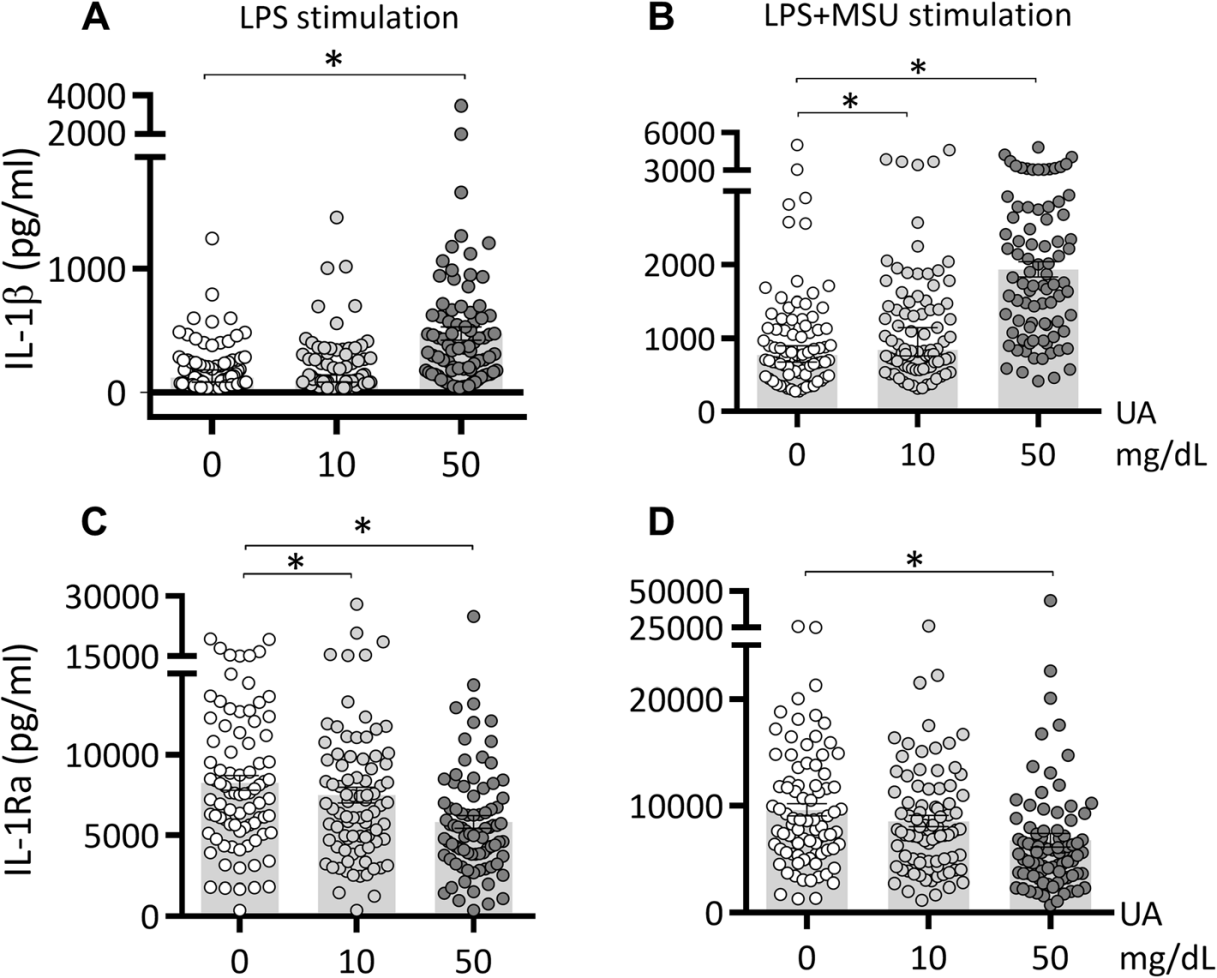
IL-1β and IL-1Ra production after urate priming of PBMCs in vitro. Freshly isolated PBMCs from 85 healthy volunteers were exposed to culture medium (RPMI 1640 supplemented with 10% human pooled serum) in the presence or absence of urate (UA) 10 or 50 mg/dL. After 24 h, urate was removed, and cells were stimulated with LPS 10 ng/mL in the presence or absence of MSU crystals (300 μg/mL). IL-1β (A-B) and IL-1Ra (C-D) were measured in the supernatants of cells, data are representative of 3 independent experiments using a total of 85 different healthy volunteers of the 200FG cohort, graphs depict means+/−SEM. UA, uric acid/urate. *, Friedman test and post-hoc analysis *p* < 0.05

**Fig. 2:**
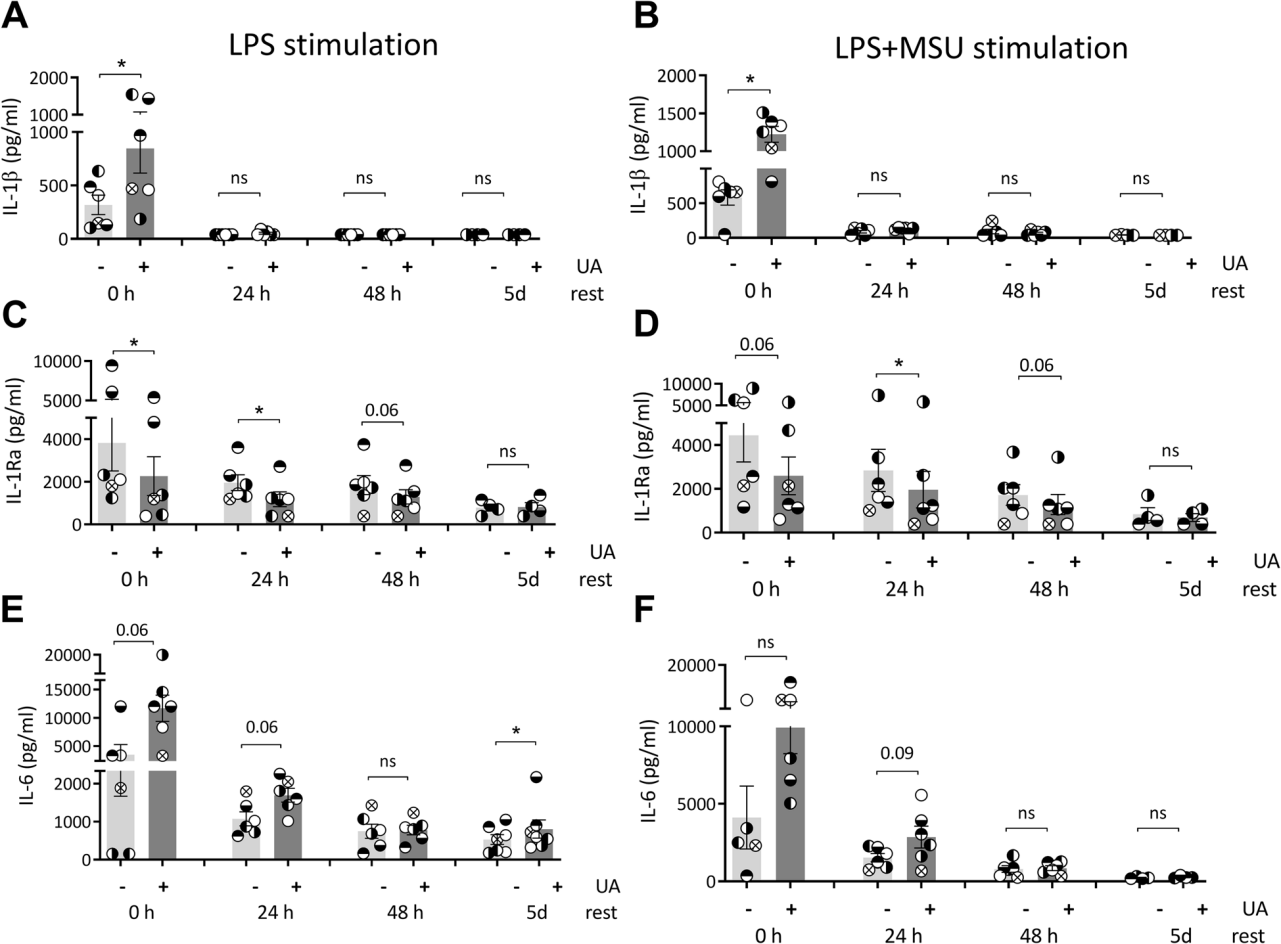
Persistence of urate priming effects in vitro. Freshly isolated PBMCs from 6 healthy volunteers were exposed to culture medium (RPMI 1640 supplemented with 10% human pooled serum) in the presence or absence of urate (UA) 50 mg/dL. After 24 h, urate was removed, and cells were stimulated with LPS 10 ng/mL in the presence or absence of MSU crystals (300 μg/mL). The second stimulation was performed at different times after urate washout: immediately (0 h resting time), or after increasing the number of days of resting in 10% serum RPMI (24 h, 48 h, 5 days). IL-1β (A-B), IL-1Ra (C-D), and IL-6 (E-F) were measured in the supernatants of cells, data are representative for 3 independent experiments and 6 different volunteers, graphs depict individual values with paired samples shown in identical symbols, bars and error bars represent means+/−SEM. UA, uric acid/urate 50 mg/dL. *, Wilcoxon *p* < 0.05

We tested the effects of urate solubilized in culture medium at high concentration (50 mg/dL) or at concentrations similar to in vivo hyperuricemia (10 mg/dL). Both concentrations of soluble urate primed the cells to produce higher IL-1β and lower IL-1Ra production (Fig.1A–D). Next, we investigated whether these priming effects persisted beyond the 24 h period of urate priming. Cells were incubated for 24 h with urate, washed, and thereafter subjected to increasing resting times (up to 5 days) in culture medium before stimulation with LPS (10 ng/mL) and MSU crystals (300 μg/mL). While IL-1β production capacity was strongly diminished after 48 h of culture (Fig.2A, B; 24 h resting periods and onwards), persistent effects were observed for reduction of IL-1Ra (Fig.2C, D) and for induction of IL-6 (Fig. 2E, F).

### Pharmacological inhibition of methyl-transferases inhibits urate effects in an in vivo murine model of gout

**Fig. 3:**
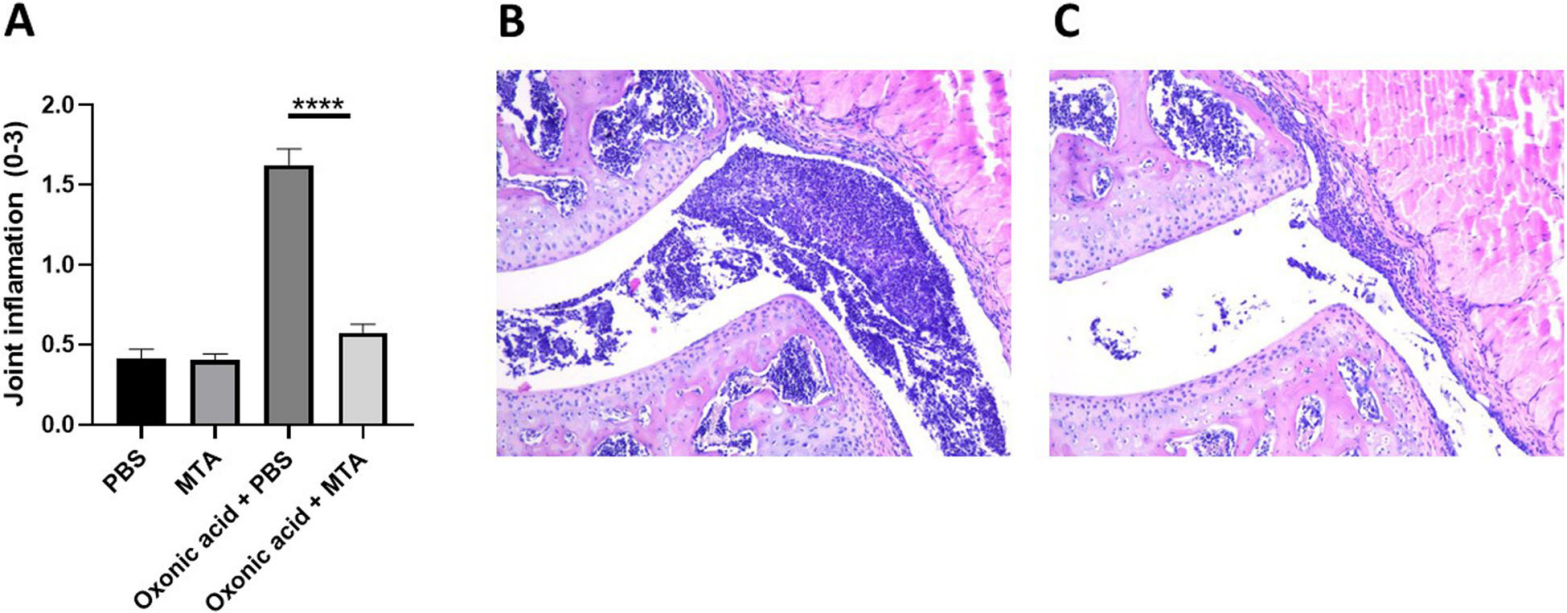
Methyltransferase inhibition limits gout inflammation in mice. Macroscopic (**A**) scores of the knees in mice treated with vehicle control or oxonic acid + urate in the presence or absence of methyl transferase inhibitor MTA (methyl-thio-adenosine) followed by intraarticular injection of MSU+C16:0. Inflammation was scored at 24 h. Histology (H&E staining) of joints treated with MSU+C16:0 in oxonic acid + urate mice (**B**) and in the presence of MTA (**C**)

The broad protein methyl-transferase inhibitor methylthioadenosine (5′-S-methyl-5′-thioadenosine, MTA) was previously shown to inhibit the cytokine production induced by urate in vitro [21]*.* To provide validation in an in vivo model*,* mice were administered exogenous urate in addition to oxonic acid (uricase inhibitor). Acute gout was induced by intraarticular injections with MSU crystals and palmitate (C16:0). Inflammation was significantly enhanced in the oxonic acid group compared with controls as observed by macroscopically scored inflammation (Fig. 3A). The addition of MTA inhibited this effect on the enhanced joint inflammation and histology at 24 h post intraarticular injections (Fig. 3A–C).

### Histone 3 Lysine 4 trimethylation (H3K4me3) or Histone 3 Lysine 27 acetylation (H3K27ac) are mildly affected by urate treatment of human monocytes in vitro

To test whether specific histone modifications are associated with the persistent effects of urate priming, we used chromatin immunoprecipitation coupled with massively parallel sequencing (ChIP-seq) to profile the enrichment of H3 histones trimethylated at lysine 4 (H3K4me3) and H3 histones acetylated at lysine 27 (H3K27ac), two transcriptionally permissive chromatin modifications previously associated with long-term effects of sterile stimul i[26].

**Fig. 4:**
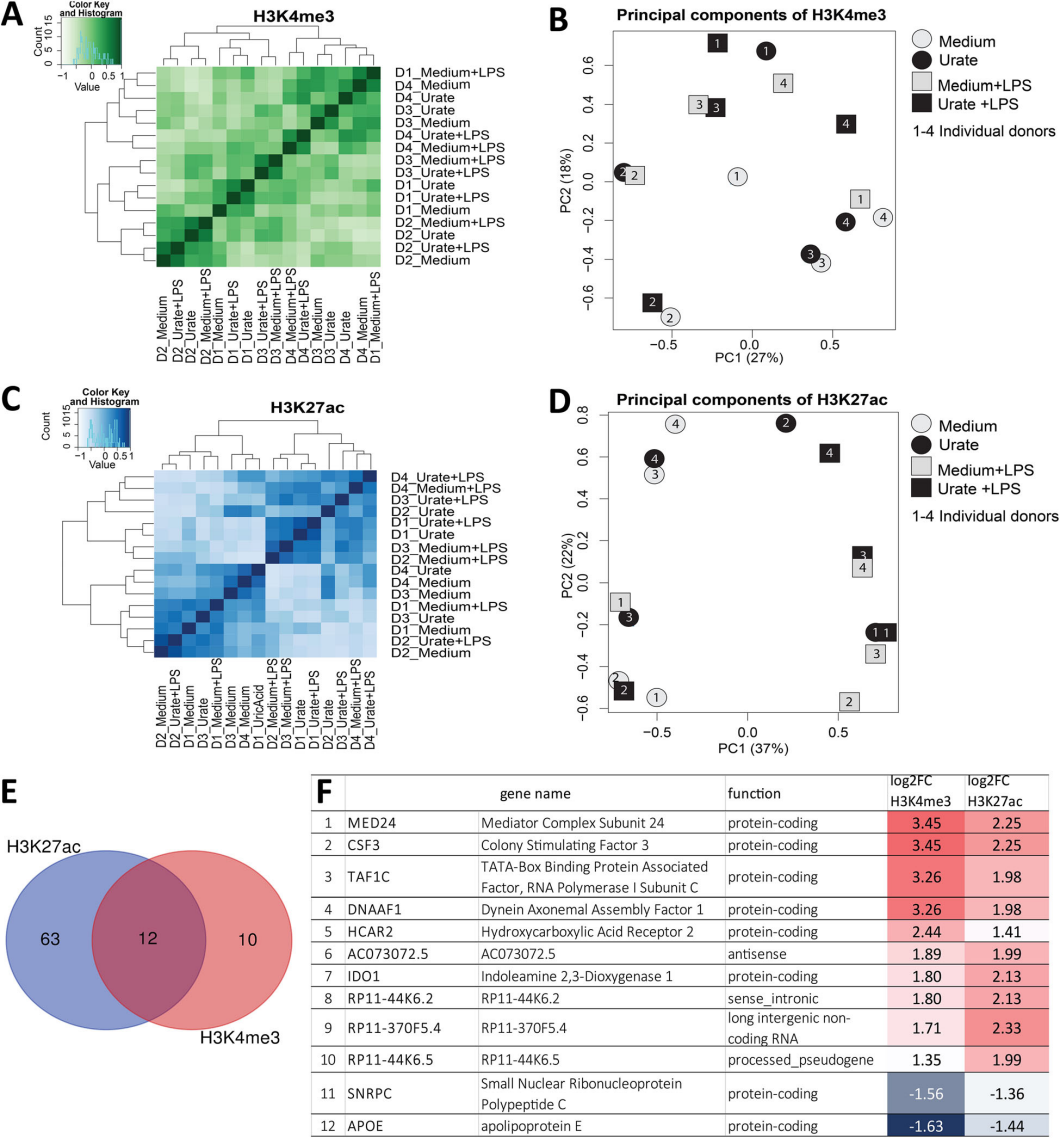
ChIP-sequencing in urate-stimulated monocytes reveals no urate-dependent clustering based on phenotype for H3K4 trimethylation and H3K27 acetylation. Cluster and principal component analysis of datasets obtained on ChIP-sequencing for H3K4me3 (**A**, **B**) or H3K27ac (**C**, **D**) in 4 different donors (labeled D1-4) and 4 different conditions (medium, urate, medium+LPS, urate+LPS). Venn diagram of regions showing differential enrichment of either H3K27ac or H3K4me3 in urate primed cells (**E**) and list of overlapping genes based on histone marks at promoter regions, including log 2 fold change values for each of the two histone marks (**F**)

The ChIPseq analysis was based on all dynamic genes that were identified by comparing all samples to each other. After filtering the data based on these cutoffs, no clustering of stimulated samples was evident to indicate that urate induced genome-wide significant differences on these two histone marks (Fig.4A-D, Fig. S1) Nevertheless, some individual genes, exhibited nominally-significant evidence for variability for H3K4me3 (Table S2) or H3K27ac (Table S3) enrichment at promoter regions. Of these, 12 genes (MED24, CSF3, TAF1C, DNAAF1, HCAR2, ACO73072.5, IDO1, RP11-44 K6.2, RP11-370F5.4, RP11-44 K6.5, SNRPC, and APOE) displayed variability for both histone modifications in urate-primed cells compared to control conditions (Fig. 4E, F).

### DNA methylation profiling reveals candidates for effects of serum urate levels in vivo in humans.

**Fig. 5:**
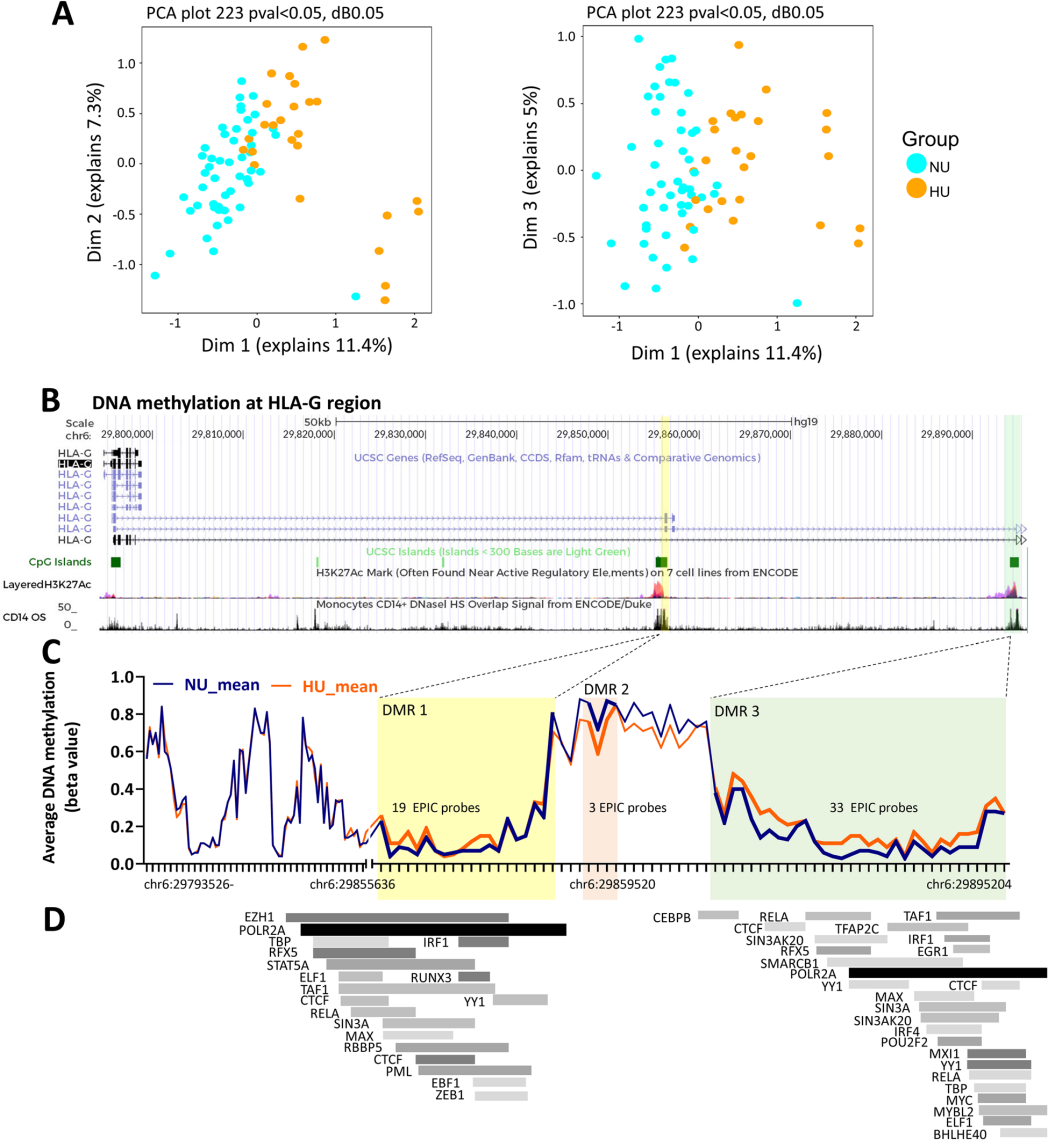
Differential DNA methylation in hyperuricemic versus normouricemic people. Principal component analysis of whole blood DNA methylation data, obtained using Illumina InfiniumMethylationEPIC BeadChips in whole blood of 26 people with hyperuricemia compared to 50 normouricemic individuals (**A**). Differentially methylated regions (DMRs) at the *HLA-G* locus (**B**) and average DNA methylation levels (beta values) for all CpG probes found within *HLA-G* (ticks on the *x*-axis represent individual probes and DNA chromosome position is indicated) (**C**). Transcription factors are known to bind at the highlighted DMR1 and DMR3 regions according to the transcription factor ChIP-seq clusters from ENCODE with factorbook motifs (**D**)

DNA methylation could also function as a basis for urate imprinting as it is a stable epigenetic mark, often associated with long-term gene silencing. In the Aotearoa New Zealand Māori participants, DNA methylation was determined and assessed in whole blood samples of hyperuricemic and normouricemic volunteers. Approximately 850 K CpG sites were studied among the two groups and revealed 223 differentially methylated probes (difference in DNA methylation of at least 5%) (Fig. 5). 23 regions that exhibited significant differential methylation across the two groups of participants were found both in intergenic or intragenic regions of specific genes, one notable example being *HLA-G* (Fig. 5 B-C). Individual differentially methylated probes and differentially methylated regions are listed in Table S4 and Table S5, respectively. An expanded list of candidates was revealed by data analysis of DNA methylation without cell composition correction (detailed information is provided in the supporting information material, Fig. S2, Tables S6 and S7).

## Discussion

In this study, we assess the effects of exposing PBMCs of 85 healthy participants to soluble urate at concentrations of 10 mg/dL or 50 mg/dL in vitro. We show that both concentrations induce a higher IL-1β release and a lower IL-1Ra production in response to subsequent stimulation with LPS or LPS+MSU crystals (Fig. 1). We also describe persistent effects of the treatment with soluble urate in primary myeloid cells that were treated with urate for 24 h, followed by medium removal and stimulation for another 24 h with LPS or LPS+MSU crystals (in the absence of urate). The behavior of monocytes after priming for 24 h with urate has been described previously, demonstrating that the cytokine production capacity of the cells switches to higher IL-1β production and lower IL-1Ra basal concentrations [21], by activating AKT-PRAS40 and inhibiting autophagy [22]. Here, we demonstrate similar effects of urate at 10 mg/dL as high dose urate stimulations (50 mg/dL). Moreover, even after extended resting times between priming and restimulation, modified cytokine levels persisted in primary cells exposed to urate. IL-1Ra remained downregulated after 48 h resting time (Fig. 2C, D). The increase in IL-6 remained present at all timepoints, although statistically significant differences were only observed after 5 days of rest in between stimulations (Fig. 2E, F). This may be due to a limited number of donors and high variability. Significant differences have been previously shown for IL-6 in past reports on urate priming, with direct stimulation at the end of the first 24 h of urate exposure [33, 21]. Other reports have shown that innate immune memory induced by β-glucan, BCG or oxidized LDL particles is best observed after 5 days resting intervals compared to 1 day rest or 3 days rest, possibly in connection with immunometabolism changes (glycolysis induction associated to innate immune memory) [34]. IL-1β induction upon stimulation was, however, markedly reduced at later incubation time points in all conditions (Fig. 1A, B). As opposed to monocytes which express constitutively active caspase-1, in macrophages, the production of IL-1β is more stringently regulated and needs two signals for active IL-1β release: a PRR signal leading to proIL-1β transcription and translation and an inflammasome activator which leads to the proteolysis of proIL-1β into active IL-1β [35]. Our results are in accordance with the restricted IL-1β production in differentiated macrophages after longer in vitro culture of primary monocytes, due to inflammasome (caspase-1) inactivation [35].

Broad protein methylation inhibitor MTA was shown to reverse the urate effect in vitro [21]*.* Here, we show reversal of inflammation in an in vivo model of gout in mice (Fig. 3). The pharmacological inhibition of the uricase enzyme using oxonic acid is a commonly used model to assess hyperuricemia in animal models where the uricase gene functionality is maintained [36, 37]. Previously, we have reported that oxonic acid and urate treatment in mice enhances inflammation triggered by intraarticular injections of palmitate and MSU crystals [22]. In the current study, we show that this effect is reversible in mice that were subjected to MTA treatment prior to intraarticular injections, providing evidence that epigenetic modulators could be potential therapeutic agents for the proinflammatory effects associated to urate exposure. Given the broad effects of MTA, it cannot be excluded that other processes, such as transcription factor methylation, are at play in urate priming.

To further decipher the involvement of histone marks in urate priming and persistence of effects, a genome-wide approach was undertaken in the pursuit of assessing epigenetic modifications globally. Two histone marks were analyzed in the setting of urate priming: trimethylation of lysine 4 of histone 3 (H3K4me3) and acetylation of lysine 27 of histone 3 (H3K27ac). Both marks are associated with activation of gene expression across different cell types [38], have been previously studied in relation to other trained immunity stimuli in recent reports [25, 39], and are known to be present at the promoters of *IL1B* and *IL1RN* in monocytes [40]. The ChIPseq data (Fig. 4) shows a lack of sample clustering based on urate exposure, indicating an absence of genome wide effects for H3K4me3 and H3K27ac in this experiment. Nevertheless, given the small number of participants, targeted approaches at validating the hits which showed evidence of variability are likely to provide new understanding of urate-induced epigenetic effects. Target genes that are relevant for urate-mediated effects were identified in the histone modification datasets. The *IL1B* and *IL1A* genes encoding the IL-1β and IL-1α proinflammatory cytokines show enrichment of the H3K27ac epigenetic mark, which is consistent with previously shown induction of these cytokines by urate in vitro [21]. Twelve genes showed concordant variance in both H3K4me3 and H3K27ac (Fig. 4E, F). Enrichment of both histone marks was highest for *MED24* (mediator complex subunit 24), *CSF3* (colony stimulating factor 3), *TAF1C* (TATA-box binding protein associated factor, RNA polymerase I subunit C), and *DNAAF1* (dynein axonemal assembly factor 1), while both marks were downregulated for *APOE* (apolipoprotein E). APOE has been previously associated to gout and hyperuricemia as a potential link with hypertriglyceridemia, a common finding in patients with gout [41]. APOE has also been reported to coat MSU crystals and inhibit MSU-induced inflammatory signaling [42]; therefore, the finding that urate exposure can lead to a reduction in H3K4me3 and H3K27ac (and, consequently, reduced chromatin accessibility) at the *APOE* locus suggests that this effect could be a point of study for the progression from hyperuricemia to gout. The validation of H3K27ac or H3K4me3 enrichment in response to lower concentrations of urate or in patients with hyperuricemia would be a useful next step. The fact that no differences are observed for LPS stimulation in our dataset is intriguing and could be attributable to the in vitro differentiation of the monocytes into macrophages in the presence of serum-supplemented culture medium. Serum is known to downregulate CD14 expression and to induce the release of CD14 in the extracellular space [43]. Moreover, in vitro culture of monocytes for 24 h in standard conditions is reported to lead to the irreversible loss of MD2 activity [44]. Taken together these data suggest that serum-derived macrophages are less responsive to LPS due to CD14 and MD2 downregulation in standard culture conditions, which may explain the lack of significant alteration in the studied histone marks in response to the short 4 h stimulation with LPS.

We also studied the possibility of DNA methylation involvement in the proinflammatory effects associated to hyperuricemia in a sample set of New Zealand Māori ancestry [28]. The Māori population has 2–3-fold higher risk of gout compared to the population of European descent[45], likely contributed to by genetic susceptibility alleles that have increased in prevalence during the Pacific ancestral migrations, through mechanisms that are still debate d[46]. By comparing DNA methylation status in hyperuricemic versus normouricemic volunteers within this cohort, 223 differentially methylated probes and 23 differentially methylated regions were identified in the vicinity of genes. Interestingly, three DMRs were found to be present at the *HLA-G* locus (human leukocyte-associated antigen, class I, G) (Fig.5). Two of the *HLA-G* DMRs coincided with H3K27ac enrichment peaks (Fig.5B), CD14+ monocyte DNAse I accessibility peaks (Fig. 5B) and binding sites for several transcription factors (Fig. 5D) (www.encodeproject.org), which is indicative of potential functional effects of the DNA methylation variance observed at these sites. HLA-G is a HLA-class Ib molecule with immunomodulatory properties across several tissues, which has recently been suggested to limit the progression of autoimmune and autoinflammatory disorders (extensively reviewed in [47]) and could be a promising target to study in gout.

Other candidates highlighted by the presence of two DMPs in the vicinity of the gene (Table S4) were *IFITM3* (interferon-induced transmembrane protein 3) or *PRKAB2* (AMP-activated protein kinase subunit beta 2, AMPK-β2). Genes in the interferon signaling pathway have previously been reported to be differentially expressed (upregulated) in whole blood of healthy individuals administered rasburicase compared to placebo [48]. In monocytes pre-treated with urate, transcriptomic analysis revealed downregulation of genes associated to GO term “Influenza A,” which includes several interferon signaling-related genes [22]. Moreover, type I interferons are known to inhibit STAT1 signaling and inflammasome activation [49]; hence, urate-induced downregulation of interferon signaling could play a role in escalating IL-1 production and release. AMPK-β2 is a regulatory subunit of AMP-activated protein kinase (AMPK). AMPK activation was shown to limit MSU crystal-induced IL-1β production and to drive anti-inflammatory macrophage M2 polarization [50]. DNA hypomethylation at the *PRKAG2* gene body (AMPK subunit gamma 2) was reported in gout patients compared to controls [50]. Furthermore, PRKAG2 is one of the loci associated with hyperuricemia [12] and gout [51]. Our data suggest that urate exposure could modulate AMPK and interferon signaling pathways via DNA methylation in hyperuricemic people.

This report has the limitation of having studied a small number of donors for the assessment of genome-wide epigenetic modifications (monocytes of 4 donors and stimulations for ChIP-sequencing, or 26 versus 50 volunteers for the DNA methylation study). Variation in histone modification or DNA methylation in no gene was found to be experimentally-wide significant between urate-exposed and control. Nevertheless, there is evidence of variation correlated to urate exposure for all these epigenetic mechanisms in candidate genes. Further studies using larger sample sizes or targeted approaches based on these initial candidates are needed in order to find statistically significant effects. Since this report describes consequences of higher than normal urate levels, we cannot exclude that any of these effects could also be driven by the precipitated form of urate. For the in vitro experiments, using polarized light microscopy, we were not able to observe urate crystals formed during the 24 h of exposure time for the described experimental conditions. Validating the changes in the histone mark landscape at lower concentrations of urate or in patients with hyperuricemia would be an important next step. Finally, the current study lacks mRNA data to show transcriptional regulation of cytokine genes in response to urate after differential resting periods. A previous report by our group showed that the gene expression of *IL1B*, *IL1RN*, and *IL6* follows the same trend as the protein levels at 24 h [22]. Further assessment of the transcriptome of samples at later time-points after the initial encounter with urate would help understand the extent of persistence of the transcriptional programme induced by urate.

## Conclusions

We have generated datasets involving epigenomic and functional immunological experiments to investigate potential major mechanisms involved in the urate priming of myeloid cells. Based on complementary methods we show that epigenetic changes are likely to play a role in mediating the persistent effects of urate exposure on innate immune cells. Our study shows that high levels of urate can persistently alter the cytokine production capacity of primary PBMCs in vitro, leading to increased IL-1beta and IL-6 production and reduced levels of IL-1 receptor antagonist (IL-1Ra). Uricase inhibition in mice led to higher inflammation scores upon intraarticular injection of MSU crystals and palmitate, and this effect was reversed by methyltransferase inhibition. We present here evidence that histone modifications (H3K4me3 or H3K27ac) and DNA methylation show differences in response to high urate exposure and provide potential candidates of differentially regulated targets. The differences in epigenetic regulation may provide a new understanding and possibility for intervention in urate-dependent inflammatory responses as well as in the progression from hyperuricemia to gout.

## Supplementary Information

**Additional file 1.**

## Data Availability

ChIP-sequencing and cytokine data used for this manuscript will be made available to readers upon request. DNA methylation data cannot be made available due to ethical reasons.

## Abbreviations

ABCC9: ATP-binding cassette, sub-family C member 9
ACO73072.5: Long non-coding RNA, LOC541472
AKT: Serine-threonine kinase
AMPK: Adenosine 5′-monophosphate (AMP)-activated protein kinase
APOE: Apolipoprotein E
C16:0: Palmitate
C5a: Complement component 5a
ChIP-seq: Chromatin immunoprecipitation sequencing
CpG: 5′—C—phosphate—G—3'
CSF3: Colony stimulating factor 3
DML: Differentially methylated loci
DNAAF1: Dynein axonemal assembly factor 1
ELISA: Enzyme-linked immunosorbent assay
H3K4me3: Histone 3 lysine 4 trimethylation
H3K27ac: Histone 3 lysine 27 acetylation
HCAR2: Hydroxycarboxylic acid receptor 2
HLA-G: Human leukocyte antigen-G
IDO1: Indoleamine 2,3-Dioxygenase 1
IFITM3: Interferon-induced transmembrane protein 3
IL-1β: Interleukin-1 beta
IL-1Ra: Interleukin-1 receptor antagonist
IL-6: Interleukin-6
LPS: Lipopolysaccharide
MED24: Mediator complex subunit 24
MEF2C: Myocyte enhancer factor 2C
MSU: Monosodium urate
MTA: 5′-Methylthioadenosine
NFATC2: Nuclear factor of activated T cells 2
NLRP3: NLR family pyrin domain containing 3
Pam3Cys: N-Palmitoyl-S-[2,3-bis(palmitoyloxy)-(2RS)-propyl]-[R]-cysteinyl-[S]-seryl-[S]-lysyl-[S]-lysyl-[S]-lysyl-[S]-lysine
PBMC: Peripheral blood mononuclear cells
PRAS40: Proline rich Akt substrate, 40 kDa
PRKAB2: Protein kinase AMP-activated non-catalytic subunit beta 2
RP11-44 K6.2, RP11-370F5.4, RP11-44 K6.5: Long non-coding RNAs
RPMI: Roswell Park Memorial Institute medium
SLC2A9: Solute carrier family 2 member 9
SNRPC: Small nuclear ribonucleoprotein polypeptide C
Th17: T helper 17 cell
TAF1C: TATA-Box Binding Protein Associated Factor, RNA Polymerase I Subunit C
