# Supplementary material for: Urate-induced epigenetic modifications in myeloid cells
Source: Arthritis Res Ther. 2021 Jul 28;23:202. doi: 10.1186/s13075-021-02580-1 (PMC8317351; doi:10.1186/s13075-021-02580-1)
Supplement: Supplementary file 1 — Additional file 1. [file 13075_2021_2580_MOESM1_ESM.docx]

**Urate – induced epigenetic modifications in myeloid cells**

**ADDITIONAL FILE 1**

**MATERIALS AND METHODS**

**Participants**

Venous blood was drawn from the cubital vein of Dutch participants without gout into EDTA tubes. Urate priming experiments were performed in freshly isolated PBMCs of 85 healthy volunteers of Dutch nationality from the 200 Functional Genomics (200FG) cohort in the Human Functional Genomics Project (<http://www.humanfunctionalgenomics.org>)[1]. Experiments requiring large amounts of cells were performed using cells isolated from buffy coats after overnight storage at room temperature (Sanquin blood bank, Nijmegen, The Netherlands). Stimulation experiments were approved by the Ethical Committee of Radboud University Nijmegen (nr. 42561.091.12) and were conducted according to the principles of the Declaration of Helsinki. The DNA methylation study was approved by the New Zealand Lower South Health and Disability Ethics Committee (MEC/05/10/130). The study included 76 individuals of New Zealand Māori ancestry (Table S1), 38 men and 40 women, with a range of serum urate values (average 0.37 mmol/L, +/- standard deviation 0.12 mmol/L, range 0.6 mmol/L), who for further analysis were segregated into a hyperuricemia group (0.42 mmol/L or higher) (n=26) or a normouricemia (n=50) group. All participants provided written informed consent. Patients or the public were not involved in the design, or conduct, or reporting, or dissemination of our research

**Reagents**

Urate, lipopolysaccharide (LPS, *E. coli* serotype 055:B5), and 5'-S-methyl-5'-thioadenosine (methylthioadenosine, MTA) were purchased from Sigma. LPS was subjected to ultra-purification before cell culture experiments. Monosodium urate (MSU) crystals were prepared inhouse as previously described[2]. Pharmacological epigenetic inhibitors were purchased from Tocris, Bio-techne (Table S1).

**PBMC and monocyte isolation**

Human PBMCs were separated using Ficoll-Paque (Pharmacia Biotech) and suspended in culture medium RPMI (Roswell Park Memorial Institute 1640). Monocytes were enriched using hyperosmotic Percol solution[3] and were subsequently purified by negative selection using the Pan Monocyte Isolation kit (Miltenyi Biotec) according to the manufacturer’s instructions. This led to a cell suspension containing monocytes with 85-95% purity.

**Stimulation experiments**

Experiments were performed in culture medium containing RPMI 1640, supplemented with 50 µg/ml gentamicin, 2 mM L-glutamine, 1 mM pyruvate and 10% human pooled serum following an in vitro urate priming protocol described extensively elsewhere[4]. For ChIP-seq experiments, 5x10^6 human monocytes were seeded in Petri dishes (Corning) and were primed for 20 hours followed by addition of medium or LPS 10 ng/ml for another 4 h. Cells from all donors used for ChIP-seq were also used in a separate experiment conducted in parallel for RNA-seq assessment and for control experiments to test if the expected urate priming phenotype was present. The experimental setup, purity of cells and cytokine control experiments were as previously described[4]. For the *in vitro* experiments, using polarized light microscopy, we were not able to observe urate crystals formed during the 24 hours of exposure time for the described experimental conditions.

**Cytokine measurements**

Cytokine concentrations were determined in supernatants of cell culture using specific sandwich ELISA kits for interleukin-1β (IL-1β), IL-1Ra (receptor antagonist) (R&D Systems), and IL-6 (Sanquin) according to the manufacturer’s instructions.

**Animal model**

Male 10-12 week old C57Bl/6J mice were purchased from Jackson Laboratories (Bar Harbor, Maine, USA), a total of five males were selected per group. The experiments were approved by The Institutional Animal Care and Use Committees of the University of Colorado Denver, Aurora, CO (protocol #0035) and were conducted according to the principles of the Declaration of Helsinki. Uricase was inhibited using oxonic acid and urate was administered in order to increase serum urate levels according to a previously described protocol[4]. At Day 0, mice were given oxonic acid orally 140 mg/kg, 2 times/day (in the morning and in the evening). Each administration was followed after 2 hours by urate 4 mg/kg, intraperitoneally, in the presence of MTA 30 mg/kg or equivalent volumes of vehicle control. At Day 1, the same treatment as Day 0 was performed, together with induction of gouty arthritis at noon. Gouty arthritis was induced by intra-articular injection (i.a.) of 300 μg MSU crystals and 200 μM palmitic acid (C16) in a volume of 10 μl phosphate buffered saline (PBS) as previously described in both the right and left knee joints of each mouse [2–5]. 24h after injection, mice were sacrificed, knees were macroscopically scored for joint thickness after removal of skin resulting in 10 joints in total, 5 mice per group (scores ranging from 0 to 3), followed by harvesting of joints for histology (only the right knee joint of the mice). Histology was performed as previously described[2].

**ChIP sequencing preparation and analysis**

Immediately after cell isolation and after stimulation, samples were subjected to DNA-histone crosslinking by treatment with 1% formaldehyde for 10 minutes followed by treatment with 1.25 mol/L glycine for another 3 minutes. For cultured cells, floating medium and cells were removed followed by three cycles of scraping and recovering adherent fixated cells (2.4-4.8 x 10^6 cells could be recovered from 5x10^6 plated monocytes). Samples were stored at 4°C in PBS containing complete phosphate inhibitor cocktail until sonicated and subjected to chromatin immunoprecipitation as previously described[6]. Briefly, fixed cell preparations were sonicated using a Diagenode Bioruptor UCD-300 for 3x 10min (30s on; 30s off). 67μl of chromatin (1 million cells) was incubated with 229μl dilution buffer, 3μl protease inhibitor cocktail and 0.5-1μg of H3K27ac or H3K4me3 antibodies (Diagenode) and incubated overnight at 4°C with rotation. Protein A/G magnetic beads were washed in dilution buffer with 0.15% SDS and 0.1% BSA, added to the chromatin/antibody mix and rotated for 60 min at 4°C. Beads were washed with 400μl of buffer for 5min at 4°C with five rounds of washes. Chromatin was eluted using elution buffer for 20min. Supernatant was collected, 8μl 5M NaCl, 3μl proteinase K were added and samples were incubated for 4hr at 65°C. Finally, samples were purified using QIAGEN; Qiaquick MinElute PCR purification Kit and eluted in 20μl EB. Detailed protocols can be found on the Blueprint website (http://www.blueprint-epigenome.eu/UserFiles/file/Protocols/Histone_ChIP_May2013.pdf).

Illumina library preparation was done as previously described[6], using the Kapa Hyper Prep Kit. Samples were purified using the QIAquick MinElute PCR purification kit and 300bp fragments selected using E-gel. Correct size selection was confirmed by BioAnalyzer analysis. Sequencing was performed using Illumina HiSeq 2000 machines and generated 43bp single end reads. Sequence reads were aligned to human genome assembly hg19 (NCBI version 37) using bwa. Duplicate reads were removed after alignment with Picard tools. For peak calling the BAM files were first filtered to remove the reads with mapping quality less than 15, followed by fragment size modeling (https://code.google.com/archive/p/phantompeakqualtools/) and MACS2 (https://github.com/taoliu/MACS/) was used to call the peaks. For each histone mark dataset, the data were normalized using the R package DESeq2 and then pair-wise comparisons were performed (fold change 3, adjusted p-value< 0.05 and reads per kilo base per million mapped read ≥ 2) to determine the differentially expressed genes per condition. The results from all possible pairwise comparisons (within each condition and similar time points across all conditions per mark) were pooled and merged to define the dynamic set of enriched regions.

**DNA methylation analysis**

Genomic DNA was isolated from peripheral blood of 76 individuals of New Zealand Māori ancestry with varying serum urate levels. Genome-wide methylation analysis was performed using Illumina InfiniumMethylationEPIC BeadChips (referred to from now as ‘EPIC array’)[7]. The EPIC array measures DNA methylation level at more than 850,000 CpG sites (referred to as ‘EPIC probes’), and covers all gene promoters, gene bodies and ENCODE-assigned distal regulatory elements[8].

Raw IDAT files were processed and analysed using the MissMethyl and minfi packages for R[9, 10], both available from Bioconductor[11]. Samples were checked for quality, with all samples remaining for analysis (all with a mean detection p-value of <0.01). A total of 50 controls and 26 hyperuricemia samples were used for analysis.

Data were normalized for both within and between array technical variation using SWAN (Subset-quantile Within Array Normalization)[12]. A total of 798,740 probes were used for analysis after removal of poor probes, probes that overlap SNPs with MAF>0%, and cross hybridising probes . Cell composition was determined using the estimateCellCounts tool, with the ‘Blood’ reference data used for adult peripheral blood analysis[13]. Differential methylation analysis by linear regression modelling was performed using limma[14]. Differential methylation analysis with and without cell composition correction was performed.

Differentially methylated probes (DMPs) were identified as those having a p-value of <0.05 and a change in methylation (delta beta or Δβ) of ≥5%. Differentially methylated regions (DMRs) were identified using the DMRcate tool[15].

**Statistical analysis**

Cytokine data were analyzed using GraphPad Prism version 8. The differences were analysed using Friedmann or Wilcoxon signed rank test after testing for distribution normality. Data were considered statistically significant at a p-value<0.05. Data is shown as individual or cumulative results of levels obtained in all volunteers (means +/- SEM). ChIP sequencing and DNA methylation data were analyzed using R.

**RESULTS**

**ChIP-Seq analysis in freshly isolated monocytes at day 0 displays a marked clustering feature compared to all stimulated samples obtained at day 1.**

To assess whether the ChIP-seq data showed differences in freshly isolated monocytes compared to cultured monocytes, samples before and after culturing conditions were included in the analysis. The data was reanalyzed with an extra negative control sample for each of the four donors, which was prepared from freshly isolated monocytes, at day 0, before culture. The analyses performed using day 0 samples and day 1 samples (with the 4 conditions described – RPMI or urate primed cells stimulated with either control medium or LPS) revealed distinct features of the samples obtained at day 0 compared to all samples obtained at day 1.

**DNA methylation profiling without cell composition correction reveals an expanded list of candidates for effects of urate *in vivo* in humans.**

To obtain additional information regarding the DNA methylation patterns associated to hyperuricemia, DNA methylation data were also analysed without correction for cell composition. This second analysis revealed 676 differentially methylated probes (which consisted of a difference in DNA methylation of at least 5%) and 55 differentially methylated regions (Tables S6 and S7, respectively). The *HLA-G* locus was consistently identified by both types of analyses. Regions that depicted significant differential methylation over the two groups of patients were found in intergenic or intragenic regions of certain genes, of which a list of candidate genes are shown in Fig.6A. One interesting example with possible roles for the modulation of the IL-1β/IL-1Ra pathway is *SOCS3,* which exhibited three significantly differentially methylated CpG probes that were found intragenically (Fig.S2) and which showed higher methylation in hyperuricemic compared to normouricemic patients (Fig.S2 D). Publicly-available data from the ENCODE database show that this region includes transcription factor binding sites that could be influenced by variable methylation and chromatin accessibility (Fig.S2 E).

**DISCUSSION**

Recently, the use of the cell composition correction (Houseman correction,[16]) for whole blood DNA methylation data was challenged due to the possible violation of the no multicollinearity assumption in statistical regression models[17]. This possibility is of particular relevance for studies in which the assessed outcomes are represented by inflammatory phenotypes, because inflammation can be associated to cell-type composition variation[17]. For this reason, in this study, DNA methylation was determined and assessed between hyperuricemic and normouricemic volunteers by using both cell composition correction (as described in the main text of this manuscript) and no correction for cell composition. While this analysis identified 163 differentially methylated probes and 10 differentially methylated regions which were common for the targets identified by the two types of analyses (data not shown), new targets were also revealed. One example is *SOCS3* (suppressor of cytokine signaling 3), a regulator of STAT3 signaling with broad roles in cytokine signaling[18, 19], mostly associated with negative feedback inhibition of proinflammatory cytokines[20, 21]. Our data show higher DNA methylation in hyperuricemic individuals at 3 neighboring *SOCS3* intragenic regions (Fig.S2). Since the functionality of this higher methylation status is not known, future validation studies are necessary to show whether SOCS3 levels are indeed modified in the context of urate exposure *in vitro* or *in vivo*. If SOCS3 levels are diminished, this could typically coincide with less STAT3 inhibition and promotion of proinflammatory cytokines production[20, 21]. However, SOCS3 has been shown to have the dual role of upregulating TLR4 induced cytokines by TGFβ inhibition[22]. Therefore, the possible consequences of SOCS3 regulation in the context of urate priming are an interesting pathway to be tested and further validated in patients.

**REFERENCES**

1. Li Y, Oosting M, Deelen P, et al (2016) Inter-individual variability and genetic influences on cytokine responses to bacteria and fungi. Nat Med. https://doi.org/10.1038/nm.4139

2. Joosten LAB, Netea MG, Mylona E, et al (2010) Engagement of fatty acids with toll-like receptor 2 drives interleukin-1β production via the ASC/caspase 1 pathway in monosodium urate monohydrate crystal-induced gouty arthritis. Arthritis Rheum. https://doi.org/10.1002/art.27667

3. Repnik U, Knezevic M, Jeras M (2003) Simple and cost-effective isolation of monocytes from buffy coats. J Immunol Methods. https://doi.org/10.1016/S0022-1759(03)00231-X

4. Crişan TO, Cleophas MCP, Novakovic B, Erler K, Van De Veerdonk FL, Stunnenberg HG, Netea MG, Dinarello CA, Joosten LAB (2017) Uric acid priming in human monocytes is driven by the AKT-PRAS40 autophagy pathway. Proc Natl Acad Sci U S A. https://doi.org/10.1073/pnas.1620910114

5. Joosten LAB, Crisan TO, Azam T, Cleophas MCP, Koenders MI, Van De Veerdonk FL, Netea MG, Kim S, Dinarello CA (2016) Alpha-1-anti-trypsin-Fc fusion protein ameliorates gouty arthritis by reducing release and extracellular processing of IL-1β and by the induction of endogenous IL-1Ra. Ann Rheum Dis. https://doi.org/10.1136/annrheumdis-2014-206966

6. Novakovic B, Habibi E, Wang SY, et al (2016) β-Glucan Reverses the Epigenetic State of LPS-Induced Immunological Tolerance. Cell. https://doi.org/10.1016/j.cell.2016.09.034

7. Martino D, Neeland M, Dang T, Cobb J, Ellis J, Barnett A, Tang M, Vuillermin P, Allen K, Saffery R (2018) Epigenetic dysregulation of naive CD4+ T-cell activation genes in childhood food allergy. Nat Commun. https://doi.org/10.1038/s41467-018-05608-4

8. Pidsley R, Zotenko E, Peters TJ, Lawrence MG, Risbridger GP, Molloy P, Van Djik S, Muhlhausler B, Stirzaker C, Clark SJ (2016) Critical evaluation of the Illumina MethylationEPIC BeadChip microarray for whole-genome DNA methylation profiling. Genome Biol. https://doi.org/10.1186/s13059-016-1066-1

9. Phipson B, Maksimovic J, Oshlack A (2016) MissMethyl: An R package for analyzing data from Illumina’s HumanMethylation450 platform. Bioinformatics. https://doi.org/10.1093/bioinformatics/btv560

10. Aryee MJ, Jaffe AE, Corrada-Bravo H, Ladd-Acosta C, Feinberg AP, Hansen KD, Irizarry RA (2014) Minfi: A flexible and comprehensive Bioconductor package for the analysis of Infinium DNA methylation microarrays. Bioinformatics. https://doi.org/10.1093/bioinformatics/btu049

11. Gentleman RC, Carey VJ, Bates DM, et al (2004) Bioconductor: open software development for computational biology and bioinformatics. Genome Biol. https://doi.org/10.1186/gb-2004-5-10-r80

12. Maksimovic J, Gordon L, Oshlack A (2012) SWAN: Subset-quantile within array normalization for illumina infinium HumanMethylation450 BeadChips. Genome Biol. https://doi.org/10.1186/gb-2012-13-6-r44

13. Houseman EA, Molitor J, Marsit CJ (2014) Reference-free cell mixture adjustments in analysis of DNA methylation data. Bioinformatics. https://doi.org/10.1093/bioinformatics/btu029

14. Ritchie ME, Phipson B, Wu D, Hu Y, Law CW, Shi W, Smyth GK (2015) Limma powers differential expression analyses for RNA-sequencing and microarray studies. Nucleic Acids Res. https://doi.org/10.1093/nar/gkv007

15. Peters TJ, Buckley MJ, Statham AL, Pidsley R, Samaras K, V Lord R, Clark SJ, Molloy PL (2015) De novo identification of differentially methylated regions in the human genome. Epigenetics and Chromatin. https://doi.org/10.1186/1756-8935-8-6

16. Houseman EA, Accomando WP, Koestler DC, Christensen BC, Marsit CJ, Nelson HH, Wiencke JK, Kelsey KT (2012) DNA methylation arrays as surrogate measures of cell mixture distribution. BMC Bioinformatics. https://doi.org/10.1186/1471-2105-13-86

17. Barton SJ, Melton PE, Titcombe P, Murray R, Rauschert S, Lillycrop KA, Huang RC, Holbrook JD, Godfrey KM (2019) In Epigenomic Studies, Including Cell-Type Adjustments in Regression Models Can Introduce Multicollinearity, Resulting in Apparent Reversal of Direction of Association. Front Genet. https://doi.org/10.3389/fgene.2019.00816

18. Mahony R, Ahmed S, Diskin C, Stevenson NJ (2016) SOCS3 revisited: a broad regulator of disease, now ready for therapeutic use? Cell Mol Life Sci. https://doi.org/10.1007/s00018-016-2234-x

19. Yoshimura A, Suzuki M, Sakaguchi R, Hanada T, Yasukawa H (2012) SOCS, inflammation, and autoimmunity. Front Immunol 3:1–9

20. Yan C, Ward PA, Wang X, Gao H (2013) Myeloid depletion of SOCS3 enhances LPS-induced acute lung injury through CCAAT/enhancer binding protein δ pathway. FASEB J. https://doi.org/10.1096/fj.12-225797

21. Yoshimura A, Yasukawa H (2012) JAK’s SOCS: A Mechanism of Inhibition. Immunity. https://doi.org/10.1016/j.immuni.2012.01.010

22. Liu X, Zhang Y, Yu Y, Yang X, Cao X (2008) SOCS3 promotes TLR4 response in macrophages by feedback inhibiting TGF-β1/Smad3 signaling. Mol Immunol. https://doi.org/10.1016/j.molimm.2007.08.018

23. Ruppert J, Schtitt C, Ostermeier D, Peters JH CD14. 281–286

24. Kennedy MN, Mullen GED, Leifer CA, Lee CW, Mazzoni A, Dileepan KN, Segal DM (2004) A complex of soluble MD-2 and lipopolysaccharide serves as an activating ligand for toll-like receptor 4. J Biol Chem 279:34698–34704

**FIGURES AND TABLES**


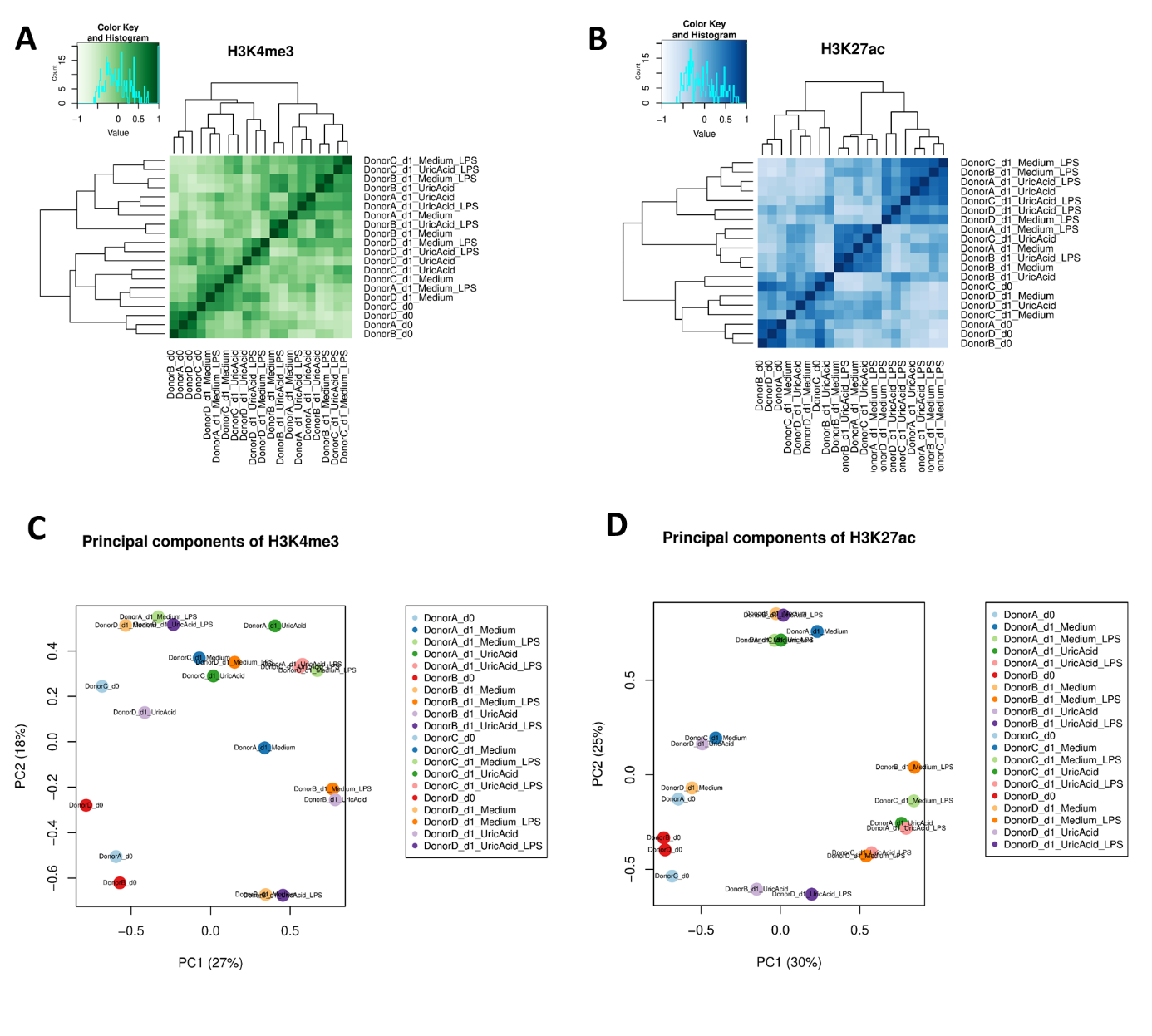


**Figure S1. ChIP-Seq analysis in freshly isolated monocytes at day 0 (d0), without culturing, reveals distinct clustering features compared to all stimulated samples obtained at day 1 (d1).** Cluster and principal component analysis of datasets obtained on ChIP sequencing for H3K4me3 (A and C) or H3K27ac (B and D) in 4 different donors (labeled D1-4) and 4 different conditions (Medium, Urate, Medium+LPS, Urate+LPS).

**
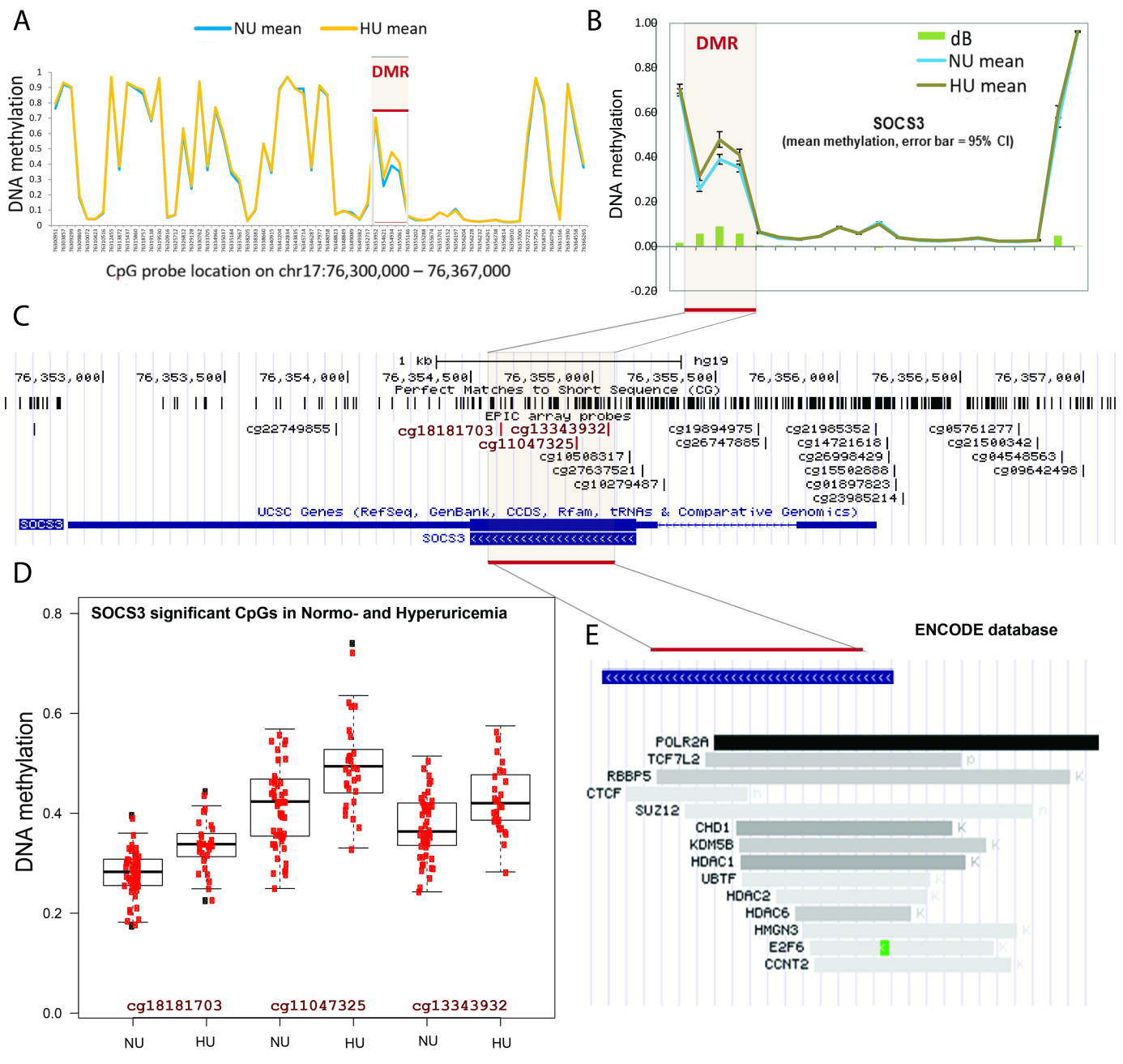
Figure S2.** **Differential DNA methylation analysis (without cell composition correction) in hyperuricemic versus normouricemic people.** DNA methylation levels (A) and absolute change inmean methylation between hyperuricemic (HU) and normouricemic (NU) people (B). UCSC genome browser snapshot of 3 intragenic probes located at the *SOCS3* locus (C). Individual methylation levels in NU compared o HU people for the 3 highlighted *SOCS3* probes (D). Transcription factors known to bind at the highlighted DMR region according to the transcription factor ChIP-seq clusters from ENCODE with factorbook motifs (E).

**Table S1. Patient characteristics for DNA methylation study**. NU=Normouricaemia, HU=Hyperuricaemia; Serum urate levels = mmol/L; NU <0.42mmol/L, HU>0.42mmol/L.

| **Group** | **Subjects** | **Age** | **Sex** | **BMI** | **Serum urate** | **Gout** |
| --- | --- | --- | --- | --- | --- | --- |
| **HU** | n = 26 | Min.   :20.00 | F = 7 | Min.   :30.40 | Min.   :0.4200 | n = 15 |
|  |  | 1st Qu.:41.00 | M = 19 | 1st Qu.:33.92 | 1st Qu.:0.4425 |  |
|  |  | Median :47.50 |  | Median :37.20 | Median :0.4800 |  |
|  |  | Mean   :47.88 |  | Mean   :39.74 | Mean   :0.4919 |  |
|  |  | 3rd Qu.:59.50 |  | 3rd Qu.:42.06 | 3rd Qu.:0.5200 |  |
|  |  | Max.   :70.00 |  | Max.   :66.90 | Max.   :0.6700 |  |
| **NU** | n = 50 | Min.   :25.00 | F = 33 | Min.   :27.01 | Min.   :0.0700 | n = 14 |
|  |  | 1st Qu.:42.25 | M = 17 | 1st Qu.:32.06 | 1st Qu.:0.2725 |  |
|  |  | Median :52.00 |  | Median :35.66 | Median :0.3300 |  |
|  |  | Mean   :51.52 |  | Mean   :36.74 | Mean   :0.3104 |  |
|  |  | 3rd Qu.:62.00 |  | 3rd Qu.:39.66 | 3rd Qu.:0.3800 |  |
|  |  | Max.   :83.00 |  | Max.   :56.25 | Max.   :0.4100 |  |

**Table S2.** Genes showing enrichment of histone modification H3K4me3.

| ENSEMBL gene ID | gene name | function | chr | Log 2 Fold change | p-val |
| --- | --- | --- | --- | --- | --- |
| ENSG00000008838 | MED24 | protein_coding | chr17 | 3,4531 | 0,0036 |
| ENSG00000108342 | CSF3 | protein_coding | chr17 | 3,4531 | 0,0036 |
| ENSG00000103168 | TAF1C | protein_coding | chr16 | 3,2611 | 0,0016 |
| ENSG00000154099 | DNAAF1 | protein_coding | chr16 | 3,2611 | 0,0016 |
| ENSG00000182782 | HCAR2 | protein_coding | chr12 | 2,7893 | 0,0035 |
| ENSG00000255398 | HCAR3 | protein_coding | chr12 | 2,4391 | 0,0051 |
| ENSG00000256249 | RP11-324E6.6 | lincRNA | chr12 | 2,4391 | 0,0051 |
| ENSG00000166278 | C2 | protein_coding | chr6 | 2,1957 | 0,0172 |
| ENSG00000179428 | AC073072.5 | antisense | chr7 | 1,8949 | 0,0109 |
| ENSG00000131203 | IDO1 | protein_coding | chr8 | 1,7988 | 0,0162 |
| ENSG00000253838 | RP11-44K6.2 | sense_intronic | chr8 | 1,7988 | 0,0162 |
| ENSG00000171855 | IFNB1 | protein_coding | chr9 | 1,7420 | 0,0444 |
| ENSG00000203364 | RP11-370F5.4 | lincRNA | chr9 | 1,7082 | 0,0317 |
| ENSG00000115009 | CCL20 | protein_coding | chr2 | 1,4683 | 0,0118 |
| ENSG00000229172 | AC073065.3 | pseudogene | chr2 | 1,4683 | 0,0118 |
| ENSG00000253320 | KB-1507C5.2 | protein_coding | chr8 | 1,4589 | 0,0305 |
| ENSG00000237568 | RP4-620F22.2 | antisense | chr1 | 1,3998 | 0,0438 |
| ENSG00000140379 | BCL2A1 | protein_coding | chr15 | 1,3622 | 0,0187 |
| ENSG00000253790 | RP11-44K6.5 | pseudogene | chr8 | 1,3460 | 0,0384 |
| ENSG00000095970 | TREM2 | protein_coding | chr6 | -1,4660 | 0,0192 |
| ENSG00000124562 | SNRPC | protein_coding | chr6 | -1,5600 | 0,0009 |
| ENSG00000130203 | APOE | protein_coding | chr19 | -1,6253 | 0,0146 |

**Table S3.** Genes showing enrichment of histone modification H3K27ac.

| ENSEMBL gene ID | gene name | function | chr | Log 2 Fold change | p-val |
| --- | --- | --- | --- | --- | --- |
| ENSG00000240481 | Metazoa_SRP | misc_RNA | chr9 | 2,6607 | 0,0494 |
| ENSG00000169184 | MN1 | protein_coding | chr22 | 2,4834 | 0,0083 |
| ENSG00000203364 | RP11-370F5.4 | lincRNA | chr9 | 2,3311 | 0,0298 |
| ENSG00000231525 | AC002486.2 | processed_pseudogene | chr7 | 2,3170 | 0,0237 |
| ENSG00000240624 | RP11-45P15.2 | processed_pseudogene | chr14 | 2,2890 | 0,0334 |
| ENSG00000234496 | MRPS21P1 | processed_pseudogene | chr1 | 2,2565 | 0,0380 |
| ENSG00000008838 | MED24 | protein_coding | chr17 | 2,2487 | 0,0314 |
| ENSG00000108342 | CSF3 | protein_coding | chr17 | 2,2487 | 0,0314 |
| ENSG00000238793 | SNORD124 | snoRNA | chr17 | 2,2487 | 0,0314 |
| ENSG00000131203 | IDO1 | protein_coding | chr8 | 2,1271 | 0,0298 |
| ENSG00000253838 | RP11-44K6.2 | sense_intronic | chr8 | 2,1271 | 0,0298 |
| ENSG00000101460 | MAP1LC3A | protein_coding | chr20 | 2,1127 | 0,0241 |
| ENSG00000101464 | PIGU | protein_coding | chr20 | 2,1127 | 0,0241 |
| ENSG00000134460 | IL2RA | protein_coding | chr10 | 2,0580 | 0,0362 |
| ENSG00000251922 | SNORA14 | snoRNA | chr10 | 2,0580 | 0,0362 |
| ENSG00000105855 | ITGB8 | retained_intron | chr7 | 2,0193 | 0,0351 |
| ENSG00000248719 | RP11-377G16.2 | antisense | chr4 | 2,0036 | 0,0298 |
| ENSG00000179428 | AC073072.5 | antisense | chr7 | 1,9881 | 0,0318 |
| ENSG00000188676 | IDO2 | processed_transcript | chr8 | 1,9871 | 0,0482 |
| ENSG00000253790 | RP11-44K6.5 | processed_pseudogene | chr8 | 1,9871 | 0,0482 |
| ENSG00000103168 | TAF1C | protein_coding | chr16 | 1,9753 | 0,0227 |
| ENSG00000154099 | DNAAF1 | retained_intron | chr16 | 1,9753 | 0,0227 |
| ENSG00000115008 | IL1A | protein_coding | chr2 | 1,9373 | 0,0193 |
| ENSG00000125538 | IL1B | protein_coding | chr2 | 1,9373 | 0,0193 |
| ENSG00000221541 | AC079753.1 | miRNA | chr2 | 1,9373 | 0,0193 |
| ENSG00000244104 | Metazoa_SRP | misc_RNA | chr12 | 1,8827 | 0,0098 |
| ENSG00000234869 | RP3-439F8.1 | antisense | chr22 | 1,8560 | 0,0305 |
| ENSG00000129277 | CCL4 | protein_coding | chr17 | 1,8478 | 0,0370 |
| ENSG00000263488 | Metazoa_SRP | misc_RNA | chr17 | 1,8478 | 0,0370 |
| ENSG00000264684 | MIR4773-2 | miRNA | chr2 | 1,8258 | 0,0384 |
| ENSG00000234956 | RP11-356I2.1 | lincRNA | chr6 | 1,8221 | 0,0219 |
| ENSG00000232591 | RP5-1031D4.2 | lincRNA | chr10 | 1,7670 | 0,0050 |
| ENSG00000250762 | RP1-313L4.4 | unprocessed_pseudogene | chr1 | 1,7670 | 0,0408 |
| ENSG00000259090 | RP11-173D9.5 | processed_pseudogene | chr14 | 1,7077 | 0,0378 |
| ENSG00000128271 | ADORA2A | processed_transcript | chr22 | 1,6242 | 0,0166 |
| ENSG00000178803 | ADORA2A-AS1 | protein_coding | chr22 | 1,6242 | 0,0166 |
| ENSG00000258555 | SPECC1L | nonsense_mediated_decay | chr22 | 1,6242 | 0,0166 |
| ENSG00000168260 | C14orf183 | protein_coding | chr14 | 1,5943 | 0,0289 |
| ENSG00000251792 | Y_RNA | misc_RNA | chr14 | 1,5943 | 0,0289 |
| ENSG00000110047 | EHD1 | protein_coding | chr11 | 1,5784 | 0,0121 |
| ENSG00000226169 | RP11-375H17.1 | antisense | chr22 | 1,5761 | 0,0165 |
| ENSG00000251230 | RP11-701P16.5 | processed_transcript | chr4 | 1,5183 | 0,0464 |
| ENSG00000266698 | MIR3945 | miRNA | chr4 | 1,5183 | 0,0464 |
| ENSG00000235842 | RP11-356I2.2 | processed_transcript | chr6 | 1,5052 | 0,0469 |
| ENSG00000160325 | CACFD1 | protein_coding | chr9 | 1,5017 | 0,0153 |
| ENSG00000160326 | SLC2A6 | protein_coding | chr9 | 1,5017 | 0,0153 |
| ENSG00000227898 | RP13-100B2.4 | antisense | chr9 | 1,5017 | 0,0153 |
| ENSG00000228923 | AP000355.2 | antisense | chr22 | 1,4950 | 0,0496 |
| ENSG00000254693 | RP11-58K22.5 | antisense | chr11 | 1,4674 | 0,0285 |
| ENSG00000227269 | RP11-96L7.2 | lincRNA | chr9 | 1,4561 | 0,0494 |
| ENSG00000240235 | Metazoa_SRP | misc_RNA | chr9 | 1,4561 | 0,0165 |
| ENSG00000268621 | AC006262.5 | lincRNA | chr19 | 1,4445 | 0,0308 |
| ENSG00000181577 | C6orf223 | retained_intron | chr6 | 1,4410 | 0,0011 |
| ENSG00000238486 | snoU13 | snoRNA | chr19 | 1,4326 | 0,0351 |
| ENSG00000173578 | XCR1 | protein_coding | chr3 | 1,4309 | 0,0429 |
| ENSG00000182782 | HCAR2 | protein_coding | chr12 | 1,4093 | 0,0343 |
| ENSG00000184588 | PDE4B | protein_coding | chr1 | 1,3800 | 0,0432 |
| ENSG00000169429 | IL8 | protein_coding | chr4 | 1,3583 | 0,0207 |
| ENSG00000228277 | AC112518.3 | lincRNA | chr4 | 1,3583 | 0,0207 |
| ENSG00000101337 | TM9SF4 | protein_coding | chr20 | 1,3472 | 0,0054 |
| ENSG00000232759 | AC002480.3 | processed_transcript | chr7 | -1,3221 | 0,0487 |
| ENSG00000223009 | AL138930.1 | miRNA | chr1 | -1,3501 | 0,0027 |
| ENSG00000235007 | RP11-344B5.4 | lincRNA | chr9 | -1,3508 | 0,0467 |
| ENSG00000124562 | SNRPC | protein_coding | chr6 | -1,3638 | 0,0133 |
| ENSG00000232131 | RP1-293L8.5 | antisense | chr6 | -1,4102 | 0,0082 |
| ENSG00000130203 | APOE | protein_coding | chr19 | -1,4381 | 0,0177 |
| ENSG00000267009 | RP11-120M18.2 | processed_transcript | chr17 | -1,4398 | 0,0217 |
| ENSG00000110079 | MS4A4A | protein_coding | chr11 | -1,4508 | 0,0047 |
| ENSG00000260872 | RP11-680G24.5 | antisense | chr16 | -1,4750 | 0,0065 |
| ENSG00000170323 | FABP4 | protein_coding | chr8 | -1,4973 | 0,0092 |
| ENSG00000141293 | SKAP1 | protein_coding | chr17 | -1,5263 | 0,0231 |
| ENSG00000269843 | CTC-490E21.10 | lincRNA | chr19 | -1,5742 | 0,0419 |
| ENSG00000197849 | OR8G7P | unprocessed_pseudogene | chr11 | -1,6011 | 0,0004 |
| ENSG00000255298 | OR8G1 | unprocessed_pseudogene | chr11 | -1,6011 | 0,0004 |
| ENSG00000204872 | AC092653.5 | unprocessed_pseudogene | chr2 | -1,6486 | 0,0430 |
| ENSG00000230490 | RP11-141M1.3 | lincRNA | chr13 | -1,6965 | 0,0169 |
| ENSG00000217746 | RP1-303A1.1 | processed_pseudogene | chr6 | -1,6984 | 0,0112 |
| ENSG00000235899 | RP11-345L23.1 | antisense | chr6 | -1,9337 | 0,0001 |

**Table S4.** Differentially methylated probes in whole blood of people with hyperuricemia compared to normouricemic people with cell composition correction

| Nr. | probe ID | nearest gene (distance in bp) | chr | NU_mean | HU_mean | dB | P.Value |
| --- | --- | --- | --- | --- | --- | --- | --- |
| 1 | cg20482334 | FASN (+7677) | chr17 | 0.715 | 0.858 | 0.143 | 0.0098 |
| 2 | cg21224286 | LRTM2 (-19517) | chr12 | 0.474 | 0.611 | 0.137 | 0.0016 |
| 3 | cg12859507 | LRTM2 (-19470) | chr12 | 0.289 | 0.415 | 0.126 | 0.0022 |
| 4 | cg21022775 | AREG (+28658) | chr4 | 0.685 | 0.810 | 0.126 | 0.0408 |
| 5 | cg20347269 | P2RX1 (+11637) | chr17 | 0.498 | 0.623 | 0.124 | 0.0003 |
| 6 | cg18850127 | POU6F2 (+152900) | chr7 | 0.484 | 0.608 | 0.124 | 0.0231 |
| 7 | cg24751894 | HLA-G (+99386) | chr6 | 0.144 | 0.266 | 0.123 | 0.0071 |
| 8 | cg18786623 | HLA-G (+99295) | chr6 | 0.239 | 0.361 | 0.121 | 0.0066 |
| 9 | cg15212455 | POU6F2 (+152942) | chr7 | 0.545 | 0.662 | 0.117 | 0.0153 |
| 10 | cg15070894 | HLA-G (+99407) | chr6 | 0.180 | 0.291 | 0.111 | 0.0072 |
| 11 | cg04520169 | HLA-G (+99440) | chr6 | 0.138 | 0.240 | 0.103 | 0.0300 |
| 12 | cg08951186 | ZNF469 (-203509) | chr16 | 0.531 | 0.631 | 0.101 | 0.0295 |
| 13 | cg26175789 | EVA1A (-25045) | chr2 | 0.385 | 0.485 | 0.100 | 0.0173 |
| 14 | cg07973162 | UGT2B17 (-1006) | chr4 | 0.409 | 0.504 | 0.095 | 0.0179 |
| 15 | cg08231349 | HLA-G (+99889) | chr6 | 0.051 | 0.146 | 0.095 | 0.0043 |
| 16 | cg03806328 | ARHGAP32 (-67539) | chr11 | 0.588 | 0.683 | 0.095 | 0.0226 |
| 17 | cg13365324 | UGT2B17 (-1229) | chr4 | 0.427 | 0.521 | 0.094 | 0.0163 |
| 18 | cg07952421 | UGT2B17 (-1357) | chr4 | 0.525 | 0.618 | 0.092 | 0.0152 |
| 19 | cg10388667 | MRPL53 (+2528) | chr2 | 0.687 | 0.779 | 0.092 | 0.0049 |
| 20 | cg24239165 | C17orf82 (+7542) | chr17 | 0.580 | 0.672 | 0.091 | 0.0001 |
| 21 | cg20302533 | POU6F2 (+153166) | chr7 | 0.266 | 0.355 | 0.089 | 0.0375 |
| 22 | cg22772380 | ZNF12 (-484) | chr7 | 0.445 | 0.533 | 0.088 | 0.0321 |
| 23 | cg14381313 | ZNF469 (-225540) | chr16 | 0.493 | 0.579 | 0.085 | 0.0364 |
| 24 | cg26353469 | HLA-G (+61215) | chr6 | 0.085 | 0.170 | 0.085 | 0.0404 |
| 25 | cg14904295 | PEG3 (+3535) | chr19 | 0.291 | 0.375 | 0.084 | 0.0262 |
| 26 | cg03660162 | MAF (+721526) | chr16 | 0.710 | 0.793 | 0.083 | 0.0229 |
| 27 | cg21332948 | AGPAT3 (-21173) | chr21 | 0.674 | 0.758 | 0.083 | 0.0428 |
| 28 | cg07346359 | OPTC (+26398) | chr1 | 0.422 | 0.505 | 0.083 | 0.0122 |
| 29 | cg03995122 | HLA-G (+99887) | chr6 | 0.030 | 0.113 | 0.083 | 0.0183 |
| 30 | cg21549632 | HLA-G (+61532) | chr6 | 0.066 | 0.148 | 0.082 | 0.0128 |
| 31 | cg15671450 | HLA-G (+100361) | chr6 | 0.089 | 0.168 | 0.079 | 0.0497 |
| 32 | cg16302021 | HLA-G (+99189) | chr6 | 0.404 | 0.481 | 0.078 | 0.0230 |
| 33 | cg00409917 | HLA-G (+99924) | chr6 | 0.074 | 0.152 | 0.077 | 0.0068 |
| 34 | cg00901687 | MYCBPAP (-475) | chr17 | 0.453 | 0.529 | 0.076 | 0.0250 |
| 35 | cg23237314 | HLA-G (+99442) | chr6 | 0.130 | 0.206 | 0.076 | 0.0401 |
| 36 | cg19823512 | OPTC (+26455) | chr1 | 0.305 | 0.380 | 0.075 | 0.0388 |
| 37 | cg09597767 | IFITM3 (-4866) | chr11 | 0.700 | 0.772 | 0.072 | 0.0036 |
| 38 | cg21323039 | MATN1 (+3484) | chr1 | 0.792 | 0.863 | 0.072 | 0.0063 |
| 39 | cg01336390 | HLA-G (+100304) | chr6 | 0.087 | 0.159 | 0.072 | 0.0118 |
| 40 | cg26028489 | LMO3 (+85999) | chr12 | 0.478 | 0.550 | 0.072 | 0.0297 |
| 41 | cg17341345 | CCNH (-574) | chr5 | 0.505 | 0.575 | 0.071 | 0.0151 |
| 42 | cg26054541 | GSDMC (+815502) | chr8 | 0.691 | 0.761 | 0.070 | 0.0034 |
| 43 | cg22968327 | NUP93 (+59405) | chr16 | 0.258 | 0.327 | 0.069 | 0.0406 |
| 44 | cg14449180 | HLA-G (+99864) | chr6 | 0.040 | 0.109 | 0.069 | 0.0293 |
| 45 | cg10632656 | UGT2B17 (-1349) | chr4 | 0.274 | 0.343 | 0.069 | 0.0171 |
| 46 | cg06223984 | UGT2B17 (-7507) | chr4 | 0.561 | 0.629 | 0.068 | 0.0230 |
| 47 | cg13597893 | UBE2E3 (-272789) | chr2 | 0.493 | 0.561 | 0.068 | 0.0121 |
| 48 | cg03071500 | IFITM3 (-4915) | chr11 | 0.562 | 0.629 | 0.067 | 0.0012 |
| 49 | cg10193107 | HSPB1 (-7591) | chr7 | 0.481 | 0.547 | 0.067 | 0.0378 |
| 50 | cg00738934 | PKIA (-432095) | chr8 | 0.487 | 0.554 | 0.067 | 0.0010 |
| 51 | cg21665744 | POU6F2 (+153516) | chr7 | 0.631 | 0.697 | 0.066 | 0.0211 |
| 52 | cg15931205 | HLA-G (+100065) | chr6 | 0.060 | 0.126 | 0.066 | 0.0121 |
| 53 | cg18191116 | STON2 (+241) | chr14 | 0.502 | 0.568 | 0.065 | 0.0475 |
| 54 | cg09549987 | SPAG11B (+644) | chr8 | 0.310 | 0.374 | 0.064 | 0.0091 |
| 55 | cg22253032 | PRSS1 (-37378) | chr7 | 0.431 | 0.495 | 0.063 | 0.0012 |
| 56 | cg21862992 | MRPL21 (+12905) | chr11 | 0.404 | 0.467 | 0.063 | 0.0418 |
| 57 | cg15598217 | ZZEF1 (+44435) | chr17 | 0.524 | 0.587 | 0.063 | 0.0237 |
| 58 | cg00135841 | USP18 (+104683) | chr22 | 0.762 | 0.825 | 0.063 | 0.0423 |
| 59 | cg11440486 | MYCBPAP (-529) | chr17 | 0.462 | 0.524 | 0.062 | 0.0347 |
| 60 | cg00186468 | CCNH (-393) | chr5 | 0.248 | 0.310 | 0.062 | 0.0095 |
| 61 | cg11449146 | GAS2 (+62304) | chr11 | 0.555 | 0.617 | 0.062 | 0.0034 |
| 62 | cg20111217 | MYCBPAP (-481) | chr17 | 0.542 | 0.604 | 0.062 | 0.0171 |
| 63 | cg27584762 | CCNH (-291) | chr5 | 0.207 | 0.269 | 0.062 | 0.0202 |
| 64 | cg19748509 | HLA-G (+116023) | chr6 | 0.070 | 0.131 | 0.061 | 0.0152 |
| 65 | cg01617603 | UGT2B17 (+15538) | chr4 | 0.243 | 0.304 | 0.061 | 0.0116 |
| 66 | cg03249561 | FCAR (+14427) | chr19 | 0.674 | 0.734 | 0.060 | 0.0275 |
| 67 | cg04815973 | STOML1 (+17521) | chr15 | 0.251 | 0.311 | 0.060 | 0.0453 |
| 68 | cg06112835 | MRPL21 (+12495) | chr11 | 0.293 | 0.352 | 0.059 | 0.0399 |
| 69 | cg19481811 | UGT2B17 (+563) | chr4 | 0.587 | 0.646 | 0.059 | 0.0314 |
| 70 | cg15218522 | SBSPON (-75679) | chr8 | 0.333 | 0.392 | 0.059 | 0.0225 |
| 71 | cg21894124 | FBRSL1 (-161448) | chr12 | 0.146 | 0.205 | 0.059 | 0.0018 |
| 72 | cg11994053 | VEZT (+100370) | chr12 | 0.740 | 0.799 | 0.059 | 0.0034 |
| 73 | cg04184807 | GAPVD1 (+207038) | chr9 | 0.672 | 0.731 | 0.059 | 0.0386 |
| 74 | cg17453273 | ITSN2 (-61147) | chr2 | 0.116 | 0.174 | 0.059 | 0.0135 |
| 75 | cg09274344 | SOX8 (+46882) | chr16 | 0.313 | 0.372 | 0.059 | 0.0211 |
| 76 | cg01062395 | HLA-G (+60881) | chr6 | 0.110 | 0.169 | 0.058 | 0.0212 |
| 77 | cg09874992 | SDC3 (+67000) | chr1 | 0.372 | 0.430 | 0.057 | 0.0026 |
| 78 | cg08743428 | ROBO2 (+678273) | chr3 | 0.456 | 0.513 | 0.057 | 0.0070 |
| 79 | cg18976649 | LMX1A (+213468) | chr1 | 0.718 | 0.774 | 0.056 | 0.0011 |
| 80 | cg14453346 | TSPAN33 (+19652) | chr7 | 0.807 | 0.862 | 0.056 | 0.0389 |
| 81 | cg21733854 | ZSCAN22 (-615) | chr19 | 0.517 | 0.571 | 0.055 | 0.0387 |
| 82 | cg19727165 | DHFRL1 (-114061) | chr3 | 0.608 | 0.663 | 0.055 | 0.0077 |
| 83 | cg07398106 | LDHC (+318) | chr11 | 0.450 | 0.505 | 0.054 | 0.0182 |
| 84 | cg20476087 | CGREF1 (-126) | chr2 | 0.117 | 0.171 | 0.054 | 0.0018 |
| 85 | cg21705926 | DNAJB6 (+276978) | chr7 | 0.483 | 0.537 | 0.054 | 0.0385 |
| 86 | cg14080482 | NONE | chr6 | 0.383 | 0.438 | 0.054 | 0.0182 |
| 87 | cg10773881 | GGA1 (+15331) | chr22 | 0.115 | 0.170 | 0.054 | 0.0314 |
| 88 | cg26942295 | PDZD2 (+49191) | chr5 | 0.494 | 0.548 | 0.054 | 0.0249 |
| 89 | cg13719529 | C11orf44 (+4653) | chr11 | 0.513 | 0.567 | 0.054 | 0.0379 |
| 90 | cg19445457 | OR52N5 (+451) | chr11 | 0.531 | 0.584 | 0.054 | 0.0197 |
| 91 | cg09142843 | ARHGAP18 (+389699) | chr6 | 0.737 | 0.790 | 0.053 | 0.0264 |
| 92 | cg13910785 | HLA-DRB1 (+7776) | chr6 | 0.283 | 0.336 | 0.053 | 0.0040 |
| 93 | cg23917496 | UACA (+300798) | chr15 | 0.379 | 0.432 | 0.053 | 0.0416 |
| 94 | cg15853715 | C14orf39 (-2535) | chr14 | 0.447 | 0.500 | 0.053 | 0.0059 |
| 95 | cg12884719 | SIPA1L2 (+451040) | chr1 | 0.420 | 0.473 | 0.053 | 0.0053 |
| 96 | cg22083892 | KCNJ8 (-907) | chr12 | 0.341 | 0.394 | 0.053 | 0.0215 |
| 97 | cg26637898 | PAPD7 (+44310) | chr5 | 0.804 | 0.857 | 0.053 | 0.0396 |
| 98 | cg14332815 | LDHC (-290) | chr11 | 0.403 | 0.456 | 0.053 | 0.0267 |
| 99 | cg13056653 | UCP1 (-49011) | chr4 | 0.519 | 0.572 | 0.053 | 0.0220 |
| 100 | cg13048967 | CXCR1 (+1595) | chr2 | 0.624 | 0.676 | 0.052 | 0.0150 |
| 101 | cg03278639 | DNAJB8 (+50966) | chr3 | 0.672 | 0.724 | 0.052 | 0.0003 |
| 102 | cg23999422 | SIRT6 (+9135) | chr19 | 0.228 | 0.280 | 0.052 | 0.0024 |
| 103 | cg03518414 | TOPAZ1 (-104724) | chr3 | 0.424 | 0.476 | 0.052 | 0.0270 |
| 104 | cg07040013 | GLRX3 (+164891) | chr10 | 0.351 | 0.402 | 0.052 | 0.0056 |
| 105 | cg06012695 | TRIM27 (+121173) | chr6 | 0.296 | 0.347 | 0.051 | 0.0015 |
| 106 | cg15236555 | HS1BP3 (+7072) | chr2 | 0.319 | 0.370 | 0.051 | 0.0178 |
| 107 | cg23155965 | TTC40 (-22088) | chr10 | 0.788 | 0.839 | 0.051 | 0.0060 |
| 108 | cg01521378 | ZNRD1 (-40965) | chr6 | 0.713 | 0.764 | 0.051 | 0.0288 |
| 109 | cg06902219 | HLA-G (+61539) | chr6 | 0.102 | 0.152 | 0.051 | 0.0144 |
| 110 | cg15279541 | PDCD2 (-142631) | chr6 | 0.426 | 0.477 | 0.051 | 0.0297 |
| 111 | cg24931191 | STAC (-172977) | chr3 | 0.264 | 0.315 | 0.051 | 0.0063 |
| 112 | cg03742947 | HLA-G (+61520) | chr6 | 0.067 | 0.118 | 0.051 | 0.0407 |
| 113 | cg21234506 | BCL2A1 (+656) | chr15 | 0.388 | 0.438 | 0.051 | 0.0477 |
| 114 | cg19107429 | FAM86B2 (+21475) | chr8 | 0.646 | 0.696 | 0.050 | 0.0196 |
| 115 | cg05707985 | PRSS1 (-26980) | chr7 | 0.294 | 0.344 | 0.050 | 0.0093 |
| 116 | cg11395062 | HS3ST3B1 (-64543) | chr17 | 0.710 | 0.760 | 0.050 | 0.0388 |
| 117 | cg13503915 | SIGLEC5 (-640) | chr19 | 0.638 | 0.588 | -0.050 | 0.0199 |
| 118 | cg21158434 | CLSTN3 (+8365) | chr12 | 0.700 | 0.650 | -0.050 | 0.0359 |
| 119 | cg12709009 | DLL1 (+41979) | chr6 | 0.622 | 0.571 | -0.050 | 0.0028 |
| 120 | cg18838701 | TNNI3 (+488) | chr19 | 0.309 | 0.259 | -0.050 | 0.0094 |
| 121 | cg17707095 | MPC1 (-197056) | chr6 | 0.653 | 0.602 | -0.051 | 0.0494 |
| 122 | cg14222229 | HIST2H3PS2 (+262795) | chr1 | 0.326 | 0.275 | -0.051 | 0.0111 |
| 123 | cg15797131 | IRS2 (+54603) | chr13 | 0.547 | 0.496 | -0.051 | 0.0403 |
| 124 | cg11524065 | ZCCHC8 (+21993) | chr12 | 0.832 | 0.781 | -0.051 | 0.0084 |
| 125 | cg10218876 | TNNT1 (+97) | chr19 | 0.245 | 0.193 | -0.051 | 0.0007 |
| 126 | cg04737881 | TENM3 (-103639) | chr4 | 0.326 | 0.275 | -0.051 | 0.0295 |
| 127 | cg00143193 | SMYD3 (+519171) | chr1 | 0.753 | 0.702 | -0.051 | 0.0490 |
| 128 | cg04161902 | BAI2 (+1728) | chr1 | 0.732 | 0.681 | -0.051 | 0.0004 |
| 129 | cg12463722 | OR4D1 (+6) | chr17 | 0.730 | 0.678 | -0.052 | 0.0225 |
| 130 | cg24045276 | NCF2 (+7621) | chr1 | 0.654 | 0.603 | -0.052 | 0.0133 |
| 131 | cg20212775 | SPON2 (+1980) | chr4 | 0.639 | 0.587 | -0.052 | 0.0057 |
| 132 | cg26965779 | HLA-G (+59334) | chr6 | 0.507 | 0.455 | -0.052 | 0.0088 |
| 133 | cg12847536 | ARHGEF10 (+50045) | chr8 | 0.636 | 0.584 | -0.052 | 0.0441 |
| 134 | cg22027267 | COBL (-160023) | chr7 | 0.751 | 0.699 | -0.052 | 0.0094 |
| 135 | cg22898160 | NDRG4 (-1339) | chr16 | 0.662 | 0.609 | -0.053 | 0.0207 |
| 136 | cg08121925 | FXR1 (-42049) | chr3 | 0.324 | 0.271 | -0.053 | 0.0020 |
| 137 | cg03321588 | FAM208A (-70669) | chr3 | 0.462 | 0.409 | -0.053 | 0.0398 |
| 138 | cg20660297 | KRR1 (-903) | chr12 | 0.532 | 0.479 | -0.053 | 0.0054 |
| 139 | cg07510085 | IL15RA (+18184) | chr10 | 0.721 | 0.667 | -0.054 | 0.0079 |
| 140 | cg05010260 | NAT16 (+3266) | chr7 | 0.610 | 0.556 | -0.054 | 0.0070 |
| 141 | cg15324331 | SVIL (-60404) | chr10 | 0.652 | 0.598 | -0.054 | 0.0054 |
| 142 | cg20304530 | NTRK3 (+297163) | chr15 | 0.798 | 0.744 | -0.054 | 0.0158 |
| 143 | cg01540595 | LHX8 (-3159) | chr1 | 0.468 | 0.413 | -0.054 | 0.0113 |
| 144 | cg01799458 | HOXB13 (+1736) | chr17 | 0.569 | 0.514 | -0.055 | 0.0353 |
| 145 | cg00590063 | B3GNT3 (+12877) | chr19 | 0.336 | 0.281 | -0.055 | 0.0457 |
| 146 | cg11016221 | PTGES (+9679) | chr9 | 0.411 | 0.356 | -0.055 | 0.0416 |
| 147 | cg08071329 | DLL1 (+43704) | chr6 | 0.739 | 0.683 | -0.056 | 0.0096 |
| 148 | cg19504245 | TNNT1 (+102) | chr19 | 0.294 | 0.238 | -0.056 | 0.0031 |
| 149 | cg09810149 | NFU1 (-27150) | chr2 | 0.655 | 0.599 | -0.056 | 0.0295 |
| 150 | cg20336352 | HERC4 (-13229) | chr10 | 0.757 | 0.701 | -0.056 | 0.0032 |
| 151 | cg16603012 | APRT (-1242) | chr16 | 0.660 | 0.604 | -0.057 | 0.0037 |
| 152 | cg19902553 | JPH3 (+49910) | chr16 | 0.241 | 0.184 | -0.057 | 0.0394 |
| 153 | cg08124399 | DDX43 (+398) | chr6 | 0.730 | 0.672 | -0.057 | 0.0469 |
| 154 | cg27516945 | ZNF528 (-603) | chr19 | 0.669 | 0.611 | -0.057 | 0.0010 |
| 155 | cg08122831 | ERBB4 (-294015) | chr2 | 0.767 | 0.710 | -0.057 | 0.0356 |
| 156 | cg06968712 | MS4A15 (+10431) | chr11 | 0.553 | 0.495 | -0.058 | 0.0024 |
| 157 | cg18423549 | MTRNR2L1 (-278559) | chr17 | 0.394 | 0.336 | -0.058 | 0.0050 |
| 158 | cg09602542 | GGT6 (-36137) | chr17 | 0.840 | 0.782 | -0.058 | 0.0176 |
| 159 | cg15199034 | ADAMTS1 (-42681) | chr21 | 0.701 | 0.642 | -0.058 | 0.0003 |
| 160 | cg17240725 | ABHD11 (+3841) | chr7 | 0.393 | 0.335 | -0.058 | 0.0207 |
| 161 | cg25122629 | EML6 (-14857) | chr2 | 0.360 | 0.301 | -0.059 | 0.0009 |
| 162 | cg05176970 | GLOD4 (-38693) | chr17 | 0.654 | 0.593 | -0.060 | 0.0009 |
| 163 | cg24441899 | FOXK1 (-477568) | chr7 | 0.561 | 0.501 | -0.060 | 0.0112 |
| 164 | cg18503693 | FAM155A (+595468) | chr13 | 0.618 | 0.557 | -0.061 | 0.0356 |
| 165 | cg15925527 | WRAP73 (+6530) | chr1 | 0.733 | 0.672 | -0.062 | 0.0462 |
| 166 | cg10491108 | TST (+6245) | chr22 | 0.640 | 0.578 | -0.062 | 0.0352 |
| 167 | cg22647161 | TMPO (-223043) | chr12 | 0.591 | 0.529 | -0.062 | 0.0407 |
| 168 | cg10432947 | NAT8L (+1203) | chr4 | 0.578 | 0.516 | -0.062 | 0.0000 |
| 169 | cg09430341 | CCDC17 (-481) | chr1 | 0.762 | 0.700 | -0.062 | 0.0129 |
| 170 | cg22871949 | NOM1 (-6814) | chr7 | 0.639 | 0.577 | -0.062 | 0.0119 |
| 171 | cg08617160 | MIER2 (-520) | chr19 | 0.542 | 0.479 | -0.062 | 0.0042 |
| 172 | cg07442889 | SIGLEC5 (-914) | chr19 | 0.694 | 0.631 | -0.063 | 0.0029 |
| 173 | cg21158163 | CDH4 (-284904) | chr20 | 0.809 | 0.746 | -0.063 | 0.0057 |
| 174 | cg17014757 | CHI3L1 (-221) | chr1 | 0.393 | 0.330 | -0.064 | 0.0183 |
| 175 | cg22526555 | KCNJ15 (-49635) | chr21 | 0.771 | 0.706 | -0.065 | 0.0002 |
| 176 | cg19708055 | C6orf123 (+151983) | chr6 | 0.647 | 0.581 | -0.065 | 0.0174 |
| 177 | cg16814483 | PRKAB2 (+94049) | chr1 | 0.500 | 0.435 | -0.066 | 0.0400 |
| 178 | cg10852875 | IRX2 (+214079) | chr5 | 0.589 | 0.523 | -0.066 | 0.0000 |
| 179 | cg10292709 | MCPH1 (-435562) | chr8 | 0.715 | 0.649 | -0.066 | 0.0065 |
| 180 | cg14248883 | SERP2 (+30700) | chr13 | 0.736 | 0.670 | -0.066 | 0.0095 |
| 181 | cg12159505 | DDX56 (+8747) | chr7 | 0.770 | 0.704 | -0.066 | 0.0021 |
| 182 | cg17959580 | ELFN2 (-1113) | chr22 | 0.648 | 0.581 | -0.067 | 0.0419 |
| 183 | cg23441030 | CACYBP (-59405) | chr1 | 0.712 | 0.645 | -0.067 | 0.0089 |
| 184 | cg15634083 | HLA-B (+86056) | chr6 | 0.204 | 0.137 | -0.067 | 0.0107 |
| 185 | cg09189601 | UGT2B15 (+22315) | chr4 | 0.480 | 0.410 | -0.070 | 0.0264 |
| 186 | cg20478239 | COBL (-159980) | chr7 | 0.691 | 0.620 | -0.071 | 0.0010 |
| 187 | cg16754099 | THEM6 (+1615) | chr8 | 0.560 | 0.488 | -0.072 | 0.0005 |
| 188 | cg05056638 | NEFM (+30300) | chr8 | 0.598 | 0.525 | -0.073 | 0.0411 |
| 189 | cg07569483 | NLRP9 (-8089) | chr19 | 0.634 | 0.558 | -0.076 | 0.0014 |
| 190 | cg01880147 | FCGR3B (-8389) | chr1 | 0.702 | 0.625 | -0.077 | 0.0010 |
| 191 | cg00256329 | GLOD4 (-38794) | chr17 | 0.692 | 0.614 | -0.078 | 0.0014 |
| 192 | cg24480926 | SFSWAP (-276103) | chr12 | 0.629 | 0.550 | -0.079 | 0.0430 |
| 193 | cg18220841 | BANP (+156623) | chr16 | 0.551 | 0.472 | -0.080 | 0.0446 |
| 194 | cg05758861 | BAI1 (+50033) | chr8 | 0.756 | 0.675 | -0.081 | 0.0117 |
| 195 | cg22445217 | QKI (+585402) | chr6 | 0.690 | 0.609 | -0.081 | 0.0020 |
| 196 | cg18325044 | HLA-G (+74145) | chr6 | 0.812 | 0.730 | -0.081 | 0.0086 |
| 197 | cg09105440 | THEM6 (+1617) | chr8 | 0.549 | 0.468 | -0.082 | 0.0015 |
| 198 | cg13784004 | MYO5B (+24730) | chr18 | 0.564 | 0.482 | -0.082 | 0.0069 |
| 199 | cg03526459 | PRKAB2 (+94183) | chr1 | 0.630 | 0.546 | -0.084 | 0.0482 |
| 200 | cg06451157 | HLA-G (+73540) | chr6 | 0.792 | 0.706 | -0.085 | 0.0169 |
| 201 | cg10805896 | PLXNA4 (-88990) | chr7 | 0.439 | 0.350 | -0.089 | 0.0411 |
| 202 | cg19729930 | BOLA3 (+17249) | chr2 | 0.596 | 0.506 | -0.090 | 0.0119 |
| 203 | cg00639946 | NONE | chr19 | 0.499 | 0.408 | -0.091 | 0.0215 |
| 204 | cg10804687 | HLA-G (+64765) | chr6 | 0.858 | 0.765 | -0.093 | 0.0458 |
| 205 | cg09163930 | PDGFRB (+1459) | chr5 | 0.505 | 0.409 | -0.096 | 0.0166 |
| 206 | cg10474018 | HLA-G (+65261) | chr6 | 0.871 | 0.774 | -0.097 | 0.0043 |
| 207 | cg01017244 | BOLA3 (+17594) | chr2 | 0.717 | 0.620 | -0.097 | 0.0097 |
| 208 | cg26127187 | HLA-G (+62040) | chr6 | 0.807 | 0.708 | -0.099 | 0.0158 |
| 209 | cg20891558 | BOLA3 (+17270) | chr2 | 0.565 | 0.466 | -0.099 | 0.0048 |
| 210 | cg15514307 | PPP2CA (-20989) | chr5 | 0.743 | 0.644 | -0.099 | 0.0120 |
| 211 | cg14018363 | HLA-G (+116510) | chr6 | 0.504 | 0.404 | -0.100 | 0.0137 |
| 212 | cg06454464 | TSNARE1 (+56622) | chr8 | 0.833 | 0.732 | -0.101 | 0.0139 |
| 213 | cg19077165 | TCEB3CL2 (-2555) | chr18 | 0.744 | 0.637 | -0.106 | 0.0054 |
| 214 | cg26649688 | HLA-G (+63605) | chr6 | 0.882 | 0.774 | -0.108 | 0.0088 |
| 215 | cg18423635 | HLA-G (+75181) | chr6 | 0.853 | 0.744 | -0.109 | 0.0345 |
| 216 | cg24179288 | HLA-G (+72530) | chr6 | 0.860 | 0.745 | -0.115 | 0.0140 |
| 217 | cg03395495 | GOLGA6L4 (-83129) | chr15 | 0.706 | 0.582 | -0.124 | 0.0495 |
| 218 | cg15825968 | SFSWAP (-276155) | chr12 | 0.614 | 0.489 | -0.125 | 0.0011 |
| 219 | cg20381372 | ZFP14 (+68693) | chr19 | 0.571 | 0.445 | -0.126 | 0.0116 |
| 220 | cg12046183 | HLA-G (+65074) | chr6 | 0.723 | 0.594 | -0.129 | 0.0033 |
| 221 | cg25817503 | AFAP1 (+153311) | chr4 | 0.596 | 0.463 | -0.133 | 0.0030 |
| 222 | cg05890377 | BOLA3 (+17408) | chr2 | 0.604 | 0.465 | -0.139 | 0.0167 |
| 223 | cg13393919 | GPBAR1 (-10899) | chr2 | 0.788 | 0.602 | -0.187 | 0.0009 |

**Table S5.** Differentially methylated regions in whole blood of people with hyperuricemia compared to normouricemic people with cell composition correction

| Nr. | nearest gene | DMR chr | DMR start | DMR end | length | probes in dmr |
| --- | --- | --- | --- | --- | --- | --- |
| 1 | HLA-G | chr6 | 29893273 | 29895204 | 1932 | 33 |
| 2 | HLA-G | chr6 | 29855890 | 29856564 | 675 | 19 |
| 3 | LDHC | chr11 | 18433500 | 18434354 | 855 | 10 |
| 4 | DOC2A | chr16 | 30023028 | 30024074 | 1047 | 10 |
| 5 | TNNT1 | chr19 | 55660514 | 55661528 | 1015 | 10 |
| 6 | DLL1 | chr6 | 170553133 | 170555857 | 2725 | 10 |
| 7 | IRX2 | chr5 | 2537210 | 2537834 | 625 | 8 |
| 8 | TNNI3 | chr19 | 55667533 | 55669059 | 1527 | 6 |
| 9 | FXR1 | chr3 | 180587900 | 180588228 | 329 | 6 |
| 10 | NAT8L | chr4 | 2061923 | 2063566 | 1644 | 6 |
| 11 | C6orf123 | chr6 | 168045268 | 168046457 | 1190 | 6 |
| 12 | MYCBPAP | chr17 | 48585216 | 48585470 | 255 | 5 |
| 13 | BOLA3 | chr2 | 74357527 | 74358223 | 697 | 5 |
| 14 | DLL1 | chr6 | 170557102 | 170558102 | 1001 | 5 |
| 15 | NOM1 | chr7 | 156735260 | 156735656 | 397 | 5 |
| 16 | BAI1 | chr8 | 143580770 | 143581481 | 712 | 5 |
| 17 | UGT2B17 | chr4 | 69435250 | 69435601 | 352 | 4 |
| 18 | THEM6 | chr8 | 143809371 | 143810237 | 867 | 4 |
| 19 | FBRSL1 | chr12 | 132904540 | 132904796 | 257 | 3 |
| 20 | ERBB4 | chr2 | 213697579 | 213698158 | 580 | 3 |
| 21 | KCNJ15 | chr21 | 39578742 | 39579277 | 536 | 3 |
| 22 | HLA-G | chr6 | 29859520 | 29860016 | 497 | 3 |
| 23 | POU6F2 | chr7 | 39170497 | 39170763 | 267 | 3 |

**Table S6.** Differentially methylated probes in whole blood of people with hyperuricemia compared to normouricemic people without cell composition correction

| Nr. | probe ID | nearest gene (distance in bp) | chr | NU_mean | HU_mean | dB | P.Value |
| --- | --- | --- | --- | --- | --- | --- | --- |
| 1 | cg07334023 | IL2RB (-9994) | chr22 | 0.487 | 0.668 | 0.182 | 0.0090 |
| 2 | cg02821156 | ANKH (+54827) | chr5 | 0.566 | 0.738 | 0.173 | 0.0045 |
| 3 | cg20482334 | FASN (+7677) | chr17 | 0.713 | 0.857 | 0.144 | 0.0011 |
| 4 | cg21224286 | LRTM2 (-19517) | chr12 | 0.476 | 0.613 | 0.137 | 0.0003 |
| 5 | cg13632935 | RPL12 (+6177) | chr9 | 0.495 | 0.624 | 0.129 | 0.0413 |
| 6 | cg07412545 | GPR88 (+232) | chr1 | 0.472 | 0.599 | 0.127 | 0.0256 |
| 7 | cg12859507 | LRTM2 (-19470) | chr12 | 0.293 | 0.420 | 0.127 | 0.0000 |
| 8 | cg18850127 | POU6F2 (+152900) | chr7 | 0.479 | 0.604 | 0.125 | 0.0003 |
| 9 | cg21022775 | AREG (+28658) | chr4 | 0.688 | 0.813 | 0.124 | 0.0035 |
| 10 | cg20347269 | P2RX1 (+11637) | chr17 | 0.501 | 0.625 | 0.124 | 0.0127 |
| 11 | cg24751894 | HLA-G (+99386) | chr6 | 0.146 | 0.269 | 0.123 | 0.0037 |
| 12 | cg26294610 | MUT (+158454) | chr6 | 0.525 | 0.647 | 0.122 | 0.0287 |
| 13 | cg18786623 | HLA-G (+99295) | chr6 | 0.243 | 0.363 | 0.121 | 0.0025 |
| 14 | cg16515087 | EFCAB6 (-22809) | chr22 | 0.315 | 0.435 | 0.119 | 0.0000 |
| 15 | cg12432526 | TMEM105 (-50229) | chr17 | 0.316 | 0.432 | 0.117 | 0.0149 |
| 16 | cg15212455 | POU6F2 (+152942) | chr7 | 0.551 | 0.666 | 0.116 | 0.0010 |
| 17 | cg06405219 | MTURN (+44710) | chr7 | 0.267 | 0.381 | 0.114 | 0.0446 |
| 18 | cg15070894 | HLA-G (+99407) | chr6 | 0.183 | 0.294 | 0.111 | 0.0084 |
| 19 | cg06223162 | GPR88 (-5) | chr1 | 0.350 | 0.459 | 0.109 | 0.0261 |
| 20 | cg07297153 | REPIN1 (-2012) | chr7 | 0.655 | 0.763 | 0.108 | 0.0085 |
| 21 | cg00956193 | ZNF770 (-95077) | chr15 | 0.450 | 0.557 | 0.107 | 0.0054 |
| 22 | cg13093842 | NLRP7 (-23) | chr19 | 0.600 | 0.706 | 0.106 | 0.0068 |
| 23 | cg04520169 | HLA-G (+99440) | chr6 | 0.137 | 0.239 | 0.102 | 0.0067 |
| 24 | cg02249577 | SGMS1 (-51036) | chr10 | 0.682 | 0.783 | 0.101 | 0.0054 |
| 25 | cg07655261 | NANOG (+6526) | chr12 | 0.490 | 0.591 | 0.101 | 0.0110 |
| 26 | cg03447150 | SEMA5A (+662041) | chr5 | 0.546 | 0.646 | 0.100 | 0.0244 |
| 27 | cg08951186 | ZNF469 (-203509) | chr16 | 0.536 | 0.635 | 0.100 | 0.0087 |
| 28 | cg24680439 | TTC40 (-22379) | chr10 | 0.505 | 0.604 | 0.099 | 0.0260 |
| 29 | cg26175789 | EVA1A (-25045) | chr2 | 0.391 | 0.490 | 0.099 | 0.0062 |
| 30 | cg06013395 | EPHB3 (+1141) | chr3 | 0.668 | 0.766 | 0.099 | 0.0072 |
| 31 | cg18060330 | BTNL2 (-1162) | chr6 | 0.589 | 0.686 | 0.097 | 0.0147 |
| 32 | cg08778598 | LPCAT1 (-70488) | chr5 | 0.168 | 0.263 | 0.096 | 0.0188 |
| 33 | cg07973162 | UGT2B17 (-1006) | chr4 | 0.413 | 0.509 | 0.096 | 0.0200 |
| 34 | cg06221963 | KCNN3 (+2943) | chr1 | 0.298 | 0.394 | 0.096 | 0.0028 |
| 35 | cg13166535 | LOXL4 (-28710) | chr10 | 0.459 | 0.554 | 0.095 | 0.0240 |
| 36 | cg08231349 | HLA-G (+99889) | chr6 | 0.052 | 0.146 | 0.095 | 0.0006 |
| 37 | cg07748963 | LPHN2 (+361581) | chr1 | 0.316 | 0.411 | 0.095 | 0.0299 |
| 38 | cg13365324 | UGT2B17 (-1229) | chr4 | 0.431 | 0.525 | 0.094 | 0.0290 |
| 39 | cg12088417 | RPTOR (+55649) | chr17 | 0.754 | 0.848 | 0.094 | 0.0411 |
| 40 | cg03806328 | ARHGAP32 (-67539) | chr11 | 0.594 | 0.687 | 0.094 | 0.0015 |
| 41 | cg07952421 | UGT2B17 (-1357) | chr4 | 0.529 | 0.622 | 0.093 | 0.0183 |
| 42 | cg24239165 | C17orf82 (+7542) | chr17 | 0.583 | 0.675 | 0.092 | 0.0221 |
| 43 | cg09359103 | KCNN3 (+2847) | chr1 | 0.240 | 0.331 | 0.092 | 0.0022 |
| 44 | cg17370981 | ATP4B (+918) | chr13 | 0.445 | 0.535 | 0.090 | 0.0344 |
| 45 | cg10388667 | MRPL53 (+2528) | chr2 | 0.692 | 0.782 | 0.090 | 0.0171 |
| 46 | cg06871764 | BTNL2 (-1191) | chr6 | 0.518 | 0.607 | 0.089 | 0.0077 |
| 47 | cg27219185 | BTBD3 (-511455) | chr20 | 0.453 | 0.542 | 0.089 | 0.0463 |
| 48 | cg20302533 | POU6F2 (+153166) | chr7 | 0.263 | 0.352 | 0.089 | 0.0004 |
| 49 | cg27175123 | SEL1L (-121914) | chr14 | 0.617 | 0.706 | 0.089 | 0.0020 |
| 50 | cg18806707 | EPDR1 (+38254) | chr7 | 0.326 | 0.414 | 0.088 | 0.0122 |
| 51 | cg22742493 | SLC25A38 (-6194) | chr3 | 0.525 | 0.613 | 0.088 | 0.0276 |
| 52 | cg22772380 | ZNF12 (-484) | chr7 | 0.450 | 0.538 | 0.088 | 0.0010 |
| 53 | cg18734446 | GDNF (-38184) | chr5 | 0.653 | 0.739 | 0.086 | 0.0073 |
| 54 | cg14381313 | ZNF469 (-225540) | chr16 | 0.496 | 0.581 | 0.085 | 0.0098 |
| 55 | cg26353469 | HLA-G (+61215) | chr6 | 0.086 | 0.171 | 0.085 | 0.0007 |
| 56 | cg02947374 | ZNF469 (-226413) | chr16 | 0.561 | 0.645 | 0.084 | 0.0208 |
| 57 | cg15727171 | NONE | chr9 | 0.241 | 0.325 | 0.084 | 0.0019 |
| 58 | cg04485391 | ZMIZ1 (-327344) | chr10 | 0.526 | 0.610 | 0.084 | 0.0161 |
| 59 | cg14904295 | PEG3 (+3535) | chr19 | 0.295 | 0.379 | 0.083 | 0.0037 |
| 60 | cg03660162 | MAF (+721526) | chr16 | 0.713 | 0.796 | 0.083 | 0.0207 |
| 61 | cg07346359 | OPTC (+26398) | chr1 | 0.427 | 0.509 | 0.083 | 0.0005 |
| 62 | cg21549632 | HLA-G (+61532) | chr6 | 0.066 | 0.149 | 0.083 | 0.0004 |
| 63 | cg03995122 | HLA-G (+99887) | chr6 | 0.030 | 0.112 | 0.082 | 0.0008 |
| 64 | cg13132497 | LYSMD4 (-195527) | chr15 | 0.464 | 0.544 | 0.080 | 0.0321 |
| 65 | cg00621508 | SLC17A2 (+4745) | chr6 | 0.603 | 0.684 | 0.080 | 0.0250 |
| 66 | cg20744362 | BRD1 (+168288) | chr22 | 0.473 | 0.553 | 0.080 | 0.0235 |
| 67 | cg18768136 | ASAH2 (-87699) | chr10 | 0.436 | 0.516 | 0.080 | 0.0009 |
| 68 | cg07113537 | DAZL (+3439) | chr3 | 0.137 | 0.217 | 0.080 | 0.0001 |
| 69 | cg25835058 | TMEM51 (-71271) | chr1 | 0.422 | 0.502 | 0.080 | 0.0109 |
| 70 | cg00935887 | CXXC11 (+32120) | chr2 | 0.446 | 0.526 | 0.079 | 0.0178 |
| 71 | cg15671450 | HLA-G (+100361) | chr6 | 0.090 | 0.170 | 0.079 | 0.0107 |
| 72 | cg06014707 | PI15 (-69154) | chr8 | 0.571 | 0.650 | 0.079 | 0.0028 |
| 73 | cg10983929 | FAM90A1 (+213) | chr12 | 0.445 | 0.523 | 0.079 | 0.0047 |
| 74 | cg15681295 | TTC40 (-22198) | chr10 | 0.497 | 0.575 | 0.079 | 0.0070 |
| 75 | cg08363235 | ASAH2 (-168462) | chr10 | 0.454 | 0.533 | 0.078 | 0.0007 |
| 76 | cg00413089 | WDR60 (+101717) | chr7 | 0.294 | 0.372 | 0.078 | 0.0223 |
| 77 | cg16302021 | HLA-G (+99189) | chr6 | 0.409 | 0.486 | 0.077 | 0.0016 |
| 78 | cg19196320 | DEFB115 (-322339) | chr20 | 0.573 | 0.650 | 0.077 | 0.0158 |
| 79 | cg00409917 | HLA-G (+99924) | chr6 | 0.074 | 0.150 | 0.077 | 0.0025 |
| 80 | cg23237314 | HLA-G (+99442) | chr6 | 0.132 | 0.209 | 0.076 | 0.0176 |
| 81 | cg18896979 | CXXC11 (+32294) | chr2 | 0.455 | 0.531 | 0.076 | 0.0177 |
| 82 | cg19783563 | SPACA7 (-45022) | chr13 | 0.323 | 0.399 | 0.076 | 0.0022 |
| 83 | cg19823512 | OPTC (+26455) | chr1 | 0.308 | 0.383 | 0.075 | 0.0011 |
| 84 | cg14091258 | DNAJB6 (+276948) | chr7 | 0.512 | 0.587 | 0.075 | 0.0067 |
| 85 | cg05237260 | CNTNAP3B (+717097) | chr9 | 0.291 | 0.366 | 0.075 | 0.0053 |
| 86 | cg16906964 | AJAP1 (-54934) | chr1 | 0.412 | 0.487 | 0.075 | 0.0040 |
| 87 | cg00845968 | ZMIZ1 (-318760) | chr10 | 0.578 | 0.653 | 0.075 | 0.0085 |
| 88 | cg14859874 | HAX1 (-6722) | chr1 | 0.091 | 0.166 | 0.075 | 0.0066 |
| 89 | cg05181157 | SNAPC1 (+47084) | chr14 | 0.615 | 0.689 | 0.075 | 0.0275 |
| 90 | cg01918803 | PSG2 (-36757) | chr19 | 0.538 | 0.613 | 0.074 | 0.0013 |
| 91 | cg02048733 | CBLB (-480477) | chr3 | 0.456 | 0.530 | 0.074 | 0.0016 |
| 92 | cg01719566 | FAM90A1 (-83) | chr12 | 0.470 | 0.544 | 0.074 | 0.0135 |
| 93 | cg02033694 | NPIPB11 (+118553) | chr16 | 0.284 | 0.358 | 0.074 | 0.0002 |
| 94 | cg09928274 | ELSPBP1 (+3822) | chr19 | 0.764 | 0.837 | 0.073 | 0.0313 |
| 95 | cg17155018 | TRAF3IP2 (-25618) | chr6 | 0.519 | 0.592 | 0.073 | 0.0060 |
| 96 | cg24236953 | FGFR2 (+41563) | chr10 | 0.496 | 0.569 | 0.073 | 0.0324 |
| 97 | cg17291189 | SLC2A14 (+25003) | chr12 | 0.346 | 0.418 | 0.073 | 0.0066 |
| 98 | cg09597767 | IFITM3 (-4866) | chr11 | 0.698 | 0.770 | 0.072 | 0.0037 |
| 99 | cg13266242 | HLA-G (+39740) | chr6 | 0.551 | 0.623 | 0.072 | 0.0014 |
| 100 | cg01336390 | HLA-G (+100304) | chr6 | 0.086 | 0.158 | 0.072 | 0.0048 |
| 101 | cg15532640 | GLT1D1 (+216486) | chr12 | 0.305 | 0.376 | 0.071 | 0.0382 |
| 102 | cg03410772 | ZNRD1 (-59026) | chr6 | 0.665 | 0.736 | 0.071 | 0.0000 |
| 103 | cg25539628 | ENSG00000182319 (+55525) | chr8 | 0.300 | 0.371 | 0.071 | 0.0004 |
| 104 | cg15780967 | PPP2R5E (+76068) | chr14 | 0.580 | 0.651 | 0.071 | 0.0377 |
| 105 | cg20744163 | PPIF (-107393) | chr10 | 0.665 | 0.736 | 0.071 | 0.0413 |
| 106 | cg26028489 | LMO3 (+85999) | chr12 | 0.485 | 0.556 | 0.071 | 0.0027 |
| 107 | cg25877386 | DHX37 (-1348) | chr12 | 0.755 | 0.825 | 0.071 | 0.0361 |
| 108 | cg17341345 | CCNH (-574) | chr5 | 0.510 | 0.580 | 0.071 | 0.0116 |
| 109 | cg16223220 | HLA-G (+61159) | chr6 | 0.042 | 0.112 | 0.071 | 0.0017 |
| 110 | cg09646655 | ZFP37 (-2357) | chr9 | 0.481 | 0.551 | 0.070 | 0.0102 |
| 111 | cg10632656 | UGT2B17 (-1349) | chr4 | 0.278 | 0.348 | 0.070 | 0.0192 |
| 112 | cg00661861 | GPRIN2 (-3909) | chr10 | 0.591 | 0.660 | 0.069 | 0.0381 |
| 113 | cg12038583 | ZNF727 (-476334) | chr7 | 0.484 | 0.554 | 0.069 | 0.0181 |
| 114 | cg05202858 | FGF12 (+352912) | chr3 | 0.408 | 0.477 | 0.069 | 0.0330 |
| 115 | cg22968327 | NUP93 (+59405) | chr16 | 0.262 | 0.331 | 0.069 | 0.0178 |
| 116 | cg14449180 | HLA-G (+99864) | chr6 | 0.039 | 0.108 | 0.069 | 0.0021 |
| 117 | cg08993878 | TMPO (-758029) | chr12 | 0.355 | 0.424 | 0.069 | 0.0301 |
| 118 | cg05246613 | SH3RF3 (+9250) | chr2 | 0.302 | 0.370 | 0.069 | 0.0088 |
| 119 | cg17746638 | ENSG00000233024 (-7835) | chr16 | 0.541 | 0.609 | 0.069 | 0.0219 |
| 120 | cg09199338 | AGA (-311227) | chr4 | 0.270 | 0.338 | 0.069 | 0.0003 |
| 121 | cg07318398 | NT5C3B (-587) | chr17 | 0.338 | 0.406 | 0.068 | 0.0043 |
| 122 | cg03977382 | CNTN5 (+672992) | chr11 | 0.574 | 0.643 | 0.068 | 0.0085 |
| 123 | cg15411272 | HLA-G (+100432) | chr6 | 0.284 | 0.352 | 0.068 | 0.0392 |
| 124 | cg01499815 | HLA-G (+100319) | chr6 | 0.087 | 0.156 | 0.068 | 0.0028 |
| 125 | cg08422420 | LPCAT1 (-70642) | chr5 | 0.145 | 0.213 | 0.068 | 0.0205 |
| 126 | cg26274304 | NCAPG2 (+942) | chr7 | 0.273 | 0.341 | 0.068 | 0.0042 |
| 127 | cg24289952 | ZNF80 (+14382) | chr3 | 0.474 | 0.542 | 0.068 | 0.0331 |
| 128 | cg21963583 | MRPL21 (+12452) | chr11 | 0.399 | 0.467 | 0.068 | 0.0303 |
| 129 | cg21138405 | IRF1 (-1318) | chr5 | 0.226 | 0.294 | 0.068 | 0.0247 |
| 130 | cg09801012 | ASAH2 (-127080) | chr10 | 0.644 | 0.712 | 0.067 | 0.0049 |
| 131 | cg13597893 | UBE2E3 (-272789) | chr2 | 0.498 | 0.565 | 0.067 | 0.0009 |
| 132 | cg11047325 | SOCS3 (+1224) | chr17 | 0.481 | 0.548 | 0.067 | 0.0042 |
| 133 | cg14372705 | GSC2 (-3235) | chr22 | 0.490 | 0.557 | 0.067 | 0.0470 |
| 134 | cg23703303 | SPAG11A (+14714) | chr8 | 0.545 | 0.611 | 0.067 | 0.0107 |
| 135 | cg03071500 | IFITM3 (-4915) | chr11 | 0.559 | 0.626 | 0.067 | 0.0227 |
| 136 | cg05187965 | TMEM72 (+117) | chr10 | 0.553 | 0.619 | 0.067 | 0.0142 |
| 137 | cg15878909 | FAM90A1 (-73) | chr12 | 0.399 | 0.465 | 0.066 | 0.0240 |
| 138 | cg00255919 | IRF1 (-1429) | chr5 | 0.234 | 0.300 | 0.066 | 0.0153 |
| 139 | cg25952247 | LHX3 (+2131) | chr9 | 0.413 | 0.479 | 0.066 | 0.0099 |
| 140 | cg11406274 | HLA-G (+61809) | chr6 | 0.253 | 0.319 | 0.066 | 0.0040 |
| 141 | cg10621924 | POU6F2 (+153473) | chr7 | 0.692 | 0.758 | 0.066 | 0.0016 |
| 142 | cg03181300 | HIST1H2AD (+3476) | chr6 | 0.220 | 0.285 | 0.066 | 0.0288 |
| 143 | cg04034577 | AGXT (+28480) | chr2 | 0.418 | 0.484 | 0.066 | 0.0177 |
| 144 | cg27149073 | LPCAT1 (-70239) | chr5 | 0.134 | 0.199 | 0.066 | 0.0077 |
| 145 | cg04972766 | NONE | chr5 | 0.466 | 0.531 | 0.066 | 0.0003 |
| 146 | cg03607220 | HLA-DRB5 (-28200) | chr6 | 0.564 | 0.629 | 0.066 | 0.0440 |
| 147 | cg21257293 | ZFP37 (-5722) | chr9 | 0.393 | 0.459 | 0.066 | 0.0484 |
| 148 | cg08835755 | NKX1-1 (-114199) | chr4 | 0.474 | 0.540 | 0.065 | 0.0191 |
| 149 | cg08009379 | COBL (+125847) | chr7 | 0.331 | 0.396 | 0.065 | 0.0001 |
| 150 | cg12744031 | WDR60 (+101916) | chr7 | 0.318 | 0.383 | 0.065 | 0.0460 |
| 151 | cg15931205 | HLA-G (+100065) | chr6 | 0.060 | 0.125 | 0.065 | 0.0003 |
| 152 | cg23490161 | SDHA (+97) | chr5 | 0.136 | 0.201 | 0.065 | 0.0240 |
| 153 | cg21665744 | POU6F2 (+153516) | chr7 | 0.637 | 0.701 | 0.065 | 0.0083 |
| 154 | cg18191116 | STON2 (+241) | chr14 | 0.508 | 0.572 | 0.065 | 0.0093 |
| 155 | cg20696345 | HS1BP3 (+39466) | chr2 | 0.415 | 0.480 | 0.065 | 0.0137 |
| 156 | cg16666458 | FAM126A (+72462) | chr7 | 0.588 | 0.652 | 0.065 | 0.0005 |
| 157 | cg18146737 | GFI1 (+2811) | chr1 | 0.705 | 0.769 | 0.064 | 0.0432 |
| 158 | cg05090351 | CTBP2 (-134704) | chr10 | 0.461 | 0.525 | 0.064 | 0.0096 |
| 159 | cg22253032 | PRSS1 (-37378) | chr7 | 0.436 | 0.500 | 0.064 | 0.0005 |
| 160 | cg08757828 | CPT1A (-28717) | chr11 | 0.290 | 0.354 | 0.064 | 0.0227 |
| 161 | cg03877706 | NCAM2 (+202000) | chr21 | 0.544 | 0.607 | 0.063 | 0.0111 |
| 162 | cg15011775 | RARB (+13136) | chr3 | 0.505 | 0.569 | 0.063 | 0.0020 |
| 163 | cg06025105 | RSPH1 (+21549) | chr21 | 0.701 | 0.764 | 0.063 | 0.0476 |
| 164 | cg27421994 | NAB1 (+9952) | chr2 | 0.363 | 0.426 | 0.063 | 0.0027 |
| 165 | cg02748618 | ATPAF2 (+10470) | chr17 | 0.370 | 0.433 | 0.063 | 0.0016 |
| 166 | cg19954471 | SPACA7 (+66683) | chr13 | 0.406 | 0.469 | 0.063 | 0.0009 |
| 167 | cg00186468 | CCNH (-393) | chr5 | 0.250 | 0.313 | 0.063 | 0.0011 |
| 168 | cg17184855 | HLA-G (+595) | chr6 | 0.607 | 0.670 | 0.063 | 0.0008 |
| 169 | cg21862992 | MRPL21 (+12905) | chr11 | 0.409 | 0.471 | 0.063 | 0.0432 |
| 170 | cg10077346 | BCKDHB (+104095) | chr6 | 0.481 | 0.543 | 0.063 | 0.0333 |
| 171 | cg06752595 | ALDH5A1 (-1115) | chr6 | 0.530 | 0.592 | 0.062 | 0.0464 |
| 172 | cg27584762 | CCNH (-291) | chr5 | 0.208 | 0.271 | 0.062 | 0.0022 |
| 173 | cg05332308 | CDH8 (-372226) | chr16 | 0.439 | 0.501 | 0.062 | 0.0047 |
| 174 | cg04535902 | GFI1 (+2179) | chr1 | 0.632 | 0.694 | 0.062 | 0.0248 |
| 175 | cg25897043 | ITGB1 (-95701) | chr10 | 0.511 | 0.573 | 0.062 | 0.0057 |
| 176 | cg27209610 | ST3GAL5 (-102063) | chr2 | 0.454 | 0.516 | 0.062 | 0.0041 |
| 177 | cg14223671 | GNG13 (-7249) | chr16 | 0.142 | 0.205 | 0.062 | 0.0065 |
| 178 | cg15598217 | ZZEF1 (+44435) | chr17 | 0.530 | 0.592 | 0.062 | 0.0058 |
| 179 | cg01668281 | CLDN14 (-62894) | chr21 | 0.272 | 0.334 | 0.062 | 0.0016 |
| 180 | cg01324550 | HOXB5 (-4777) | chr17 | 0.355 | 0.417 | 0.062 | 0.0016 |
| 181 | cg11365170 | SEMA6A (+77177) | chr5 | 0.590 | 0.652 | 0.062 | 0.0015 |
| 182 | cg27618398 | DCLK1 (-27638) | chr13 | 0.647 | 0.709 | 0.062 | 0.0458 |
| 183 | cg00135841 | USP18 (+104683) | chr22 | 0.766 | 0.828 | 0.062 | 0.0110 |
| 184 | cg19748509 | HLA-G (+116023) | chr6 | 0.071 | 0.133 | 0.062 | 0.0014 |
| 185 | cg03551406 | MT1X (-580) | chr16 | 0.444 | 0.506 | 0.062 | 0.0006 |
| 186 | cg01617603 | UGT2B17 (+15538) | chr4 | 0.247 | 0.309 | 0.062 | 0.0185 |
| 187 | cg11449146 | GAS2 (+62304) | chr11 | 0.560 | 0.622 | 0.061 | 0.0026 |
| 188 | cg00369056 | NPIPA7 (+490096) | chr16 | 0.658 | 0.719 | 0.061 | 0.0223 |
| 189 | cg19684894 | C1orf222 (+11284) | chr1 | 0.097 | 0.158 | 0.061 | 0.0379 |
| 190 | cg10031873 | ALG10B (-166540) | chr12 | 0.732 | 0.793 | 0.061 | 0.0003 |
| 191 | cg27540865 | CEP170 (+364418) | chr1 | 0.189 | 0.250 | 0.061 | 0.0056 |
| 192 | cg12439472 | DNAJC15 (-31940) | chr13 | 0.497 | 0.558 | 0.061 | 0.0182 |
| 193 | cg02556954 | ETF1 (+30412) | chr5 | 0.483 | 0.544 | 0.061 | 0.0010 |
| 194 | cg27119318 | WRB (+7405) | chr21 | 0.171 | 0.232 | 0.061 | 0.0033 |
| 195 | cg24699985 | C1orf174 (-276752) | chr1 | 0.462 | 0.522 | 0.060 | 0.0024 |
| 196 | cg16680214 | KCNN3 (+2773) | chr1 | 0.181 | 0.241 | 0.060 | 0.0004 |
| 197 | cg03249561 | FCAR (+14427) | chr19 | 0.678 | 0.738 | 0.060 | 0.0371 |
| 198 | cg12545480 | SYT14 (-12090) | chr1 | 0.405 | 0.465 | 0.060 | 0.0309 |
| 199 | cg23248910 | FGFR2 (+87847) | chr10 | 0.469 | 0.529 | 0.060 | 0.0177 |
| 200 | cg11351709 | TDRP (-333843) | chr8 | 0.418 | 0.478 | 0.060 | 0.0271 |
| 201 | cg19061000 | GALC (+182752) | chr14 | 0.560 | 0.620 | 0.060 | 0.0045 |
| 202 | cg03618918 | ITLN1 (-10138) | chr1 | 0.653 | 0.713 | 0.060 | 0.0004 |
| 203 | cg16681436 | EGFL8 (+2860) | chr6 | 0.694 | 0.753 | 0.060 | 0.0209 |
| 204 | cg02447462 | CFD (+1619) | chr19 | 0.403 | 0.462 | 0.060 | 0.0175 |
| 205 | cg19686152 | TMOD3 (-285) | chr15 | 0.458 | 0.517 | 0.060 | 0.0484 |
| 206 | cg24960960 | LPCAT1 (-70587) | chr5 | 0.171 | 0.230 | 0.059 | 0.0169 |
| 207 | cg15218522 | SBSPON (-75679) | chr8 | 0.339 | 0.398 | 0.059 | 0.0137 |
| 208 | cg02850689 | PLCH2 (-7551) | chr1 | 0.439 | 0.499 | 0.059 | 0.0026 |
| 209 | cg21167402 | LPCAT1 (-70585) | chr5 | 0.217 | 0.277 | 0.059 | 0.0214 |
| 210 | cg02015053 | EIF3J (+24728) | chr15 | 0.412 | 0.471 | 0.059 | 0.0039 |
| 211 | cg22872376 | MAGEL2 (-2568) | chr15 | 0.310 | 0.369 | 0.059 | 0.0015 |
| 212 | cg06611532 | RASA3 (-1936) | chr13 | 0.436 | 0.495 | 0.059 | 0.0423 |
| 213 | cg03589715 | SLC8A3 (-34563) | chr14 | 0.620 | 0.679 | 0.059 | 0.0256 |
| 214 | cg01719179 | NPIPB11 (+118657) | chr16 | 0.221 | 0.280 | 0.059 | 0.0004 |
| 215 | cg05037738 | ATP13A3 (-1381) | chr3 | 0.531 | 0.590 | 0.059 | 0.0149 |
| 216 | cg21894124 | FBRSL1 (-161448) | chr12 | 0.148 | 0.207 | 0.059 | 0.0006 |
| 217 | cg06112835 | MRPL21 (+12495) | chr11 | 0.298 | 0.357 | 0.059 | 0.0296 |
| 218 | cg13488542 | MPHOSPH6 (+17514) | chr16 | 0.669 | 0.728 | 0.059 | 0.0152 |
| 219 | cg08835956 | POU6F2 (+153437) | chr7 | 0.639 | 0.698 | 0.059 | 0.0086 |
| 220 | cg07962847 | ACTR3B (+531378) | chr7 | 0.390 | 0.449 | 0.059 | 0.0233 |
| 221 | cg14079719 | IPCEF1 (+100117) | chr6 | 0.420 | 0.479 | 0.059 | 0.0088 |
| 222 | cg20370296 | ZNF251 (+69043) | chr8 | 0.352 | 0.411 | 0.059 | 0.0184 |
| 223 | cg03785755 | HIST1H2AD (+2677) | chr6 | 0.360 | 0.419 | 0.059 | 0.0038 |
| 224 | cg05845204 | RBM39 (+12594) | chr20 | 0.733 | 0.792 | 0.059 | 0.0182 |
| 225 | cg09274344 | SOX8 (+46882) | chr16 | 0.315 | 0.373 | 0.059 | 0.0136 |
| 226 | cg15111296 | CXXC1 (-33327) | chr18 | 0.545 | 0.603 | 0.058 | 0.0466 |
| 227 | cg24126361 | SLC25A37 (+12029) | chr8 | 0.499 | 0.558 | 0.058 | 0.0423 |
| 228 | cg01062395 | HLA-G (+60881) | chr6 | 0.111 | 0.170 | 0.058 | 0.0060 |
| 229 | cg05875302 | CXXC11 (+32901) | chr2 | 0.695 | 0.753 | 0.058 | 0.0023 |
| 230 | cg17590488 | NEBL (-132505) | chr10 | 0.447 | 0.505 | 0.058 | 0.0089 |
| 231 | cg26188685 | HDC (+17954) | chr15 | 0.220 | 0.278 | 0.058 | 0.0021 |
| 232 | cg20748132 | SCARB1 (+130686) | chr12 | 0.474 | 0.532 | 0.058 | 0.0075 |
| 233 | cg11851257 | TAC4 (+21973) | chr17 | 0.579 | 0.637 | 0.058 | 0.0014 |
| 234 | cg05636112 | KYNU (+47533) | chr2 | 0.488 | 0.546 | 0.058 | 0.0196 |
| 235 | cg11284736 | ENSG00000166503 (+50062) | chr15 | 0.603 | 0.661 | 0.058 | 0.0003 |
| 236 | cg09239700 | IRF4 (+58348) | chr6 | 0.604 | 0.662 | 0.058 | 0.0033 |
| 237 | cg07872519 | ABCB1 (-301) | chr7 | 0.459 | 0.517 | 0.057 | 0.0021 |
| 238 | cg01889129 | PDZD8 (-63910) | chr10 | 0.505 | 0.562 | 0.057 | 0.0063 |
| 239 | cg15534755 | TAGLN (-2730) | chr11 | 0.574 | 0.631 | 0.057 | 0.0161 |
| 240 | cg05900440 | TTC30A (+19814) | chr2 | 0.441 | 0.498 | 0.057 | 0.0145 |
| 241 | cg10814153 | ODF3L2 (+10) | chr19 | 0.578 | 0.635 | 0.057 | 0.0103 |
| 242 | cg09874992 | SDC3 (+67000) | chr1 | 0.376 | 0.434 | 0.057 | 0.0044 |
| 243 | cg12798157 | AKR7A3 (+15025) | chr1 | 0.185 | 0.242 | 0.057 | 0.0159 |
| 244 | cg05452645 | PRDM8 (+11224) | chr4 | 0.348 | 0.405 | 0.057 | 0.0426 |
| 245 | cg14643763 | STK35 (+71468) | chr20 | 0.387 | 0.444 | 0.057 | 0.0146 |
| 246 | cg13126979 | EME2 (+970) | chr16 | 0.232 | 0.289 | 0.057 | 0.0045 |
| 247 | cg01876548 | ZNF608 (-45746) | chr5 | 0.560 | 0.617 | 0.057 | 0.0068 |
| 248 | cg03594447 | PLA2G5 (-36957) | chr1 | 0.292 | 0.348 | 0.057 | 0.0016 |
| 249 | cg08121845 | NONE | chr3 | 0.261 | 0.318 | 0.057 | 0.0017 |
| 250 | cg04124281 | MTHFS (-747) | chr15 | 0.229 | 0.286 | 0.057 | 0.0047 |
| 251 | cg03701930 | ADARB2 (-201767) | chr10 | 0.144 | 0.200 | 0.057 | 0.0014 |
| 252 | cg07324633 | LPCAT1 (-44578) | chr5 | 0.431 | 0.488 | 0.057 | 0.0012 |
| 253 | cg23420995 | DCLK1 (-2777) | chr13 | 0.171 | 0.227 | 0.057 | 0.0010 |
| 254 | cg18181703 | SOCS3 (+1537) | chr17 | 0.307 | 0.363 | 0.057 | 0.0004 |
| 255 | cg19167230 | NONE | chr4 | 0.344 | 0.401 | 0.056 | 0.0015 |
| 256 | cg11861562 | TAGLN (-2809) | chr11 | 0.540 | 0.596 | 0.056 | 0.0135 |
| 257 | cg27029450 | BRD1 (+120378) | chr22 | 0.339 | 0.396 | 0.056 | 0.0013 |
| 258 | cg18943014 | ZNF92 (+89579) | chr7 | 0.375 | 0.431 | 0.056 | 0.0018 |
| 259 | cg20681948 | ASCL1 (-9167) | chr12 | 0.373 | 0.429 | 0.056 | 0.0034 |
| 260 | cg15817705 | CAMK1G (-350999) | chr1 | 0.537 | 0.594 | 0.056 | 0.0018 |
| 261 | cg16884706 | TAS1R2 (+66273) | chr1 | 0.462 | 0.518 | 0.056 | 0.0011 |
| 262 | cg07782112 | CHAC2 (-887087) | chr2 | 0.391 | 0.447 | 0.056 | 0.0229 |
| 263 | cg17309085 | CNTN5 (+672975) | chr11 | 0.681 | 0.737 | 0.056 | 0.0096 |
| 264 | cg14864167 | PDE7A (+3005) | chr8 | 0.388 | 0.444 | 0.056 | 0.0014 |
| 265 | cg22872396 | SCN9A (+9830) | chr2 | 0.507 | 0.562 | 0.055 | 0.0150 |
| 266 | cg11807280 | MEIS1 (-7888) | chr2 | 0.296 | 0.352 | 0.055 | 0.0036 |
| 267 | cg17361885 | MXRA7 (+6038) | chr17 | 0.669 | 0.725 | 0.055 | 0.0438 |
| 268 | cg13363596 | CLDN14 (-62926) | chr21 | 0.394 | 0.449 | 0.055 | 0.0036 |
| 269 | cg10773881 | GGA1 (+15331) | chr22 | 0.118 | 0.173 | 0.055 | 0.0000 |
| 270 | cg23962358 | CPZ (+79985) | chr4 | 0.402 | 0.457 | 0.055 | 0.0099 |
| 271 | cg18737081 | PPIF (-107427) | chr10 | 0.624 | 0.679 | 0.055 | 0.0280 |
| 272 | cg04118610 | LPHN3 (+639168) | chr4 | 0.106 | 0.161 | 0.055 | 0.0309 |
| 273 | cg00417819 | SYT3 (+33140) | chr19 | 0.579 | 0.633 | 0.055 | 0.0009 |
| 274 | cg13294652 | OBSCN (+20917) | chr1 | 0.353 | 0.408 | 0.055 | 0.0158 |
| 275 | cg23299919 | DNAJB6 (+276437) | chr7 | 0.335 | 0.389 | 0.055 | 0.0185 |
| 276 | cg13470831 | SALL3 (-59897) | chr18 | 0.504 | 0.558 | 0.054 | 0.0058 |
| 277 | cg10464773 | QKI (+335832) | chr6 | 0.479 | 0.534 | 0.054 | 0.0121 |
| 278 | cg20476087 | CGREF1 (-126) | chr2 | 0.115 | 0.170 | 0.054 | 0.0002 |
| 279 | cg27404186 | CXXC11 (+31941) | chr2 | 0.661 | 0.716 | 0.054 | 0.0133 |
| 280 | cg08206881 | LPIN1 (+499637) | chr2 | 0.124 | 0.178 | 0.054 | 0.0167 |
| 281 | cg01176694 | MKL2 (+208587) | chr16 | 0.440 | 0.495 | 0.054 | 0.0009 |
| 282 | cg12019814 | RAD21 (+25858) | chr8 | 0.376 | 0.431 | 0.054 | 0.0015 |
| 283 | cg26942295 | PDZD2 (+49191) | chr5 | 0.498 | 0.552 | 0.054 | 0.0125 |
| 284 | cg02407415 | GNG13 (-6722) | chr16 | 0.207 | 0.261 | 0.054 | 0.0429 |
| 285 | cg23340194 | USP14 (-3299) | chr18 | 0.484 | 0.538 | 0.054 | 0.0390 |
| 286 | cg14827090 | BEGAIN (+42983) | chr14 | 0.520 | 0.574 | 0.054 | 0.0233 |
| 287 | cg25711786 | ETF1 (+24486) | chr5 | 0.417 | 0.471 | 0.054 | 0.0018 |
| 288 | cg14080482 | NONE | chr6 | 0.389 | 0.443 | 0.054 | 0.0004 |
| 289 | cg08937153 | CEBPD (+146838) | chr8 | 0.609 | 0.663 | 0.054 | 0.0010 |
| 290 | cg21705926 | DNAJB6 (+276978) | chr7 | 0.488 | 0.542 | 0.054 | 0.0285 |
| 291 | cg11124651 | NONE | chr19 | 0.655 | 0.709 | 0.054 | 0.0132 |
| 292 | cg17565702 | KIFC2 (-138) | chr8 | 0.457 | 0.511 | 0.054 | 0.0029 |
| 293 | cg19727165 | DHFRL1 (-114061) | chr3 | 0.613 | 0.667 | 0.054 | 0.0436 |
| 294 | cg14088970 | ENGASE (+140423) | chr17 | 0.342 | 0.396 | 0.054 | 0.0094 |
| 295 | cg21733854 | ZSCAN22 (-615) | chr19 | 0.523 | 0.577 | 0.054 | 0.0078 |
| 296 | cg00242965 | MRPL21 (+12319) | chr11 | 0.271 | 0.325 | 0.054 | 0.0396 |
| 297 | cg18449879 | CYP4F11 (+591) | chr19 | 0.487 | 0.540 | 0.054 | 0.0169 |
| 298 | cg23917496 | UACA (+300798) | chr15 | 0.383 | 0.437 | 0.054 | 0.0276 |
| 299 | cg19852147 | WWOX (+662174) | chr16 | 0.311 | 0.365 | 0.054 | 0.0061 |
| 300 | cg25282559 | NONE | chr14 | 0.468 | 0.522 | 0.054 | 0.0011 |
| 301 | cg24693741 | DYM (+101009) | chr18 | 0.237 | 0.290 | 0.054 | 0.0009 |
| 302 | cg12607525 | ATXN7L3 (-11412) | chr17 | 0.559 | 0.613 | 0.054 | 0.0042 |
| 303 | cg05333568 | C1orf65 (+80) | chr1 | 0.256 | 0.309 | 0.053 | 0.0035 |
| 304 | cg12884719 | SIPA1L2 (+451040) | chr1 | 0.424 | 0.477 | 0.053 | 0.0066 |
| 305 | cg10358342 | NOBOX (-307) | chr7 | 0.315 | 0.368 | 0.053 | 0.0004 |
| 306 | cg19445457 | OR52N5 (+451) | chr11 | 0.536 | 0.589 | 0.053 | 0.0110 |
| 307 | cg01256539 | PRR16 (+2004) | chr5 | 0.510 | 0.563 | 0.053 | 0.0116 |
| 308 | cg09142843 | ARHGAP18 (+389699) | chr6 | 0.740 | 0.793 | 0.053 | 0.0448 |
| 309 | cg13056744 | HLA-G (+100185) | chr6 | 0.118 | 0.171 | 0.053 | 0.0073 |
| 310 | cg06588529 | ECI2 (-18261) | chr6 | 0.655 | 0.708 | 0.053 | 0.0232 |
| 311 | cg00666877 | CEP112 (+190945) | chr17 | 0.553 | 0.606 | 0.053 | 0.0028 |
| 312 | cg11706815 | MBD3L1 (+59748) | chr19 | 0.396 | 0.449 | 0.053 | 0.0274 |
| 313 | cg03362483 | LDLRAP1 (-23383) | chr1 | 0.352 | 0.405 | 0.053 | 0.0095 |
| 314 | cg15853715 | C14orf39 (-2535) | chr14 | 0.452 | 0.505 | 0.053 | 0.0060 |
| 315 | cg25556464 | STOML1 (+17620) | chr15 | 0.551 | 0.603 | 0.053 | 0.0113 |
| 316 | cg22083892 | KCNJ8 (-907) | chr12 | 0.347 | 0.400 | 0.053 | 0.0172 |
| 317 | cg19684207 | WRB (+7517) | chr21 | 0.165 | 0.217 | 0.052 | 0.0050 |
| 318 | cg03904042 | NECAB3 (+6723) | chr20 | 0.213 | 0.266 | 0.052 | 0.0364 |
| 319 | cg25220979 | IGF2BP1 (+17499) | chr17 | 0.481 | 0.533 | 0.052 | 0.0002 |
| 320 | cg18018313 | IRS1 (+7283) | chr2 | 0.322 | 0.374 | 0.052 | 0.0005 |
| 321 | cg12700863 | IRX4 (-244322) | chr5 | 0.516 | 0.568 | 0.052 | 0.0047 |
| 322 | cg19318330 | SIK1 (+64136) | chr21 | 0.285 | 0.338 | 0.052 | 0.0059 |
| 323 | cg13434361 | PRSS16 (-18100) | chr6 | 0.620 | 0.672 | 0.052 | 0.0337 |
| 324 | cg09125754 | POTEF (-8533) | chr2 | 0.377 | 0.429 | 0.052 | 0.0386 |
| 325 | cg10130564 | TAGLN (-2740) | chr11 | 0.602 | 0.654 | 0.052 | 0.0456 |
| 326 | cg23245007 | TNK2 (-59895) | chr3 | 0.580 | 0.633 | 0.052 | 0.0161 |
| 327 | cg08045932 | BHLHE23 (-21594) | chr20 | 0.419 | 0.471 | 0.052 | 0.0310 |
| 328 | cg05760951 | SPATA31A5 (+453801) | chr9 | 0.225 | 0.278 | 0.052 | 0.0051 |
| 329 | cg24531534 | R3HCC1 (+16742) | chr8 | 0.313 | 0.366 | 0.052 | 0.0320 |
| 330 | cg10609241 | MTHFD1 (+29577) | chr14 | 0.669 | 0.721 | 0.052 | 0.0014 |
| 331 | cg08975641 | COL5A2 (+85053) | chr2 | 0.500 | 0.552 | 0.052 | 0.0426 |
| 332 | cg25452172 | CYP27B1 (-1718) | chr12 | 0.568 | 0.620 | 0.052 | 0.0002 |
| 333 | cg10341310 | MTFR1 (+25233) | chr8 | 0.452 | 0.503 | 0.052 | 0.0053 |
| 334 | cg16543056 | ZFP42 (-236815) | chr4 | 0.443 | 0.495 | 0.052 | 0.0013 |
| 335 | cg13343932 | SOCS3 (+1097) | chr17 | 0.440 | 0.492 | 0.052 | 0.0129 |
| 336 | cg09790280 | TMEM229A (-316698) | chr7 | 0.574 | 0.625 | 0.052 | 0.0076 |
| 337 | cg11232815 | ATP6V0E2 (+176593) | chr7 | 0.431 | 0.482 | 0.052 | 0.0378 |
| 338 | cg02830496 | SLC25A51 (-25460) | chr9 | 0.299 | 0.350 | 0.052 | 0.0197 |
| 339 | cg06012695 | TRIM27 (+121173) | chr6 | 0.300 | 0.351 | 0.052 | 0.0045 |
| 340 | cg02260461 | USH2A (+35690) | chr1 | 0.467 | 0.519 | 0.051 | 0.0016 |
| 341 | cg02027518 | NDST3 (-201083) | chr4 | 0.530 | 0.581 | 0.051 | 0.0003 |
| 342 | cg13048967 | CXCR1 (+1595) | chr2 | 0.629 | 0.681 | 0.051 | 0.0017 |
| 343 | cg07490070 | ANKRD23 (+4307) | chr2 | 0.159 | 0.210 | 0.051 | 0.0049 |
| 344 | cg25664381 | STX18 (-33719) | chr4 | 0.228 | 0.280 | 0.051 | 0.0041 |
| 345 | cg24589936 | MTX1 (+2106) | chr1 | 0.410 | 0.462 | 0.051 | 0.0489 |
| 346 | cg06531573 | GNG13 (-6844) | chr16 | 0.180 | 0.232 | 0.051 | 0.0220 |
| 347 | cg23155965 | TTC40 (-22088) | chr10 | 0.786 | 0.837 | 0.051 | 0.0057 |
| 348 | cg01124420 | EDAR (+310) | chr2 | 0.401 | 0.453 | 0.051 | 0.0034 |
| 349 | cg23999422 | SIRT6 (+9135) | chr19 | 0.225 | 0.276 | 0.051 | 0.0005 |
| 350 | cg12761472 | CEP85L (-685) | chr6 | 0.586 | 0.637 | 0.051 | 0.0014 |
| 351 | cg20746451 | SMARCAD1 (-1059) | chr4 | 0.593 | 0.644 | 0.051 | 0.0478 |
| 352 | cg24931191 | STAC (-172977) | chr3 | 0.269 | 0.319 | 0.051 | 0.0044 |
| 353 | cg08695253 | ODF3L2 (+4) | chr19 | 0.473 | 0.524 | 0.051 | 0.0096 |
| 354 | cg03742947 | HLA-G (+61520) | chr6 | 0.068 | 0.119 | 0.051 | 0.0080 |
| 355 | cg25883179 | GABRG2 (+256684) | chr5 | 0.665 | 0.715 | 0.051 | 0.0003 |
| 356 | cg12552320 | SLC25A21 (+176861) | chr14 | 0.434 | 0.484 | 0.051 | 0.0015 |
| 357 | cg05707985 | PRSS1 (-26980) | chr7 | 0.299 | 0.350 | 0.051 | 0.0288 |
| 358 | cg04492858 | PPP2R2D (-189169) | chr10 | 0.244 | 0.295 | 0.051 | 0.0085 |
| 359 | cg10848692 | TCL1A (-121392) | chr14 | 0.250 | 0.301 | 0.051 | 0.0010 |
| 360 | cg15441605 | TYRP1 (+121209) | chr9 | 0.538 | 0.589 | 0.051 | 0.0024 |
| 361 | cg06902219 | HLA-G (+61539) | chr6 | 0.100 | 0.151 | 0.051 | 0.0026 |
| 362 | cg03307118 | MAP1LC3B2 (-41193) | chr12 | 0.663 | 0.713 | 0.051 | 0.0162 |
| 363 | cg12219752 | FAM189A2 (+56037) | chr9 | 0.534 | 0.585 | 0.051 | 0.0288 |
| 364 | cg09775918 | NONE | chr9 | 0.360 | 0.410 | 0.051 | 0.0399 |
| 365 | cg03226871 | MAP3K2 (+17083) | chr2 | 0.515 | 0.565 | 0.051 | 0.0033 |
| 366 | cg22499139 | HLA-G (-35038) | chr6 | 0.643 | 0.693 | 0.051 | 0.0065 |
| 367 | cg22542685 | ZNF624 (+36608) | chr17 | 0.562 | 0.612 | 0.051 | 0.0117 |
| 368 | cg16875568 | ITGBL1 (+68145) | chr13 | 0.453 | 0.503 | 0.050 | 0.0277 |
| 369 | cg18087694 | RFPL4A (-1134) | chr19 | 0.319 | 0.370 | 0.050 | 0.0119 |
| 370 | cg03626024 | WSCD2 (-1183) | chr12 | 0.452 | 0.502 | 0.050 | 0.0019 |
| 371 | cg21234506 | BCL2A1 (+656) | chr15 | 0.394 | 0.445 | 0.050 | 0.0008 |
| 372 | cg09680926 | CHD2 (-13636) | chr15 | 0.413 | 0.464 | 0.050 | 0.0005 |
| 373 | cg09004254 | RNF157 (-34042) | chr17 | 0.578 | 0.628 | 0.050 | 0.0148 |
| 374 | cg07583744 | AUTS2 (+294919) | chr7 | 0.313 | 0.363 | 0.050 | 0.0254 |
| 375 | cg05284887 | GJA5 (+12178) | chr1 | 0.527 | 0.578 | 0.050 | 0.0046 |
| 376 | cg22943590 | MEIS1 (-13735) | chr2 | 0.458 | 0.508 | 0.050 | 0.0387 |
| 377 | cg15221739 | GPR98 (+381941) | chr5 | 0.267 | 0.318 | 0.050 | 0.0027 |
| 378 | cg20787634 | UQCRB (+7604) | chr8 | 0.592 | 0.642 | 0.050 | 0.0128 |
| 379 | cg09906620 | CCNB2 (-21939) | chr15 | 0.786 | 0.736 | -0.050 | 0.0063 |
| 380 | cg00919702 | C1QL3 (+2653) | chr10 | 0.719 | 0.669 | -0.050 | 0.0231 |
| 381 | cg23223755 | LHFPL4 (-1364) | chr3 | 0.625 | 0.575 | -0.050 | 0.0024 |
| 382 | cg02512888 | CAMK1D (+66264) | chr10 | 0.637 | 0.587 | -0.050 | 0.0007 |
| 383 | cg26985140 | SLC23A2 (-848) | chr20 | 0.710 | 0.660 | -0.050 | 0.0019 |
| 384 | cg14222229 | HIST2H3PS2 (+262795) | chr1 | 0.322 | 0.272 | -0.050 | 0.0115 |
| 385 | cg09166973 | PRDM9 (-151) | chr5 | 0.707 | 0.656 | -0.050 | 0.0146 |
| 386 | cg05250352 | POR (+27235) | chr7 | 0.695 | 0.645 | -0.050 | 0.0010 |
| 387 | cg27019757 | PDCD4 (+18771) | chr10 | 0.715 | 0.665 | -0.050 | 0.0041 |
| 388 | cg11541587 | TPGS2 (-123951) | chr18 | 0.779 | 0.728 | -0.051 | 0.0014 |
| 389 | cg13795666 | DDX59 (+20818) | chr1 | 0.685 | 0.634 | -0.051 | 0.0075 |
| 390 | cg24821564 | KIR2DL1 (-591) | chr19 | 0.774 | 0.723 | -0.051 | 0.0270 |
| 391 | cg04161902 | BAI2 (+1728) | chr1 | 0.736 | 0.686 | -0.051 | 0.0043 |
| 392 | cg00804338 | TFDP1 (+179) | chr13 | 0.166 | 0.115 | -0.051 | 0.0038 |
| 393 | cg10085053 | NARS2 (-5329) | chr11 | 0.692 | 0.641 | -0.051 | 0.0105 |
| 394 | cg09886641 | SPESP1 (+180) | chr15 | 0.741 | 0.690 | -0.051 | 0.0018 |
| 395 | cg00496389 | OTOP1 (+88771) | chr4 | 0.748 | 0.697 | -0.051 | 0.0139 |
| 396 | cg23609713 | TRIM27 (+153477) | chr6 | 0.585 | 0.534 | -0.051 | 0.0045 |
| 397 | cg11524065 | ZCCHC8 (+21993) | chr12 | 0.834 | 0.783 | -0.051 | 0.0402 |
| 398 | cg10218876 | TNNT1 (+97) | chr19 | 0.243 | 0.192 | -0.051 | 0.0443 |
| 399 | cg12463722 | OR4D1 (+6) | chr17 | 0.734 | 0.682 | -0.051 | 0.0002 |
| 400 | cg23492448 | RPL38 (-113727) | chr17 | 0.598 | 0.547 | -0.051 | 0.0134 |
| 401 | cg06820102 | ZBTB38 (-2263) | chr3 | 0.665 | 0.614 | -0.051 | 0.0003 |
| 402 | cg15547703 | CSTB (+3154) | chr21 | 0.590 | 0.539 | -0.051 | 0.0018 |
| 403 | cg10556772 | HLA-G (+679) | chr6 | 0.406 | 0.354 | -0.051 | 0.0065 |
| 404 | cg24836607 | ASZ1 (-176) | chr7 | 0.679 | 0.627 | -0.051 | 0.0096 |
| 405 | cg26259926 | LRRC24 (-3573) | chr8 | 0.655 | 0.604 | -0.051 | 0.0294 |
| 406 | cg03203155 | SCAND3 (-154692) | chr6 | 0.681 | 0.630 | -0.052 | 0.0152 |
| 407 | cg10082647 | C12orf23 (-645) | chr12 | 0.690 | 0.638 | -0.052 | 0.0049 |
| 408 | cg14841443 | RGS9 (+92958) | chr17 | 0.543 | 0.492 | -0.052 | 0.0457 |
| 409 | cg02109003 | HSBP1 (-106440) | chr16 | 0.552 | 0.500 | -0.052 | 0.0051 |
| 410 | cg24045276 | NCF2 (+7621) | chr1 | 0.658 | 0.606 | -0.052 | 0.0035 |
| 411 | cg20212775 | SPON2 (+1980) | chr4 | 0.643 | 0.591 | -0.052 | 0.0008 |
| 412 | cg09598512 | XYLB (-17726) | chr3 | 0.602 | 0.550 | -0.052 | 0.0254 |
| 413 | cg20050113 | SLC9A2 (+696) | chr2 | 0.387 | 0.334 | -0.052 | 0.0092 |
| 414 | cg17623013 | FAM81B (+81020) | chr5 | 0.856 | 0.804 | -0.052 | 0.0172 |
| 415 | cg08121925 | FXR1 (-42049) | chr3 | 0.319 | 0.267 | -0.052 | 0.0009 |
| 416 | cg23522194 | CABP5 (-17880) | chr19 | 0.786 | 0.734 | -0.052 | 0.0452 |
| 417 | cg02675179 | ACSM1 (+16505) | chr16 | 0.518 | 0.466 | -0.052 | 0.0093 |
| 418 | cg12847536 | ARHGEF10 (+50045) | chr8 | 0.641 | 0.589 | -0.052 | 0.0147 |
| 419 | cg22898160 | NDRG4 (-1339) | chr16 | 0.666 | 0.614 | -0.052 | 0.0018 |
| 420 | cg08087268 | CHGA (+16658) | chr14 | 0.619 | 0.567 | -0.052 | 0.0218 |
| 421 | cg26209990 | LEP (+29922) | chr7 | 0.682 | 0.630 | -0.052 | 0.0289 |
| 422 | cg13518079 | EBF4 (+1549) | chr20 | 0.438 | 0.386 | -0.052 | 0.0205 |
| 423 | cg17241353 | CBLN4 (+712513) | chr20 | 0.672 | 0.620 | -0.052 | 0.0026 |
| 424 | cg02663317 | ARNTL2 (-40043) | chr12 | 0.671 | 0.619 | -0.053 | 0.0018 |
| 425 | cg18193195 | SUOX (-10774) | chr12 | 0.709 | 0.657 | -0.053 | 0.0237 |
| 426 | cg22027267 | COBL (-160023) | chr7 | 0.749 | 0.696 | -0.053 | 0.0047 |
| 427 | cg11546683 | CPPED1 (+187159) | chr16 | 0.787 | 0.734 | -0.053 | 0.0140 |
| 428 | cg22079902 | PRDM9 (-80) | chr5 | 0.592 | 0.539 | -0.053 | 0.0193 |
| 429 | cg19162470 | FAN1 (-3185) | chr15 | 0.663 | 0.610 | -0.053 | 0.0170 |
| 430 | cg22762992 | DDX18 (+44152) | chr2 | 0.657 | 0.604 | -0.053 | 0.0384 |
| 431 | cg09209787 | PROX1 (-10179) | chr1 | 0.519 | 0.466 | -0.053 | 0.0015 |
| 432 | cg03971051 | KLHL35 (-11281) | chr11 | 0.816 | 0.762 | -0.053 | 0.0280 |
| 433 | cg01066472 | LHX8 (-3090) | chr1 | 0.431 | 0.378 | -0.053 | 0.0216 |
| 434 | cg24920126 | RPP40 (-82986) | chr6 | 0.693 | 0.639 | -0.053 | 0.0167 |
| 435 | cg15846771 | ZDHHC11 (-4165) | chr5 | 0.542 | 0.488 | -0.053 | 0.0039 |
| 436 | cg07531550 | FRG1 (-209348) | chr4 | 0.661 | 0.607 | -0.053 | 0.0402 |
| 437 | cg21556998 | STRBP (+44032) | chr9 | 0.686 | 0.632 | -0.054 | 0.0003 |
| 438 | cg20304530 | NTRK3 (+297163) | chr15 | 0.801 | 0.747 | -0.054 | 0.0018 |
| 439 | cg13476778 | FRMD6 (-1261) | chr14 | 0.555 | 0.501 | -0.054 | 0.0191 |
| 440 | cg14771877 | C17orf99 (-1396) | chr17 | 0.712 | 0.658 | -0.054 | 0.0133 |
| 441 | cg18211633 | RHOF (+26559) | chr12 | 0.669 | 0.615 | -0.054 | 0.0081 |
| 442 | cg20660297 | KRR1 (-903) | chr12 | 0.538 | 0.484 | -0.054 | 0.0169 |
| 443 | cg09053901 | SHANK2 (+8174) | chr11 | 0.821 | 0.767 | -0.054 | 0.0041 |
| 444 | cg05010260 | NAT16 (+3266) | chr7 | 0.615 | 0.561 | -0.054 | 0.0054 |
| 445 | cg27585222 | ACTR3 (+90194) | chr2 | 0.649 | 0.595 | -0.054 | 0.0108 |
| 446 | cg04678955 | DEFB115 (-321336) | chr20 | 0.512 | 0.458 | -0.054 | 0.0336 |
| 447 | cg00561395 | ATXN10 (+46358) | chr22 | 0.693 | 0.638 | -0.054 | 0.0045 |
| 448 | cg17850273 | KIF21A (+74994) | chr12 | 0.649 | 0.595 | -0.054 | 0.0049 |
| 449 | cg01540595 | LHX8 (-3159) | chr1 | 0.474 | 0.420 | -0.054 | 0.0071 |
| 450 | cg03779490 | SNX9 (-174754) | chr6 | 0.551 | 0.496 | -0.055 | 0.0162 |
| 451 | cg00590063 | B3GNT3 (+12877) | chr19 | 0.332 | 0.277 | -0.055 | 0.0255 |
| 452 | cg03499324 | PSMD5 (-858) | chr9 | 0.741 | 0.686 | -0.055 | 0.0352 |
| 453 | cg00105415 | EGR2 (-3162) | chr10 | 0.669 | 0.615 | -0.055 | 0.0021 |
| 454 | cg09048334 | FGD2 (+39218) | chr6 | 0.432 | 0.377 | -0.055 | 0.0455 |
| 455 | cg23425970 | HS6ST1 (-244) | chr2 | 0.242 | 0.187 | -0.055 | 0.0172 |
| 456 | cg15620114 | SLC16A12 (-1145) | chr10 | 0.768 | 0.713 | -0.055 | 0.0053 |
| 457 | cg01799458 | HOXB13 (+1736) | chr17 | 0.573 | 0.518 | -0.055 | 0.0048 |
| 458 | cg25890575 | DNAJB6 (+240283) | chr7 | 0.788 | 0.733 | -0.055 | 0.0343 |
| 459 | cg02655397 | TMEM262 (-3138) | chr11 | 0.731 | 0.675 | -0.056 | 0.0009 |
| 460 | cg11016221 | PTGES (+9679) | chr9 | 0.415 | 0.359 | -0.056 | 0.0123 |
| 461 | cg05874329 | CYP11B1 (+17565) | chr8 | 0.625 | 0.569 | -0.056 | 0.0018 |
| 462 | cg13375654 | DOC2A (-1283) | chr16 | 0.454 | 0.398 | -0.056 | 0.0084 |
| 463 | cg05663294 | DUSP10 (-236921) | chr1 | 0.594 | 0.538 | -0.056 | 0.0027 |
| 464 | cg16506114 | EPHA6 (-37633) | chr3 | 0.638 | 0.581 | -0.057 | 0.0020 |
| 465 | cg14551984 | SGOL2 (+8923) | chr2 | 0.726 | 0.669 | -0.057 | 0.0018 |
| 466 | cg05208483 | CSPG4 (+30148) | chr15 | 0.797 | 0.740 | -0.057 | 0.0362 |
| 467 | cg11206167 | SEPP1 (-112187) | chr5 | 0.767 | 0.710 | -0.057 | 0.0328 |
| 468 | cg08310558 | SLFN13 (-10935) | chr17 | 0.851 | 0.794 | -0.057 | 0.0233 |
| 469 | cg05343328 | ING1 (+98215) | chr13 | 0.649 | 0.593 | -0.057 | 0.0172 |
| 470 | cg16603012 | APRT (-1242) | chr16 | 0.658 | 0.602 | -0.057 | 0.0195 |
| 471 | cg20336352 | HERC4 (-13229) | chr10 | 0.761 | 0.704 | -0.057 | 0.0157 |
| 472 | cg08145067 | PIP5K1C (+12301) | chr19 | 0.329 | 0.272 | -0.057 | 0.0112 |
| 473 | cg03264585 | MTRNR2L1 (-229467) | chr17 | 0.646 | 0.589 | -0.057 | 0.0031 |
| 474 | cg02996355 | STON2 (+14373) | chr14 | 0.278 | 0.221 | -0.057 | 0.0105 |
| 475 | cg00816676 | MTRNR2L7 (-78458) | chr10 | 0.731 | 0.674 | -0.057 | 0.0018 |
| 476 | cg02661831 | PHF3 (+41346) | chr6 | 0.676 | 0.619 | -0.057 | 0.0105 |
| 477 | cg26137217 | TAF4 (+309977) | chr20 | 0.600 | 0.543 | -0.057 | 0.0090 |
| 478 | cg08122831 | ERBB4 (-294015) | chr2 | 0.770 | 0.713 | -0.057 | 0.0002 |
| 479 | cg18844029 | TRIM27 (+6749) | chr6 | 0.753 | 0.696 | -0.057 | 0.0131 |
| 480 | cg08124399 | DDX43 (+398) | chr6 | 0.733 | 0.676 | -0.057 | 0.0257 |
| 481 | cg10831285 | B3GNT3 (+13009) | chr19 | 0.319 | 0.261 | -0.058 | 0.0009 |
| 482 | cg11643285 | OXNAD1 (+104962) | chr3 | 0.814 | 0.757 | -0.058 | 0.0042 |
| 483 | cg24727089 | TXNL1 (+46082) | chr18 | 0.663 | 0.606 | -0.058 | 0.0082 |
| 484 | cg27516945 | ZNF528 (-603) | chr19 | 0.673 | 0.616 | -0.058 | 0.0114 |
| 485 | cg14361252 | ING1 (+98129) | chr13 | 0.593 | 0.536 | -0.058 | 0.0223 |
| 486 | cg05275595 | TEX101 (-47774) | chr19 | 0.374 | 0.316 | -0.058 | 0.0430 |
| 487 | cg07938212 | BICD1 (+13201) | chr12 | 0.637 | 0.579 | -0.058 | 0.0007 |
| 488 | cg06968712 | MS4A15 (+10431) | chr11 | 0.558 | 0.500 | -0.058 | 0.0059 |
| 489 | cg15199034 | ADAMTS1 (-42681) | chr21 | 0.705 | 0.647 | -0.058 | 0.0024 |
| 490 | cg09602542 | GGT6 (-36137) | chr17 | 0.839 | 0.780 | -0.059 | 0.0025 |
| 491 | cg18423549 | MTRNR2L1 (-278559) | chr17 | 0.398 | 0.339 | -0.059 | 0.0210 |
| 492 | cg06650819 | ACTL6A (+2784) | chr3 | 0.495 | 0.436 | -0.059 | 0.0309 |
| 493 | cg04272684 | OVCH1 (-21212) | chr12 | 0.676 | 0.617 | -0.059 | 0.0173 |
| 494 | cg27393610 | TMEM116 (+6954) | chr12 | 0.889 | 0.831 | -0.059 | 0.0015 |
| 495 | cg17240725 | ABHD11 (+3841) | chr7 | 0.398 | 0.339 | -0.059 | 0.0110 |
| 496 | cg16294255 | MMEL1 (+1855) | chr1 | 0.507 | 0.448 | -0.059 | 0.0057 |
| 497 | cg03116837 | ZNF596 (+34320) | chr8 | 0.453 | 0.394 | -0.059 | 0.0257 |
| 498 | cg20493661 | KIR3DL1 (+15456) | chr19 | 0.590 | 0.531 | -0.059 | 0.0093 |
| 499 | cg02878510 | SERTAD4 (-32407) | chr1 | 0.654 | 0.595 | -0.059 | 0.0063 |
| 500 | cg03218402 | TAS2R13 (+26087) | chr12 | 0.818 | 0.759 | -0.059 | 0.0196 |
| 501 | cg06015275 | HSBP1 (-66549) | chr16 | 0.603 | 0.544 | -0.059 | 0.0175 |
| 502 | cg27427514 | HES1 (+68104) | chr3 | 0.199 | 0.139 | -0.060 | 0.0083 |
| 503 | cg25122629 | EML6 (-14857) | chr2 | 0.366 | 0.306 | -0.060 | 0.0045 |
| 504 | cg13729548 | CHGA (+16513) | chr14 | 0.742 | 0.682 | -0.060 | 0.0204 |
| 505 | cg04601780 | ENSG00000264813 (+10162) | chr17 | 0.474 | 0.413 | -0.060 | 0.0182 |
| 506 | cg06520296 | ZNF703 (-202288) | chr8 | 0.569 | 0.509 | -0.060 | 0.0155 |
| 507 | cg14554415 | SPESP1 (+150) | chr15 | 0.636 | 0.576 | -0.060 | 0.0107 |
| 508 | cg06513015 | ERV3-1 (+7785) | chr7 | 0.784 | 0.723 | -0.060 | 0.0016 |
| 509 | cg07256649 | HOPX (+68186) | chr4 | 0.828 | 0.768 | -0.060 | 0.0408 |
| 510 | cg08030235 | EEPD1 (-95569) | chr7 | 0.726 | 0.665 | -0.060 | 0.0104 |
| 511 | cg05471900 | IL1F10 (-1191) | chr2 | 0.704 | 0.643 | -0.061 | 0.0035 |
| 512 | cg00167275 | GLUD1 (+35) | chr10 | 0.165 | 0.104 | -0.061 | 0.0008 |
| 513 | cg10846356 | CALCB (-726) | chr11 | 0.643 | 0.581 | -0.061 | 0.0042 |
| 514 | cg15936068 | DDX43 (+407) | chr6 | 0.751 | 0.689 | -0.061 | 0.0312 |
| 515 | cg18503693 | FAM155A (+595468) | chr13 | 0.623 | 0.561 | -0.062 | 0.0131 |
| 516 | cg10491108 | TST (+6245) | chr22 | 0.645 | 0.583 | -0.062 | 0.0384 |
| 517 | cg22647161 | TMPO (-223043) | chr12 | 0.595 | 0.533 | -0.062 | 0.0017 |
| 518 | cg26111308 | TVP23B (+14743) | chr17 | 0.524 | 0.462 | -0.062 | 0.0173 |
| 519 | cg00464738 | HLA-G (+35493) | chr6 | 0.784 | 0.722 | -0.062 | 0.0009 |
| 520 | cg10432947 | NAT8L (+1203) | chr4 | 0.583 | 0.521 | -0.062 | 0.0061 |
| 521 | cg09430341 | CCDC17 (-481) | chr1 | 0.766 | 0.703 | -0.063 | 0.0054 |
| 522 | cg08617160 | MIER2 (-520) | chr19 | 0.537 | 0.475 | -0.063 | 0.0058 |
| 523 | cg22871949 | NOM1 (-6814) | chr7 | 0.641 | 0.578 | -0.063 | 0.0007 |
| 524 | cg22545206 | DDX18 (+45274) | chr2 | 0.523 | 0.460 | -0.063 | 0.0084 |
| 525 | cg07442889 | SIGLEC5 (-914) | chr19 | 0.698 | 0.635 | -0.063 | 0.0207 |
| 526 | cg12060669 | R3HCC1 (+33863) | chr8 | 0.323 | 0.259 | -0.064 | 0.0443 |
| 527 | cg17014647 | GMPR (+192496) | chr6 | 0.603 | 0.539 | -0.064 | 0.0011 |
| 528 | cg22599254 | BCAR3 (+126150) | chr1 | 0.863 | 0.799 | -0.064 | 0.0027 |
| 529 | cg09419670 | PSMD5 (-405) | chr9 | 0.215 | 0.151 | -0.064 | 0.0388 |
| 530 | cg06940110 | DDX18 (+44205) | chr2 | 0.498 | 0.434 | -0.064 | 0.0201 |
| 531 | cg11092486 | RPP40 (-83324) | chr6 | 0.547 | 0.482 | -0.064 | 0.0252 |
| 532 | cg17014757 | CHI3L1 (-221) | chr1 | 0.398 | 0.334 | -0.064 | 0.0256 |
| 533 | cg15838333 | PCNXL4 (-4189) | chr14 | 0.684 | 0.619 | -0.065 | 0.0211 |
| 534 | cg05915609 | PRSS22 (+3716) | chr16 | 0.650 | 0.585 | -0.065 | 0.0105 |
| 535 | cg22526555 | KCNJ15 (-49635) | chr21 | 0.774 | 0.709 | -0.065 | 0.0026 |
| 536 | cg26011615 | BATF3 (+34020) | chr1 | 0.692 | 0.627 | -0.065 | 0.0003 |
| 537 | cg19708055 | C6orf123 (+151983) | chr6 | 0.644 | 0.578 | -0.065 | 0.0027 |
| 538 | cg14248883 | SERP2 (+30700) | chr13 | 0.739 | 0.674 | -0.065 | 0.0053 |
| 539 | cg10852875 | IRX2 (+214079) | chr5 | 0.586 | 0.521 | -0.066 | 0.0030 |
| 540 | cg08219700 | IMPAD1 (-149624) | chr8 | 0.485 | 0.419 | -0.066 | 0.0201 |
| 541 | cg10292709 | MCPH1 (-435562) | chr8 | 0.719 | 0.653 | -0.066 | 0.0091 |
| 542 | cg00073565 | ZFPM2 (+131789) | chr8 | 0.562 | 0.496 | -0.066 | 0.0279 |
| 543 | cg03994477 | FKBP4 (-75225) | chr12 | 0.556 | 0.489 | -0.066 | 0.0007 |
| 544 | cg27565337 | RASA3 (+42024) | chr13 | 0.543 | 0.476 | -0.067 | 0.0274 |
| 545 | cg04580344 | KIAA0408 (-16487) | chr6 | 0.728 | 0.661 | -0.067 | 0.0053 |
| 546 | cg06068545 | SMYD2 (+81413) | chr1 | 0.161 | 0.094 | -0.067 | 0.0219 |
| 547 | cg15634083 | HLA-B (+86056) | chr6 | 0.203 | 0.136 | -0.067 | 0.0041 |
| 548 | cg23441030 | CACYBP (-59405) | chr1 | 0.716 | 0.648 | -0.067 | 0.0001 |
| 549 | cg06143315 | CXXC11 (+138431) | chr2 | 0.769 | 0.702 | -0.067 | 0.0201 |
| 550 | cg19097082 | ZBTB44 (-16742) | chr11 | 0.732 | 0.664 | -0.068 | 0.0024 |
| 551 | cg10907232 | FAM49B (+44722) | chr8 | 0.790 | 0.722 | -0.068 | 0.0012 |
| 552 | cg16048001 | PPP1R12B (+30298) | chr1 | 0.787 | 0.719 | -0.068 | 0.0017 |
| 553 | cg10756719 | NKX6-1 (+118018) | chr4 | 0.736 | 0.668 | -0.068 | 0.0001 |
| 554 | cg08557536 | ZNF112 (-689) | chr19 | 0.516 | 0.447 | -0.069 | 0.0045 |
| 555 | cg09798387 | DEFB115 (-321358) | chr20 | 0.382 | 0.313 | -0.069 | 0.0059 |
| 556 | cg04974804 | HTR1D (-594) | chr1 | 0.733 | 0.664 | -0.070 | 0.0022 |
| 557 | cg02603861 | NONE | chr14 | 0.418 | 0.348 | -0.070 | 0.0053 |
| 558 | cg00783857 | SYT2 (+104642) | chr1 | 0.782 | 0.712 | -0.070 | 0.0015 |
| 559 | cg18174881 | MIS18BP1 (-749) | chr14 | 0.710 | 0.640 | -0.070 | 0.0006 |
| 560 | cg02825122 | ZHX2 (-97192) | chr8 | 0.784 | 0.714 | -0.071 | 0.0139 |
| 561 | cg20478239 | COBL (-159980) | chr7 | 0.696 | 0.625 | -0.071 | 0.0044 |
| 562 | cg12011299 | ADH4 (-99) | chr4 | 0.609 | 0.537 | -0.071 | 0.0121 |
| 563 | cg11494091 | GH2 (-233) | chr17 | 0.711 | 0.640 | -0.072 | 0.0156 |
| 564 | cg03804706 | SYT2 (+115163) | chr1 | 0.775 | 0.703 | -0.072 | 0.0007 |
| 565 | cg00191853 | SPAG1 (+7078) | chr8 | 0.506 | 0.434 | -0.072 | 0.0277 |
| 566 | cg27562174 | ADH4 (+2) | chr4 | 0.740 | 0.667 | -0.073 | 0.0203 |
| 567 | cg06083423 | RHOU (+244657) | chr1 | 0.690 | 0.617 | -0.073 | 0.0112 |
| 568 | cg05056638 | NEFM (+30300) | chr8 | 0.595 | 0.522 | -0.073 | 0.0026 |
| 569 | cg05697751 | DUSP16 (+4582) | chr12 | 0.786 | 0.713 | -0.073 | 0.0056 |
| 570 | cg17765025 | SUCLG1 (+581435) | chr2 | 0.747 | 0.674 | -0.073 | 0.0027 |
| 571 | cg24008280 | PLD5 (-288775) | chr1 | 0.643 | 0.569 | -0.073 | 0.0213 |
| 572 | cg09027495 | TBC1D2B (+210643) | chr15 | 0.735 | 0.661 | -0.073 | 0.0022 |
| 573 | cg04603290 | TNFSF14 (+9947) | chr19 | 0.624 | 0.550 | -0.074 | 0.0017 |
| 574 | cg06489993 | CALM3 (-21497) | chr19 | 0.640 | 0.566 | -0.074 | 0.0190 |
| 575 | cg21720175 | MAF (-53056) | chr16 | 0.537 | 0.462 | -0.074 | 0.0343 |
| 576 | cg14393316 | CAMSAP1 (+86740) | chr9 | 0.780 | 0.705 | -0.075 | 0.0010 |
| 577 | cg13912224 | MCM3 (-22449) | chr6 | 0.497 | 0.422 | -0.075 | 0.0386 |
| 578 | cg18325044 | HLA-G (+74145) | chr6 | 0.814 | 0.739 | -0.076 | 0.0051 |
| 579 | cg16270721 | EXOSC5 (-9704) | chr19 | 0.401 | 0.325 | -0.076 | 0.0361 |
| 580 | cg11061773 | TSC22D2 (-156570) | chr3 | 0.760 | 0.683 | -0.076 | 0.0188 |
| 581 | cg11685843 | ADAM29 (+509663) | chr4 | 0.628 | 0.551 | -0.076 | 0.0015 |
| 582 | cg07569483 | NLRP9 (-8089) | chr19 | 0.638 | 0.562 | -0.076 | 0.0388 |
| 583 | cg01813171 | HLA-G (-32746) | chr6 | 0.720 | 0.643 | -0.077 | 0.0173 |
| 584 | cg08414882 | BCMO1 (-15730) | chr16 | 0.595 | 0.517 | -0.077 | 0.0095 |
| 585 | cg03911306 | DAZL (-1998) | chr3 | 0.727 | 0.650 | -0.077 | 0.0021 |
| 586 | cg01880147 | FCGR3B (-8389) | chr1 | 0.706 | 0.628 | -0.078 | 0.0264 |
| 587 | cg22629375 | OSCAR (+6096) | chr19 | 0.539 | 0.461 | -0.078 | 0.0362 |
| 588 | cg10661558 | LIPI (+136111) | chr21 | 0.732 | 0.654 | -0.078 | 0.0002 |
| 589 | cg24480926 | SFSWAP (-276103) | chr12 | 0.633 | 0.555 | -0.078 | 0.0002 |
| 590 | cg24213669 | DDX18 (+44351) | chr2 | 0.472 | 0.393 | -0.079 | 0.0029 |
| 591 | cg16324669 | CDK11A (-8318) | chr1 | 0.847 | 0.768 | -0.079 | 0.0448 |
| 592 | cg17056703 | SYCP2L (+65575) | chr6 | 0.792 | 0.712 | -0.079 | 0.0133 |
| 593 | cg07753967 | DEFB115 (-233815) | chr20 | 0.287 | 0.208 | -0.080 | 0.0401 |
| 594 | cg03787837 | HLA-DQA1 (+252) | chr6 | 0.477 | 0.397 | -0.080 | 0.0306 |
| 595 | cg20811988 | DEFB115 (-233543) | chr20 | 0.273 | 0.193 | -0.080 | 0.0118 |
| 596 | cg18220841 | BANP (+156623) | chr16 | 0.553 | 0.473 | -0.080 | 0.0236 |
| 597 | cg22223119 | STK24 (+78568) | chr13 | 0.542 | 0.462 | -0.080 | 0.0080 |
| 598 | cg07195891 | CLEC4C (+1997) | chr12 | 0.729 | 0.648 | -0.080 | 0.0117 |
| 599 | cg10871684 | PLB1 (-44639) | chr2 | 0.859 | 0.779 | -0.081 | 0.0147 |
| 600 | cg22445217 | QKI (+585402) | chr6 | 0.695 | 0.614 | -0.081 | 0.0104 |
| 601 | cg05758861 | BAI1 (+50033) | chr8 | 0.754 | 0.673 | -0.081 | 0.0001 |
| 602 | cg22744079 | PLRG1 (+60727) | chr4 | 0.614 | 0.532 | -0.081 | 0.0009 |
| 603 | cg15563854 | TLE1 (-59) | chr9 | 0.200 | 0.118 | -0.082 | 0.0004 |
| 604 | cg20390711 | KIAA0408 (-16148) | chr6 | 0.733 | 0.651 | -0.083 | 0.0009 |
| 605 | cg14079463 | KIAA0408 (-16454) | chr6 | 0.563 | 0.481 | -0.083 | 0.0227 |
| 606 | cg09829303 | CALD1 (-44627) | chr7 | 0.674 | 0.591 | -0.083 | 0.0004 |
| 607 | cg09516963 | DYRK2 (-56) | chr12 | 0.320 | 0.237 | -0.083 | 0.0017 |
| 608 | cg22457256 | AZGP1 (-1085) | chr7 | 0.662 | 0.579 | -0.083 | 0.0010 |
| 609 | cg22032020 | MEF2A (+15666) | chr15 | 0.721 | 0.637 | -0.083 | 0.0420 |
| 610 | cg13469777 | ZNRD1 (-50410) | chr6 | 0.676 | 0.591 | -0.085 | 0.0141 |
| 611 | cg06451157 | HLA-G (+73540) | chr6 | 0.795 | 0.709 | -0.085 | 0.0013 |
| 612 | cg02783661 | KDM5A (-3573) | chr12 | 0.614 | 0.529 | -0.085 | 0.0117 |
| 613 | cg24433124 | IER3 (-43638) | chr6 | 0.611 | 0.524 | -0.086 | 0.0383 |
| 614 | cg04692312 | DEFB115 (-326651) | chr20 | 0.278 | 0.191 | -0.087 | 0.0028 |
| 615 | cg18349077 | TMEM87B (+30103) | chr2 | 0.524 | 0.436 | -0.088 | 0.0122 |
| 616 | cg23904161 | ING1 (+98212) | chr13 | 0.542 | 0.452 | -0.090 | 0.0135 |
| 617 | cg27286337 | NKX6-2 (+44276) | chr10 | 0.607 | 0.518 | -0.090 | 0.0327 |
| 618 | cg19729930 | BOLA3 (+17249) | chr2 | 0.599 | 0.509 | -0.090 | 0.0096 |
| 619 | cg10805896 | PLXNA4 (-88990) | chr7 | 0.444 | 0.354 | -0.090 | 0.0033 |
| 620 | cg13139335 | C8orf37 (-333487) | chr8 | 0.465 | 0.374 | -0.091 | 0.0173 |
| 621 | cg11581472 | SLC25A12 (+83511) | chr2 | 0.767 | 0.675 | -0.091 | 0.0014 |
| 622 | cg20926353 | TLE1 (+862) | chr9 | 0.299 | 0.206 | -0.093 | 0.0007 |
| 623 | cg10804687 | HLA-G (+64765) | chr6 | 0.860 | 0.766 | -0.094 | 0.0061 |
| 624 | cg00063654 | OXNAD1 (+106372) | chr3 | 0.712 | 0.618 | -0.094 | 0.0003 |
| 625 | cg22425359 | IRX2 (+335898) | chr5 | 0.810 | 0.716 | -0.095 | 0.0061 |
| 626 | cg22945019 | RASGRP3 (-174836) | chr2 | 0.739 | 0.644 | -0.095 | 0.0263 |
| 627 | cg01966510 | ZSCAN2 (-68809) | chr15 | 0.619 | 0.523 | -0.096 | 0.0007 |
| 628 | cg01017244 | BOLA3 (+17594) | chr2 | 0.720 | 0.624 | -0.096 | 0.0037 |
| 629 | cg10892585 | ZNF138 (+43878) | chr7 | 0.773 | 0.676 | -0.097 | 0.0012 |
| 630 | cg23024343 | DUS4L (-2653) | chr7 | 0.605 | 0.508 | -0.097 | 0.0321 |
| 631 | cg12060786 | HSPA1B (+8207) | chr6 | 0.267 | 0.170 | -0.097 | 0.0184 |
| 632 | cg10474018 | HLA-G (+65261) | chr6 | 0.873 | 0.775 | -0.098 | 0.0011 |
| 633 | cg00399683 | DPP6 (-640390) | chr7 | 0.698 | 0.600 | -0.098 | 0.0027 |
| 634 | cg15514307 | PPP2CA (-20989) | chr5 | 0.746 | 0.647 | -0.099 | 0.0185 |
| 635 | cg20891558 | BOLA3 (+17270) | chr2 | 0.569 | 0.470 | -0.099 | 0.0141 |
| 636 | cg21717724 | PSMD5 (+748) | chr9 | 0.604 | 0.504 | -0.099 | 0.0138 |
| 637 | cg26127187 | HLA-G (+62040) | chr6 | 0.810 | 0.710 | -0.100 | 0.0008 |
| 638 | cg11955727 | SUCLG1 (+581058) | chr2 | 0.783 | 0.683 | -0.100 | 0.0007 |
| 639 | cg06454464 | TSNARE1 (+56622) | chr8 | 0.834 | 0.733 | -0.101 | 0.0288 |
| 640 | cg19899561 | BDH2 (-38226) | chr4 | 0.832 | 0.731 | -0.102 | 0.0213 |
| 641 | cg14018363 | HLA-G (+116510) | chr6 | 0.509 | 0.407 | -0.102 | 0.0044 |
| 642 | cg15482884 | PNO1 (+38423) | chr2 | 0.576 | 0.474 | -0.103 | 0.0198 |
| 643 | cg12949927 | ZNF138 (+43874) | chr7 | 0.750 | 0.647 | -0.103 | 0.0009 |
| 644 | cg00157199 | DEFB115 (-293845) | chr20 | 0.439 | 0.336 | -0.104 | 0.0037 |
| 645 | cg26844603 | TMEM87B (+30108) | chr2 | 0.631 | 0.528 | -0.104 | 0.0107 |
| 646 | cg14815891 | DEFB115 (-233564) | chr20 | 0.290 | 0.186 | -0.104 | 0.0217 |
| 647 | cg20578893 | HLA-G (+75305) | chr6 | 0.774 | 0.669 | -0.105 | 0.0005 |
| 648 | cg04057469 | RFTN1 (+82369) | chr3 | 0.710 | 0.604 | -0.106 | 0.0001 |
| 649 | cg19077165 | TCEB3CL2 (-2555) | chr18 | 0.747 | 0.640 | -0.107 | 0.0002 |
| 650 | cg26649688 | HLA-G (+63605) | chr6 | 0.883 | 0.776 | -0.107 | 0.0004 |
| 651 | cg26964592 | HLA-DMB (+4226) | chr6 | 0.566 | 0.457 | -0.109 | 0.0046 |
| 652 | cg18423635 | HLA-G (+75181) | chr6 | 0.855 | 0.746 | -0.109 | 0.0003 |
| 653 | cg09104915 | SHANK2 (-8851) | chr11 | 0.632 | 0.520 | -0.112 | 0.0020 |
| 654 | cg03126799 | R3HCC1 (+33566) | chr8 | 0.657 | 0.545 | -0.112 | 0.0254 |
| 655 | cg24819596 | ST3GAL5 (-18877) | chr2 | 0.765 | 0.649 | -0.116 | 0.0008 |
| 656 | cg09670175 | KCNA6 (+5127) | chr12 | 0.734 | 0.618 | -0.116 | 0.0230 |
| 657 | cg12182020 | NONE | chr3 | 0.415 | 0.298 | -0.117 | 0.0297 |
| 658 | cg15228509 | CEP170 (+343635) | chr1 | 0.573 | 0.454 | -0.119 | 0.0008 |
| 659 | cg24179288 | HLA-G (+72530) | chr6 | 0.866 | 0.747 | -0.119 | 0.0002 |
| 660 | cg00151744 | AKAP13 (-32444) | chr15 | 0.576 | 0.456 | -0.120 | 0.0012 |
| 661 | cg03395495 | GOLGA6L4 (-83129) | chr15 | 0.709 | 0.586 | -0.123 | 0.0000 |
| 662 | cg15825968 | SFSWAP (-276155) | chr12 | 0.618 | 0.494 | -0.125 | 0.0000 |
| 663 | cg12927252 | UPP2 (+18697) | chr2 | 0.715 | 0.590 | -0.126 | 0.0305 |
| 664 | cg20381372 | ZFP14 (+68693) | chr19 | 0.574 | 0.448 | -0.127 | 0.0266 |
| 665 | cg17232014 | HEBP1 (+14) | chr12 | 0.310 | 0.183 | -0.127 | 0.0006 |
| 666 | cg00727777 | MYOM2 (+344659) | chr8 | 0.612 | 0.485 | -0.128 | 0.0048 |
| 667 | cg12046183 | HLA-G (+65074) | chr6 | 0.727 | 0.597 | -0.130 | 0.0013 |
| 668 | cg13149459 | PPP1R12B (+107853) | chr1 | 0.753 | 0.622 | -0.131 | 0.0005 |
| 669 | cg25817503 | AFAP1 (+153311) | chr4 | 0.599 | 0.466 | -0.133 | 0.0054 |
| 670 | cg25343008 | SYT2 (+142351) | chr1 | 0.701 | 0.564 | -0.137 | 0.0004 |
| 671 | cg05890377 | BOLA3 (+17408) | chr2 | 0.601 | 0.461 | -0.140 | 0.0014 |
| 672 | cg13685349 | STON2 (+14501) | chr14 | 0.736 | 0.586 | -0.151 | 0.0047 |
| 673 | cg17939448 | FAM47E-STBD1 (-26347) | chr4 | 0.517 | 0.360 | -0.157 | 0.0043 |
| 674 | cg26919182 | SYT2 (+90349) | chr1 | 0.720 | 0.541 | -0.179 | 0.0004 |
| 675 | cg13393919 | GPBAR1 (-10899) | chr2 | 0.791 | 0.605 | -0.186 | 0.0000 |
| 676 | cg04462931 | ZNF138 (+45259) | chr7 | 0.690 | 0.502 | -0.188 | 0.0006 |

**Table S7.** Differentially methylated regions in whole blood of people with hyperuricemia compared to normouricemic people without cell composition correction

| Nr. | nearest gene | DMR chr | DMR start | DMR end | Length | probes in dmr |
| --- | --- | --- | --- | --- | --- | --- |
| 1 | HLA-G | chr6 | 29855325 | 29858360 | 3036 | 34 |
| 2 | HLA-G | chr6 | 29893273 | 29895204 | 1932 | 33 |
| 3 | HLA-B | chr6 | 31238388 | 31239411 | 1024 | 16 |
| 4 | HLA-G | chr6 | 29910525 | 29911550 | 1026 | 14 |
| 5 | FAM90A1 | chr12 | 8380001 | 8381021 | 1021 | 11 |
| 6 | SPACA7 | chr13 | 112984602 | 112986285 | 1684 | 11 |
| 7 | LPCAT1 | chr5 | 1594021 | 1595048 | 1028 | 11 |
| 8 | C1orf65 | chr1 | 223566268 | 223567002 | 735 | 10 |
| 9 | CLDN14 | chr21 | 37915044 | 37915391 | 348 | 10 |
| 10 | WRB | chr21 | 40759534 | 40760975 | 1442 | 10 |
| 11 | PRDM9 | chr5 | 23507243 | 23507752 | 510 | 10 |
| 12 | CCNH | chr5 | 86708832 | 86709603 | 772 | 10 |
| 13 | TLE1 | chr9 | 84303358 | 84304983 | 1626 | 10 |
| 14 | HLA-G | chr6 | 29795350 | 29795595 | 246 | 9 |
| 15 | GLUD1 | chr10 | 88853608 | 88854588 | 981 | 8 |
| 16 | SPESP1 | chr15 | 69222400 | 69223368 | 969 | 8 |
| 17 | MTHFS | chr15 | 80189694 | 80190344 | 651 | 8 |
| 18 | KIAA0408 | chr6 | 127796287 | 127797286 | 1000 | 7 |
| 19 | PLCH2 | chr1 | 2390701 | 2391837 | 1137 | 6 |
| 20 | FBRSL1 | chr12 | 132903921 | 132904796 | 876 | 6 |
| 21 | BCL2A1 | chr15 | 80263132 | 80263923 | 792 | 6 |
| 22 | IGF2BP1 | chr17 | 47091521 | 47092272 | 752 | 6 |
| 23 | SOCS3 | chr17 | 76354621 | 76355288 | 668 | 6 |
| 24 | SUCLG1 | chr2 | 84105169 | 84105744 | 576 | 6 |
| 25 | ANKRD23 | chr2 | 97505275 | 97505787 | 513 | 6 |
| 26 | DDX18 | chr2 | 118616155 | 118617230 | 1076 | 6 |
| 27 | FXR1 | chr3 | 180587900 | 180588228 | 329 | 6 |
| 28 | HLA-G | chr6 | 29868295 | 29870060 | 1766 | 6 |
| 29 | POU6F2 | chr7 | 39170497 | 39171113 | 617 | 6 |
| 30 | DNAJB6 | chr7 | 157405965 | 157406737 | 773 | 6 |
| 31 | EGR2 | chr10 | 64579032 | 64579646 | 615 | 5 |
| 32 | BOLA3 | chr2 | 74357527 | 74358223 | 697 | 5 |
| 33 | USP16 | chr21 | 30395808 | 30396586 | 779 | 5 |
| 34 | C6orf123 | chr6 | 168045268 | 168045888 | 621 | 5 |
| 35 | ZNF12 | chr7 | 6746799 | 6747037 | 239 | 5 |
| 36 | NOM1 | chr7 | 156735260 | 156735656 | 397 | 5 |
| 37 | BAI1 | chr8 | 143580770 | 143581481 | 712 | 5 |
| 38 | CEP170 | chr1 | 243053673 | 243054071 | 399 | 4 |
| 39 | MTRNR2L7 | chr10 | 37969553 | 37970316 | 764 | 4 |
| 40 | TAGLN | chr11 | 117069849 | 117070046 | 198 | 4 |
| 41 | NPIPB11 | chr16 | 29296186 | 29296797 | 612 | 4 |
| 42 | HOXB5 | chr17 | 46675892 | 46676375 | 484 | 4 |
| 43 | NOBOX | chr7 | 144107418 | 144107626 | 209 | 4 |
| 44 | CNTNAP3B | chr9 | 44401998 | 44402433 | 436 | 4 |
| 45 | KCNN3 | chr1 | 154839813 | 154839983 | 171 | 3 |
| 46 | TTC40 | chr10 | 134778286 | 134778648 | 363 | 3 |
| 47 | CALCB | chr11 | 15094338 | 15094382 | 45 | 3 |
| 48 | SFSWAP | chr12 | 131919527 | 131919784 | 258 | 3 |
| 49 | B3GNT3 | chr19 | 17918795 | 17919173 | 379 | 3 |
| 50 | ERBB4 | chr2 | 213697579 | 213698158 | 580 | 3 |
| 51 | CXXC11 | chr2 | 242843821 | 242844174 | 354 | 3 |
| 52 | BRD1 | chr22 | 50098074 | 50098317 | 244 | 3 |
| 53 | NAT8L | chr4 | 2062392 | 2063036 | 645 | 3 |
| 54 | HIST1H2AD | chr6 | 26196580 | 26196794 | 215 | 3 |
| 55 | HLA-G | chr6 | 29859520 | 29860016 | 497 | 3 |
